# Design, Synthesis and Biological Activity Study of γ-Aminobutyric Acid (GABA) Derivatives Containing Bridged Bicyclic Skeletons as BCAT1 Inhibitors

**DOI:** 10.3390/molecules30040904

**Published:** 2025-02-15

**Authors:** Wen Luo, Zilu Pan, Xinyuan Zhu, Yan Li, Yong Li, Yudi Zhang, Jiamin Pan, Jian Ding, Hua Xie, Guilong Zhao

**Affiliations:** 1 School of Pharmaceutical Sciences, Southern Medical University, Guangzhou 510515, China; luowen56286@163.com (W.L.); liyong554@zidd.ac.cn (Y.L.); panjiamin987@zidd.ac.cn (J.P.); 2 Zhongshan Institute for Drug Discovery, Shanghai Institute of Materia Medica, Chinese Academy of Sciences, Zhongshan 528400, China; zhuxinyuan623@zidd.ac.cn; 3 Shanghai Institute of Materia Medica, Chinese Academy of Sciences, Shanghai 201203, China; s20-panzilu@simm.ac.cn (Z.P.); yanli@simm.ac.cn (Y.L.); zhangyd2022@shanghaitech.edu.cn (Y.Z.); jding@simm.ac.cn (J.D.); 4 University of Chinese Academy of Sciences, Beijing 100049, China; 5 School of Pharmacy, China Pharmaceutical University, Nanjing 211198, China; 6 School of Life Science and Technology, ShanghaiTech University, Shanghai 201210, China

**Keywords:** BCAT1 inhibitor, structure–activity relationship, cancer resistance, third-generation EGFR TKI, GABA, amino acid, bicyclo[3.2.1]octane

## Abstract

Branched-chain amino acid aminotransferases (BCATs), existing as the two isoforms BCAT1 and BCAT2, are responsible for the catabolism of branched-chain amino acids (BCAAs) and are highly upregulated and implicated in a diverse range of cancers. BCAT1 inhibitors represent a potential class of therapeutic agents for cancers; however, none have yet progressed to clinical development. Our earlier research identified **WQQ-345** as a novel BCAT1 inhibitor featuring a unique bridged bicyclic skeleton and demonstrating both in vitro and in vivo antitumor activity against tyrosine kinase inhibitor (TKI)-resistant lung cancer with high BCAT1 expression. In the present study, we proceeded to modify the structure of **WQQ-345** by two-round structure–activity relationship (SAR) exploration, leading to the discovery of a bicyclo[3.2.1]octene-bearing GABA derivative **7**. Compound **7** exhibited a 6-fold enhancement in BCAT1 enzymatic inhibitory activity compared to the parent compound **WQQ-345** and could effectively suppress the growth of 67R cells that highly expressed BCAT1 and was resistant to third-generation TKIs. GABA derivatives are an important chemical class of BCAT1 inhibitors, and therefore, the findings in the present study represent great progress both in the discovery of potent BCAT1 inhibitors with new chemical structures and in the treatment of cancer resistance.

## 1. Introduction

**Figure 1:**
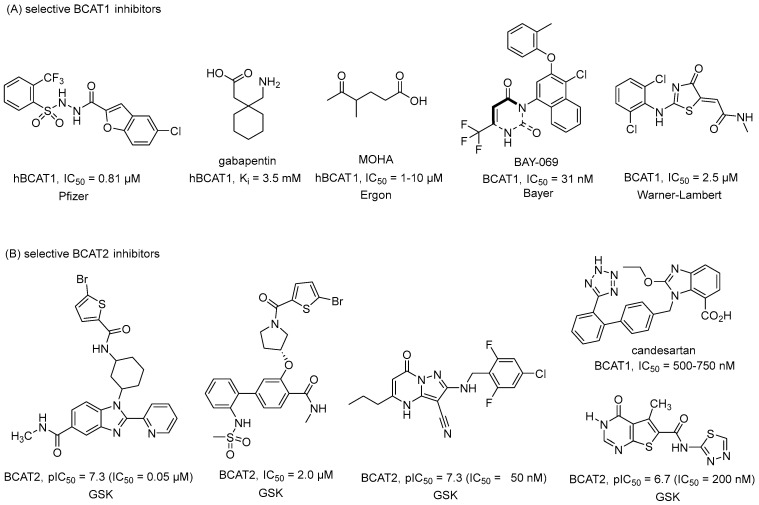
Structures of representative BCAT1 and BCAT2 inhibitors.

Branched-chain amino acids (BCAAs) (leucine (Leu), isoleucine (Ile), and valine (Val)) are essential amino acids for humans and other mammalians and play significant roles in many physiological and pathological processes [1]. The catabolism of BCAAs is catalyzed by branched-chain amino acid aminotransferase (BCAT), which exists as two isoforms, cytosolic BCAT1 (or BCATc) and mitochondrial BCAT2 (or BCATm) [2]. BCATs are highly upregulated and implicated in the occurrence, progress and metastasis of a wide range of cancers and are involved with some other diseases, such as metabolic and neurological diseases [3,4,5,6,7]. Therefore, targeting BCAT is supposed to be a potential therapeutic approach for these diseases, in particular cancers. During the past three decades, substantial efforts have been made in the discovery of BCAT1 inhibitors for the therapeutic purposes; however, none have yet advanced to clinical trials [8]. Representative selective BCAT1 and BCAT2 inhibitors are shown in Figure 1 [8]. Currently, the most advanced BCAT1 inhibitor is BAY-069, which exhibited very potent enzymatic inhibitory activity against BCAT1 as well as a decent in vitro and in vivo pharmacokinetic profile, but, unfortunately, it displayed weaker cellular inhibitory activity against BCAT1 and almost no anti-proliferative activity due to unknown reasons [9]. Therefore, further efforts are greatly needed to discover BCAT inhibitors with a more druggable profile to validate the therapeutic value in proof-of-concept clinical trials.

**Figure 2:**
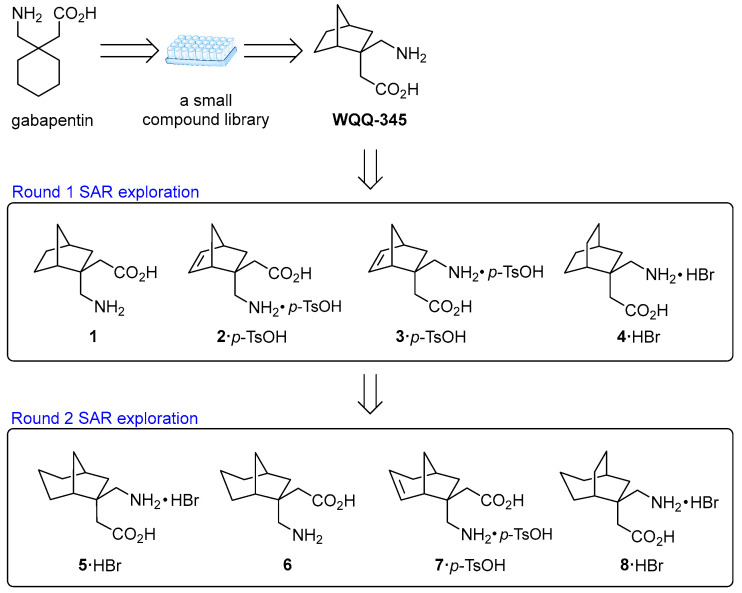
The design strategy of GABA derivatives containing bridged bicyclic skeleton as BCAT1 inhibitors.

Resistance to cancer therapies has been a long-standing challenge in cancer treatment [10], and resistance to epidermal growth factor receptor tyrosine kinase inhibitors (EGFR TKIs) has been the primary cause of cancer recurrence and treatment failure in patients with EGFR-mutant non-small-cell lung cancer (NSCLC) [11]. Our earlier research identified BCAT1 as a potential therapeutic target via high-throughput proteomics followed by genetic depletion and pharmacological inhibition [12], and furthermore, a novel BCAT1 inhibitor with a unique bridged bicyclic skeleton, namely **WQQ-345** (Figure 2), was identified by the screening of an in-house compound library of γ-aminobutyric acid (GABA) derivatives. **WQQ-345** was further demonstrated to exhibit both in vitro and in vivo antitumor activity against TKI-resistant lung cancer with high BCAT1 expression [12]. Based on the earlier promising findings, we proceeded to modify the structure of **WQQ-345** with the aim of discovering more potent BCAT1 inhibitors. Thus, in the present study, two rounds of structure–activity relationship (SAR) explorations around **WQQ-345** were conducted and a bicyclo[3.2.1]octene-bearing GABA derivative **7** was discovered (Figure 2), which was markedly more potent than the parent compound **WQQ-345** as indicated by the 6-fold improvement of BCAT1 enzymatic inhibitory activity (IC_50_, 0.78 mM for **7** versus 4.87 mM for **WQQ-345**) and could effectively suppress the growth of ASK 120067-resistent cells with high BCAT1 expression.

## 2. Results and Discussion

### 2.1. Chemistry

**Scheme 1:**
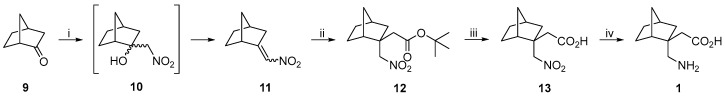
Synthetic route to compound **1**. *Reagents and conditions*: (i) CH_3_NO_2_, piperidine, PhH, reflux, 58%; (ii) (a) LDA, CH_3_CO_2_Bu*^t^*, dried THF, −78 °C, N_2_, (b) add **11** to the resulting mixture in (a), −78 °C-rt, N_2_, 58%; (iii) TFA, CH_2_Cl_2_, 0 °C-rt, 76%; (iv) H_2_ (balloon), Pd(OH)_2_, MeOH, rt, 24%.

The synthetic route to target compound **1** was shown in Scheme 1. The Henry reaction of norcamphor (**9**) and CH_3_NO_2_ in refluxing benzene gave a tertiary β-nitro alcohol **10**, which underwent spontaneous dehydration in situ to afford α,β-unsaturated nitro compound **11** as a mixture of *E*/*Z* geometric isomers [13]. The Michael addition of carbanion generated from deprotonation of *tert*-butyl acetate by lithium diisopropylamide (LDA) to **11** gave **12**. Cleavage of *tert*-butyl group in **12** by trifluoroacetic acid (TFA) in CH_2_Cl_2_ at room temperature afforded **13**, which was subjected to standard Pd(OH)_2_-catalyzed hydrogenolysis to reduce the nitro group to produce the desired target compound **1**.

**Scheme 2:**
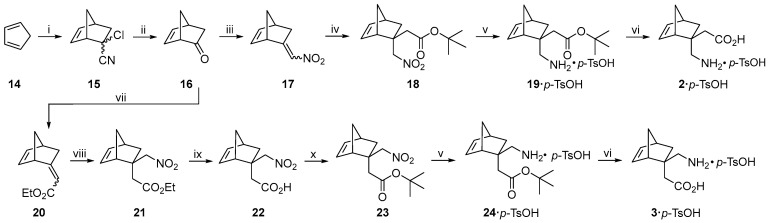
Synthetic route to compound **2** *p*-tosylate and **3** *p*-tosylate. *Reagents and conditions*: (i) CH_2_=C(Cl)CN, PhH, reflux, 36 h; (ii) KOH, EtOH, reflux, 23% (overall for **14** to **16**); (iii) CH_3_NO_2_, piperidine, PhH, reflux, 69%; (iv) (a) LDA, CH_3_CO_2_Bu*^t^*, dried THF, −78 °C, N_2_, (b) add **17** to the resulting mixture in (a), −78 °C-rt, 74%; (v) (a) Fe/NH_4_Cl, EtOH/H_2_O, reflux, N_2_; (b) *p*-TsOH·H_2_O, EtOAc, rt, 19% (for **18** to **19**·*p*-TsOH) and 37% (for **23** to **24**·*p*-TsOH); (vi) (a) aqNaHCO_3_/EtOAc; (b) TFA, CH_2_Cl_2_, rt; (c) *p*-TsOH·H_2_O, EtOAc, rt, 63% (for **19**·*p*-TsOH to **2**·*p*-TsOH) and 58% (for **24**·*p*-TsOH to **3**·*p*-TsOH); (vii) (EtO)_2_P(=O)CH_2_CO_2_Et/*t*-BuOK, dried THF, 0 °C, N_2_, 72%; (viii) CH_3_NO_2_, DBU, 85 °C, 31%; (ix) aqLiOH, EtOH, rt, 95%; (x) CH_2_=C(CH_3_)_2_, concentrated H_2_SO_4_, −15 °C-rt, 31%.

The synthetic routes to target compounds **2** and **3** are shown in Scheme 2. Diels–Alder cycloaddition of freshly distilled 1,3-cyclopentadiene (**14**) [14] and 2-chloroacrylonitrile in refluxing benzene afforded α-chloronitrile **15** [15], which was subsequently hydrolyzed with KOH in refluxing ethanol to give ketone **16**. Compound **16** was converted to **18** by a method identical to that for the synthesis of **12** from **9**. The nitro group in **18** was reduced to the corresponding amino group by iron powder in the presence of NH_4_Cl in refluxing ethanol to afford **19**, which was then converted to its crystalline *p*-toluenesulfonic acid (*p*-tosylic acid, *p*-TsOH) salt in EtOAc for purification via crystallization. Free **19** was liberated from its *p*-TsOH salt by treatment with aqueous NaHCO_3_, and its *tert*-butyl group was subsequently cleaved with TFA at room temperature in CH_2_Cl_2_ to give **2** as TFA salt, which was transformed to its crystalline *p*-tosylic acid salt, **2**•*p*-TsOH, by treatment with *p*-TsOH•H_2_O in EtOAc at room temperature. On the other hand, Witting reaction of ketone **16** and triethyl phosphonoacetate in dried THF at 0 °C afforded α,β-unsaturated ester **20** as a mixture of *E*/*Z* geometric isomers, which in turn underwent Michael addition with CH_3_NO_2_ to give **21**. The ethyl ester **21** was hydrolyzed to corresponding acid **22** with LiOH in EtOH at room temperature, and the latter was converted to the corresponding *tert*-butyl ester **23** by treatment with isobutene in the presence of concentrated H_2_SO_4_ as catalyst at −15 °C to 0 °C [16]. Following the procedure for the synthesis of **2**•*p*-TsOH from **18**, **23** was converted to the target compound **3**•*p*-TsOH. It should be noted that compounds **18**, **19** and **2** are epimers at position 3 of GABA backbone of **23**, **24** and **3**, respectively.

**Scheme 3:**
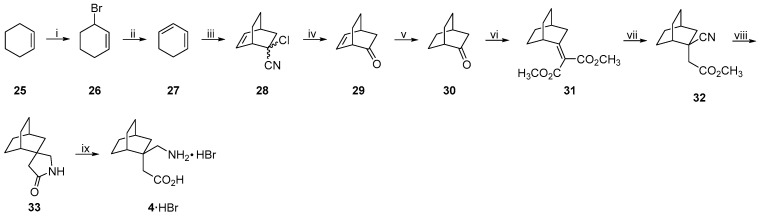
Synthetic route to compound **4** hydrobromide. *Reagents and conditions*: (i) NBS/AIBN, CCl_4_, reflux; (ii) Quinoline, reflux, 42% (overall for **25** to **27**); (iii) CH_2_=C(Cl)CN, hydroquinone, PhCH_3_, 80 °C (autoclave), N_2_; (iv) KOH, EtOH, reflux, 12% (overall for **27** to **29**); (v) H_2_, Pd/C, MeOH, rt, 80%; (vi) CH_2_(CO_2_CH_3_)_2_, TiCl_4_/pyridine, dried THF/CH_2_Cl_2_, −15 °C-rt, N_2_, 60%; (vii) TMSCN/K_2_CO_3_, MeOH, reflux, N_2_, 50%; (viii) NiCl_2_•6H_2_O/NaBH_4_, MeOH, 0 °C-rt, N_2_, 62%; (ix) 40% HBr, reflux, N_2_, 24%.

The synthetic route to **4** is shown in Scheme 3, part of which has been reported in an earlier work [17]. Thus, free-radical bromination of cyclohexene (**25**) with *N*-bromosuccinimide (NBS) in the presence of 2,2′-azodiisobutyronitrile (AIBN) as a catalyst in refluxing CCl_4_ afforded 3-bromocyclohexene (**26**). Base-mediated elimination of HBr from **26** was achieved by heating a mixture of **26** and quinoline at reflux and the produced low-boiling-point 1,3-cyclohexadiene (**27**) was distilled out from the reaction mixture and purified by fractional distillation. **27** was converted to target compound **4** as hydrobromic acid salt following a procedure reported earlier by our laboratory.

**Scheme 4:**
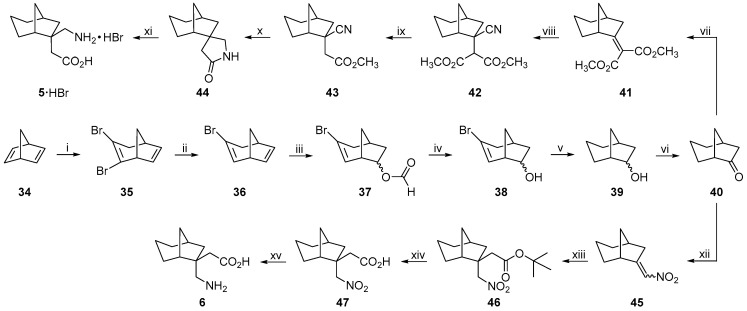
Synthetic route to compound **5** hydrobromide and **6**. *Reagents and conditions*: (i) CHBr_3_/*t*-BuOK, dried *n*-hexane, −35 °C, N_2_; (ii) LiAlH_4_, dried THF, rt, N_2_, 58% (overall for **34** to **36**); (iii) HCO_2_H, rt, 36 h; (iv) NaOH, EtOH/H_2_O, 0 °C-rt, 77% (overall for **36** to **38**); (v) H_2_ (1.5 MPa), 10% Pd/C, Et_3_N, MeOH, rt, 86%; (vi) CrO_3_/concentrated H_2_SO_4_, acetone/H_2_O, 0 °C, 56%; (vii) CH_2_(CO_2_CH_3_)_2_, TiCl_4_/pyridine, dried THF/CH_2_Cl_2_, −15 °C-rt, N_2_; (viii) TMSCN/K_2_CO_3_, MeOH, reflux, N_2_, 27% (overall for **40** to **42**); (ix) NaCl/H_2_O, DMF, reflux, 50%; (x) NiCl_2_•6H_2_O/NaBH_4_, MeOH, 0 °C-rt, N_2_, 58%; (xi) 40% HBr, reflux, N_2_, 44%; (xii) CH_3_NO_2_, piperidine, PhH, reflux, 30%; (xiii) (a) LDA, CH_3_CO_2_Bu*^t^*, dried THF, −78 °C, N_2_, (b) add **45** to the resulting mixture in (a), −78 °C-rt, 59%; (xiv) TFA, CH_2_Cl_2_, rt, 74%; (xv) H_2_, Pd(OH)_2_, MeOH, rt, 14%.

The synthetic routes to **5** and **6** are shown in Scheme 4. Treatment of norbornadiene (**34**) with CHBr_3_ in the presence of *t*-BuOK in dried *n*-hexane at −35 °C to room temperature produced dibromoolefin **35** via ring expansion involving dibromocarbene generated in situ, which was subsequently reduced by LiAlH_4_ in dried THF at room temperature to give monobromoolefin **36** [18]. The addition of HCO_2_H to one C=C double bond that was not substituted by a Br atom led to the formation of formate **37**, which was in turn hydrolyzed with NaOH in aqueous EtOH to afford alcohol **38** at room temperature. Concomitant reductions of the C-Br bond and C=C double bond in **38** to **39** were achieved with hydrogenation over 10% Pd/C in the presence of Et_3_N at high pressure (1.5 MPa) at room temperature. Oxidation of the secondary alcohol **39** with Jones reagent [19] in acetone at 0 °C afforded ketone **40**. Ketone **40** was in turn subjected to TiCl_4_/pyridine-mediated Knoevenagel condensation with dimethyl malonate to afford α,β-unsaturated malonate **41**. **41** underwent Michael addition with TMSCN in the presence of K_2_CO_3_ in refluxing MeOH to give adduct **42**, which was then subjected to decarboxylation in the presence of NaCl and H_2_O in refluxing DMF to give **43**. NiCl_2_-mediated reduction of the cyano group in **43** with NaBH_4_ gave corresponding amino group, which attacked the intramolecular ester functionality to give lactam **44**. The configuration of **44** was confirmed by single-crystal X-ray diffraction (Figure S2, Table S1). Hydrolytic ring-opening of the lactam moiety in **44** was achieved in refluxing 40% hydrobromic acid to afford target compound **5** as HBr salt. Following the procedure for the synthesis of **1** from **9**, ketone **40** was converted to target compound **6**. Like the interrelationship of compounds **2** with respect to **3**, compounds **5** and **6** are epimers with respect to each other at position 3 of GABA backbone.

**Scheme 5:**
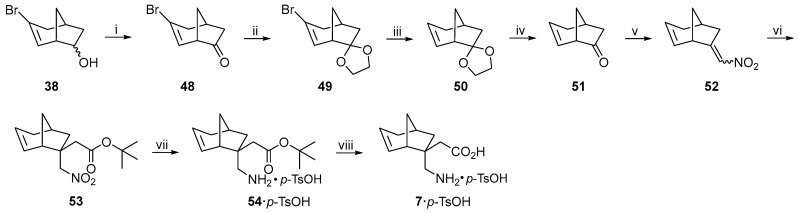
Synthetic route to compound **7** *p*-tosylate. *Reagents and conditions*: (i) CrO_3_/concentrated H_2_SO_4_, acetone/H_2_O, 0 °C, 27%; (ii) (CH_2_OH)_2_, *p*-TsOH•H_2_O, PhCH_3_, reflux, 84%; (iii) *n*-BuLi, dried THF, −78 °C, N_2_, and then H_2_O, 56%; (iv) *p*-TsOH, acetone/H_2_O, reflux, 57%; (v) CH_3_NO_2_, piperidine, PhH, 50%; (vi) (a) LDA, CH_3_CO_2_Bu*^t^*, dried THF, −78 °C, N_2_, (b) add **52** to the resulting mixture in (a), −78 °C-rt, 37%; (vii) (a) Fe/NH_4_Cl, EtOH/H_2_O, reflux, N_2_; (b) *p*-TsOH•H_2_O, EtOAc, rt, 24%; (viii) (a) aqNaHCO_3_/EtOAc, rt; (b) TFA, CH_2_Cl_2_, rt; (c) *p*-TsOH•H_2_O, EtOAc, rt, 61%.

The synthetic route to target compound **7** was shown in Scheme 5. Thus, alcohol **38** was oxidized to ketone **48** with Jones reagent in acetone at 0 °C. Reductive removal of the Br atom in **48** to give corresponding **51** was achieved by a sequential steps involving protection of carbonyl group to cyclic ketel **49** with ethylene glycol in the presence of *p*-TsOH as catalyst in refluxing benzene with azeotropic removal of water, Br-Li exchange by treatment of **49** with *n*-BiLi followed by quenching with water to give **50**, and deprotection of cyclic ketel in **50** to **51** with *p*-TsOH in refluxing acetone/water. Following the procedure for the synthesis of **2**•*p*-TsOH from **16**, ketone **51** was converted to target compound **7**•*p*-TsOH.

**Scheme 6:**
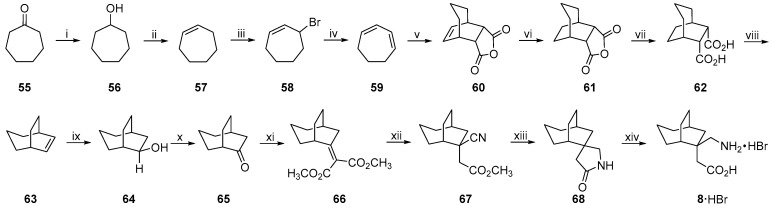
Synthetic route to compound **8** hydrobromide. *Reagents and conditions*: (i) NaBH_4_, MeOH/H_2_O, 0 °C-rt, 92%; (ii) *p*-TsOH•H_2_O, 160 °C, 100%; (iii) NBS/AIBN, CCl_4_, reflux; (iv) Quinoline, 165 °C, followed by fractional distillation to collect **59**; (v) Maleic anhydride, PhCH_3_, reflux, N_2_, 33% (overall for **57** to **60**); (vi) H_2_ (balloon), 10% Pd/C, MeOH, rt, 99%; (vii) (a) aqNaOH, rt; (b) H_3_PO_4_, 0 °C-rt, 84%; (viii) Pb(OAc)_4_, pyridine, 75 °C, 100%; (ix) (a) BH_3_·Me_2_S, dried THF, 0 °C-rt, N_2_; (b) 30%H_2_O_2_, aqNaOH, EtOH, 0 °C-rt, 97%; (x) CrO_3_/concentrated H_2_SO_4_, acetone/H_2_O, 0 °C, 49%; (xi) CH_2_(CO_2_CH_3_)_2_, TiCl_4_/pyridine, dried THF/CH_2_Cl_2_, -15 °C-rt, N_2_; (xii) TMSCN/K_2_CO_3_, MeOH, reflux, 29% (overall for **65** to **67**); (xiii) NiCl_2_•6H_2_O/NaBH_4_, MeOH, 0 °C-rt, N_2_, 53%; (xiv) 40% HBr, reflux, N_2_, 11%.

The synthetic route to **8** is shown in Scheme 6. Cycloheptanone (**55**) was reduced to cycloheptanol (**56**) with NaBH_4_ in MeOH/H_2_O at 0 °C to room temperature, and the latter was dehydrated to cycloheptene (**57**) by heating at 160 °C in the presence of *p*-TsOH as catalyst [20]. Cycloheptene (**57**) was converted to 1,3-cycloheptadiene (**59**) by a procedure used for the synthesis of **27** from **25** described above involving α-bromination of the C=C double bond and subsequent base-mediated elimination of HBr to form the second C=C double bond. Diel–Alder cycloaddition of **59** and maleic anhydride in refluxing toluene gave **60** [21]. The C=C double bond in **60** was saturated by hydrogenation over Pd/C at atmospheric pressure at room temperature to give **61**, which was then hydrolyzed with NaOH to biscarboxylic acid **62**. Pb(OAc)_4_-mediated bisdecarboxylation of **62** in pyridine at 75 °C led to formation of olefin **63** [22]. Hydroboration-oxidation of **63** with BH_3_•Me_2_S and subsequent H_2_O_2_ gave alcohol **64** [23], which was oxidized with Jones reagent in acetone at 0 °C to give ketone **65**. Following the procedure for the synthesis of **5**•HBr from **40**, **65** was converted to target compound **8** as HBr salt. Like the conversion of **41** to **43**, the conversion of **66** to **67** included two steps, the first one being Michael addition and the second one being decarboxylation at elevated temperature; however, unlike the decarboxylation for **42** to give **43** that needed very high temperature (refluxing DMF), the decarboxylation of the adduct produced from addition of TMSCN **66** occurred at comparatively low temperature (refluxing MeOH). The configuration of **68** was also confirmed by single-crystal X-ray diffraction (Figure S3, Table S1).

The sample of **WQQ-345** used in the present study was its HBr salt instead of its original free base [12], because the former had a more favorable physicochemical property and could be synthesized by a more convenient procedure as shown in supporting information (Scheme S1). The configuration of the key intermediate **S4** was confirmed by single-crystal X-ray diffraction (Figure S1, Table S1).

### 2.2. BCAT1 Enzyme Inhibition

The inhibitory activity of the synthesized compounds against BCAT1 enzyme was screened by a known method, where leucine dehydrogenase (LeuDH) was utilized to catalyze the nicotinamide adenine dinucleotide (NADH)-dependent reduction of the BCAA α-ketoisocaproate (α-KIC) to leucine and NADH consumption was measured through fluorescence and luminescence readouts to assess BCAT1 activity [9]. The half-maximal inhibitory concentration (IC_50_) values of all compounds are listed in Table 1.

Thus, **WQQ-345** and gabapentin showed robust dose-dependent BCAT1 inhibition, with IC_50_ values of 4.86 mM and 5.07 mM, respectively, which were consistent with those reported in [12,24,25]. In the first-round SAR exploration, four compounds were designed based on the structure of **WQQ-345**, i.e., compounds **1**–**4**. These new structures represent a variety of structural variations, such as chiral inversion at position 3 of the GABA backbone (**1**), the incorporation of a C=C double bond in the bridged bicycle (**3**), the combination of chiral inversion and the C=C double bond (**2**), and ring expansion of the bridged bicycle (**4**). Examination of the IC_50_ values of **1**–**4** revealed that chiral inversion at position 3 of the GABA backbone (**1** vs. **WQQ-345**) or the incorporation of the C=C double bond in the bridged bicycle (**3** vs. **WQQ-345**) slightly increased the BCAT1 inhibitory activity, while a combination of these two variations were ineffective in further improving the activity (**2** vs. **WQQ-345**). Ring expansion of the bridged bicycle (**4** vs. **WQQ-345**) resulted in the most potent activity in this round, indicating that it was likely that the bulkier bridged bicycle further enhanced the BCAT1 inhibitory activity, which inspired us to design new second-round structures, i.e., compounds **5**–**8**, by further increasing the size of the bridged bicycle. Examination of compounds **5**–**8** revealed that **7** had the most potent activity in the second round and exhibited improved activity compared to **4**; however, **8,** with the biggest bridged bicycle, was the least potent compound, indicating that the trend of increasing activity as the size of the bridged bicycle increases, as observed in the first round, stopped at compound **7**. Another SAR rule should be noted that when the aminomethyl moiety adapted the *endo* orientation with respect to the bridged bicycle, the activity was always better than the corresponding epimer, as demonstrated by the comparison of **WQQ-345** vs. **1**, **2** vs. **3**, and **5** vs. **6**.

### 2.3. Effect on Inhibition of BCAT1-Driven Cell Proliferation In Vitro

**Figure 3:**
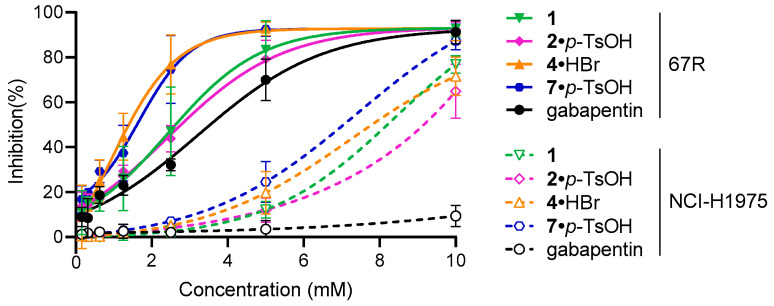
Dose–inhibition curves of proliferation inhibition of BCAT1 inhibitors **1**, **2**, **4**, **7** and gabapentin in 67R cells and NCI-H1975 cells for 120 h (n = 3; data are expressed as mean ± SD).

The third-generation EGFR tyrosine kinase inhibitor, ASK120067, has been shown to selectively inhibit the proliferation of NCI-H1975 cells by targeting the EGFR T790M mutation [26]. In our earlier study, we demonstrated that BCAT1 was highly expressed in ASK120067-resistant cells (designated as 67R) compared to parental NCI-H1975 cells, where it contributed to cell survival and conferred resistance to ASK120067 [12], and BCAT1 inhibitors could effectively suppress the growth of 67R cells [12]. Subsequently, we evaluated the in vitro anti-proliferative activity of compounds **1**, **2**, **4** and **7** on the proliferation of both 67R and NCI-H1975 cells, using gabapentin as positive controls (Figure 3 and Table 2). These four compounds represented the potent BCAT1 inhibitors in the above enzymatic assay. Notably, all of these BCAT1 inhibitors displayed potent inhibition specifically against 67R cells compared to NCI-H1975 cells. Among tested compounds, **2** and **7** showed the most significant inhibitory effects on 67R cells, with IC_50_ values below 2 mM, which was in good agreement with the trend observed in enzymatic assays described above.

As shown in Figure 1, GABA derivatives, as exemplified by gabapentin, are an important class of chemical structures of BCAT1 inhibitors, and therefore, the findings in the present study represent great progress both in the discovery of potent BCAT1 inhibitors with new chemical structures and in the treatment of cancer resistance to current third-generation EGFR tyrosine kinase inhibitors.

## 3. Materials and Methods

### 3.1. Chemistry

Unless stated otherwise, all the chemicals were obtained commercially (Bide Pharmatech Co., Ltd., Shanghai, China) and used without further purification. All the dried solvents were prepared by standard methods. Reaction progress was monitored by thin-layer chromatography (TLC) on commercially available precoated TLC silica gel plate (Xinnuo Chemical Co., Ltd., Yantai, China). Purifications on flash column chromatography were performed on silica gel column (200–300 mesh, Xinnuo Chemical Co., Ltd., Yantai, China) eluting with EtOAc/*n*-hexane (ratio was expressed as *v*/*v*). Melting points were measured in open capillaries with an SGW X-4A microscopic melting point apparatus (Shanghai INESA Physico-Optical Instrument Co., Ltd., Shanghai, China) and are uncorrected. All ^1^H and ^13^C NMR spectra were recorded on a Bruker Ascend 500 spectrometer (Bruker Switzerland AG, Fällanden, Switzerland) with CDCl_3_, DMSO-d_6_, CD_3_OD or D_2_O as solvent and TMS (for ^1^H NMR) or known chemical shifts of carbon signals of deuterated solvents (for ^13^C NMR) as internal standard. High-resolution mass spectra (HR-MS) were determined with a Thermo Scientific Exactive Plus mass spectrometer (Thermo Fisher Scientific, Bremen, Germany) using electrospray ionization (ESI) and Orbitrap techniques. Hydrogenolysis/hydrogenation requiring pressures higher than atmospheric pressure were conducted on an AE250 hydrogenator (Labe Instrument Co., Ltd., Shanghai, China) equipped with a TH-5300H high-pressure hydrogen generator (Oushisheng Technology, Co., Ltd., Beijing, China), and the reaction temperature and H_2_ pressure could be controlled automatically and continuously. Single-crystal X-ray diffractions were performed on a Bruker APEX-II CCD four-circle diffractometer (Bruker AXS Inc., Madison, WI, USA) equipped with graphite-monochromatic Mo Kα radiation (λ = 0.71073 Å) at 150.00/100.00 K. Reflections were collected by using a φ-ω scan mode. Data reduction and cell refinement were carried out using the SAINT V8.40B (Bruker, 2016) or SAINT 8.38A (Bruker, 2018) software. The structures were solved by the direct method and refined with SHELXL 2018 [27]. The crystallographic data have been deposited at Cambridge Crystallographic Data Centre (CCDC).

(*E*/*Z*)-(1R*,4S*)-2-(Nitromethylene)bicyclo[2.2.1]heptane (**11**). To a stirred solution of compound **9** (5.00 g, 45.4 mmol) in benzene (60 mL) were added CH_3_NO_2_ (30 mL) and piperidine (1.90 g, 22.3 mmol) successively. The reaction mixture was refluxed for 3 h, when TLC analysis indicated the completion of the reaction. On cooling to room temperature, the reaction mixture was poured into ice water (150 mL), and the obtained mixture was adjusted to pH 6 with 1 M HCl. The resulting mixture was extracted with CH_2_Cl_2_ (30 mL × 2), and the combined extracts were washed successively with aqueous NaHCO_3_ (30 mL × 2) and brine (30 mL), dried over anhydrous MgSO_4_, and evaporated on a rotary evaporator (Heqi Glass Co., Ltd., Shanghai, China) to afford a residue, which was purified by column chromatography (silica gel, EtOAc/*n*-hexane = 3/97) to give **11**. Pale-yellow oil, 4.01 g (58%)). Compound **11** was a mixture of *E*/*Z* geometric isomers and was used directly in the next step without further purification and characterization.

*tert*-Butyl 2-((1R*,2R*,4S*)-2-(nitromethyl)bicyclo[2.2.1]heptan-2-yl)acetate (**12**). To a stirred solution of diisopropylamine (3.96 g, 39.1 mmol) in dried THF (20 mL) cooled at −78 °C under N_2_, *n*-BuLi (1.6 M solution in THF, 24.5 mL, 39.2 mmol) was added dropwise. After addition, the mixture thus obtained was stirred for 0.5 h at this temperature. A solution of CH_3_CO_2_Bu*^t^* (4.55 g, 39.2 mmol) in dried THF (5 mL) was added dropwise to the reaction mixture, and after addition, the resulting mixture was stirred for another 0.5 h at this temperature. A solution of compound **11** (2.00 g, 13.1 mmol) in dried THF (5 mL) was added dropwise to the above mixture. After addition, the reaction mixture was stirred at −78 °C for 2 h, after which TLC analysis indicated the completion of the reaction. The reaction mixture was poured into ice water (150 mL) and the obtained mixture was adjusted to pH 6 with 1 M HCl. The resulting mixture was extracted with CH_2_Cl_2_ (30 mL × 3) and the combined extracts were washed successively with aqueous NaHCO_3_ (30 mL) and brine (30 mL), dried over anhydrous MgSO_4_ and evaporated on a rotary evaporator to afford a residue, which was purified by column chromatography (silica gel, EtOAc/*n*-hexane = 3/97) to give **12**. Colorless oil, 2.05 g (58%). ^1^H NMR (500 MHz, CDCl_3_) *δ* 4.93 (d, *J* = 12.5 Hz, 1H), 4.54 (dd, *J* = 12.8 Hz and 1.2 Hz, 1H), 2.57 (d, *J* = 16.5 Hz, 1H), 2.36 (dd, *J* = 16.8 Hz and 1.2 Hz, 1H), 2.26–2.29 (m, 2H), 1.56–1.70 (m, 4H), 1.48–1.53 (m, 1H), 1.45 (s, 9H), 1.30–1.32 (m, 1H), 1.16 (dd, *J* = 13.2 Hz and 2.8 Hz, 1H), 1.08–1.14 (m, 1H); ^13^C NMR (126 MHz, CDCl_3_) *δ* 170.97, 81.13, 79.22, 44.74, 43.27, 42.72, 41.72, 37.66, 37.24, 28.25, 28.13, 24.61; ESI-HR-MS: (*m*/*z*) calcd. for C_14_H_23_NO_4_Na ([M+Na]^+^) 292.1519, found: 292.1519.

2-((1R*,2R*,4S*)-2-(Nitromethyl)bicyclo[2.2.1]heptan-2-yl)acetic acid (**13**). To a stirred solution of compound **12** (2.00 g, 7.43 mmol) in CH_2_Cl_2_ (12 mL) cooled at 0 °C was added dropwise TFA (12 mL). The reaction mixture was stirred for 3–4 h at room temperature, after which TLC analysis indicated the completion of the reaction. The reaction mixture was evaporated on a rotary evaporator to afford a white solid, which was triturated with *n*-hexane (10 mL) at room temperature to give **13**. White solid, 1.20 g (76%). m.p. 132.4 °C–138.5 °C. ^1^H NMR (500 MHz, CDCl_3_) *δ* 4.96 (d, *J* = 12.5 Hz, 1H), 4.57 (dd, *J* = 12.8 Hz and 1.2 Hz, 1H), 2.76 (d, *J* = 17.5 Hz, 1H), 2.52 (dd, *J* = 17.5 Hz and 1.0 Hz, 1H), 2.27–2.31 (m, 2H), 1.48–1.72 (m, 5H), 1.32–1.35 (m, 1H), 1.20 (dd, *J* = 13.2 Hz and 2.8 Hz, 1H), 1.10–1.15 (m, 1H); ^13^C NMR (126 MHz, CDCl_3_) *δ* 175.69, 79.12, 44.70, 42.95, 42.80, 40.22, 37.70, 37.25, 28.06, 24.55; ESI-HR-MS: (*m*/*z*) calcd. for C_10_H_15_NO_4_Na ([M+Na]^+^) 236.0893, found: 236.0883.

2-((1R*,2R*,4S*)-2-(Aminomethyl)bicyclo[2.2.1]heptan-2-yl)acetic acid (**1**). A mixture of compound **13** (1.20 g, 5.63 mmol) and Pd(OH)_2_ (0.12 g) in MeOH (15 mL) was subjected to a procedure of standard hydrogenolysis at atmospheric pressure (balloon) at room temperature overnight, when TLC analysis indicated the completion of the reaction. The reaction mixture was filtered off through celite, and the filtrate was evaporated on a rotary evaporator to afford a solid, which was triturated with a mixed solvent (3 mL, MeOH/EtOAc = 1/3) to give **1**. White solid, 0.25 g (24%). m.p. 158.9 °C–162.7 °C. ^1^H NMR (500 MHz, CD_3_OD) *δ* 3.03 (d, *J* = 13.0 Hz, 1H), 2.98 (d, *J* = 13.0 Hz, 1H), 2.50 (d, *J* = 15.0 Hz, 1H), 2.45 (d, *J* = 15.5 Hz, 1H), 2.25–2.28 (m, 2H), 1.79–1.83 (m, 1H), 1.44–1.64 (m, 4H), 1.30–1.33 (m, 1H), 1.14–1.19 (m, 1H), 1.08 (dd, *J* = 13.0 Hz and 2.5 Hz, 1H); ^13^C NMR (126 MHz, D_2_O) *δ* 180.68, 47.84, 46.75, 43.16, 42.75, 41.42, 37.30, 36.97, 27.67, 23.69; ESI-HR-MS: (*m*/*z*) calcd. for C_10_H_18_NO_2_ ([M+H]^+^) 184.1332, found: 184.1333.

(1S*,4S*)-2-Chlorobicyclo[2.2.1]hept-5-ene-2-carbonitrile (**15**). To a stirred solution of freshly distilled cyclopentadiene [14] (**14**, 32.90 g, 0.498 mol) in benzene (90 mL), CH_2_=C(Cl)CN (24.85 g, 0.398 mol) was added. The reaction mixture was refluxed for 36 h under N_2_, when TLC analysis indicated the completion of the reaction. On cooling to room temperature, the reaction mixture was evaporated on a rotary evaporator to give a yellow oil (crude **15**, 74.00 g), which was used directly in the next step without further purification.

(1S*,4S*)-Bicyclo[2.2.1]hept-5-en-2-one (**16**). To a stirred solution of KOH (81.20 g, 1.45 mol) in EtOH (400 mL) cooled at 0 °C, solution of crude **15** prepared above in EtOH (80 mL). After addition, the reaction mixture was refluxed for 2 h, when TLC analysis indicated the completion of the reaction. On cooling to room temperature, the reaction mixture was evaporated on a rotary evaporator to afford a residue, which was poured into ice water (500 mL), and then the obtained mixture was adjusted to pH 6 with 10% H_3_PO_4_. The resulting mixture was extracted with CH_2_Cl_2_ (100 mL × 3), and the combined extracts were washed with brine (100 mL), dried over anhydrous MgSO_4_ and evaporated on a rotary evaporator to afford a residue, which was purified by column chromatography (silica gel, EtOAc/*n*-hexane = 1/10) to give **16**. Waxy solid, 12.60 g (overall 23% for **14** to **16**). ^1^H NMR (500 MHz, CDCl_3_) *δ* 6.57 (dd, *J* = 5.5 Hz and 3.0 Hz, 1H), 6.10–6.12 (m, 1H), 3.18–3.19 (m, 1H), 3.01–3.02 (m, 1H), 2.18–2.21 (m, 1H), 1.94–2.00 (m, 2H), 1.85 (dd, *J* = 16.5 Hz and 4.5 Hz, 1H). The ^1^H NMR data were consistent with those reported in [28].

(*E*/*Z*)-(1S*,4S*)-5-(Nitromethylene)bicyclo[2.2.1]hept-2-ene (**17**). Following the procedure for the synthesis of **11** from **9**, **17** was prepared from **16** (3.00 g, 27.7 mmol) with CH_3_NO_2_ (18 mL) and piperidine (1.18 g, 13.9 mmol) in benzene (30 mL) at reflux. **17** was isolated and purified by column chromatography (silica gel, EtOAc/*n*-hexane = 3/97). Pale-yellow oil, 2.90 g (69%). Compound **17** was a mixture of *E*/*Z* geometric isomers and was used directly in the next step without further purification.

*tert*-Butyl 2-((1S*,2R*,4S*)-2-(nitromethyl)bicyclo[2.2.1]hept-5-en-2-yl)acetate (**18**). Following the procedure for the synthesis of compound **12** from compound **11**, compound **18** was prepared from compound **17** (2.90 g, 19.2 mmol) with diisopropylamine (5.80 g, 57.3 mmol), *n*-BuLi (1.6 M solution in THF, 35.9 mL, 57.44 mmol) and CH_3_CO_2_Bu*^t^* (6.70 g, 57.7 mmol) in dried THF (40 mL). Colorless oil, 3.80 g (74%). ^1^H NMR (500 MHz, CDCl_3_) *δ* 6.20–6.25 (m, 2H), 4.71 (d, *J* = 12.0 Hz, 1H), 4.34 (d, *J* = 11.8 Hz, 1H), 2.89 (s, 1H), 2.82 (s, 1H), 2.67 (d, *J* = 17.0 Hz, 1H), 2.55 (d, *J* = 16.8 Hz, 1H), 1.77 (dd, *J* = 13.0 Hz and 3.5 Hz, 1H), 1.56–1.60 (m, 2H), 1.47 (s, 9H), 1.07 (dd, *J* = 13.0 Hz and 2.5 Hz, 1H); ^13^C NMR (126 MHz, CDCl_3_) *δ* 171.14, 137.91, 134.84, 81.25, 80.52, 50.01, 47.49, 44.43, 42.85, 42.08, 36.72, 28.25; ESI-HR-MS: (*m*/*z*) calcd. for C_14_H_22_NO_4_ ([M+H]^+^) 268.1543, found: 268.1546.

*tert*-Butyl 2-((1S*,2R*,4S*)-2-(aminomethyl)bicyclo[2.2.1]hept-5-en-2-yl)acetate (**19**) *p*-tosylate. To a stirred solution of compound **18** (3.80 g, 14.2 mmol) in a mixed solvent of EtOH (40 mL) and H_2_O (20 mL), iron powder (3.97 g, 71.1 mmol) and NH_4_Cl (1.52 g, 28.4 mmol) were added. The reaction mixture was refluxed for 5 h under N_2_, when TLC analysis indicated the completion of the reaction. On cooling to room temperature, the reaction mixture was filtered off through celite, the filtrate was dissolved in EtOAc (50 mL). The mixture was washed successively with aqueous NaHCO_3_ (50 mL × 2) and brine (50 mL), dried over anhydrous MgSO_4_ and evaporated on a rotary evaporator to afford a residue (2.10 g), which was then dissolved in EtOAc (5 mL), followed by addition of *p*-TsOH•H_2_O (1.85 g, 9.73 mmol). The mixture was stirred at room temperature until a large amount of crystals precipitated, which were collected by suction filtration and dried in vacuo at room temperature to give **19**•*p*-TsOH. White solid, 1.10 g (19%). m.p. 151.2 °C–153.0 °C. ^1^H NMR (500 MHz, CD_3_OD) *δ* 7.71 (d, *J* = 8.5 Hz, 2H), 7.24 (d, *J* = 8.0 Hz, 2H), 6.29 (dd, *J* = 5.5 Hz and 3.0 Hz, 1H), 6.17 (dd, *J* = 6.0 Hz and 3.0 Hz, 1H), 3.03 (d, *J* = 13.0 Hz, 1H), 2.91 (s, 1H), 2.85 (d, *J* = 13.0 Hz, 1H), 2.68 (s, 1H), 2.64 (d, *J* = 15.5 Hz, 1H), 2.60 (d, *J* = 15.5 Hz, 1H), 2.37 (s, 3H), 1.72 (dd, *J* = 12.5 Hz and 4.0 Hz, 1H), 1.69 (d, *J* = 9.0 Hz, 1H), 1.55–1.57 (m, 1H), 1.49 (s, 9H), 1.01 (dd, *J* = 12.0 Hz and 2.5 Hz, 1H); ^13^C NMR (126 MHz, DMSO-d_6_) *δ* 170.82, 145.73, 137.92, 137.59, 133.90, 128.04, 125.48, 80.55, 48.25, 47.23, 44.74, 43.04, 42.42, 40.93, 36.14, 27.80, 20.78; ESI-HR-MS: (*m*/*z*) calcd. for C_14_H_24_NO_2_ ([M+H]^+^) 238.1802, found: 238.1801.

2-((1S*,2R*,4S*)-2-(Aminomethyl)bicyclo[2.2.1]hept-5-en-2-yl)acetic acid (**2**) *p*-tosylate. A stirred solution of compound **19**•*p*-TsOH (1.10 g, 2.69 mmol) in a mixed solvent of EtOAc (10 mL) and aqueous NaHCO_3_ (20 mL) was stirred for 1 h at room temperature. After separation of the organic phase, the aqueous phase was extracted with EtOAc (10 mL × 2), and the combined extracts were washed with aqueous NaHCO_3_ (20 mL), dried over anhydrous MgSO_4_ and evaporated on a rotary evaporator to afford a residue. To a stirred solution of this residue in CH_2_Cl_2_ (3 mL) cooled at 0 °C was added dropwise TFA (2 mL), and the stirring was continued for 5 h at room temperature, when TLC analysis indicated the completion of the reaction. The reaction mixture was evaporated on a rotary evaporator to afford a residue, which was dissolved in EtOAc (3 mL) followed by addition of *p*-TsOH•H_2_O (0.51 g, 2.68 mmol). The mixture thus obtained was stirred at room temperature until a large amount of crystals precipitated. The crystals were collected via suction filtration and dried in vacuo at room temperature to give **2**•*p*-TsOH. White solid, 0.60 g (63%). m.p. 176.8 °C–178.7 °C. ^1^H NMR (500 MHz, CD_3_OD) *δ* 7.71 (d, *J* = 7.5 Hz, 2H), 7.24 (d, *J* = 8.0 Hz, 2H), 6.29–6.30 (m, 1H), 6.17–6.18 (m, 1H), 3.06 (d, *J* = 13.0 Hz, 1H), 2.87–2.92 (m, 2H), 2.71–2.74 (m, 3H), 2.37 (s, 3H), 1.70–1.75 (m, 2H), 1.56 (d, *J* = 9.0 Hz, 1H), 1.05 (dd, *J* = 12.5 Hz and 3.0 Hz, 1H); ^13^C NMR (126 MHz, D_2_O) *δ* 176.23, 142.46, 139.36, 139,12, 133.17, 129.42, 125.33, 48.61, 47.26, 46.08, 42.79, 42.70, 41.33, 36.47, 20.44; ESI-HR-MS: (*m*/*z*) calcd. for C_10_H_16_NO_2_ ([M+H]^+^) 182.1176, found: 182.1177.

Ethyl (*E*/*Z*)-2-((1S*,4S*)-bicyclo[2.2.1]hept-5-en-2-ylidene)acetate (**20**). To a stirred solution of *t*-BuOK (5.60 g, 49.9 mmol) in dried THF (50 mL) cooled at 0 °C under N_2_, triethyl phosphonoacetate (9.70 g, 43.3 mmol) was added dropwise. After addition, the resulting mixture was stirred for 40 min at this temperature. Then, a solution of compound **16** (3.60 g, 33.3 mmol) in dried THF (36 mL) was added dropwise to the above mixture. After addition, the mixture thus obtained was stirred for 3 h at this temperature, when TLC analysis indicated the completion of the reaction. The reaction mixture was poured into water (150 mL) and the obtained mixture was extracted with EtOAc (50 mL × 3). The combined extracts were washed with brine (50 mL), dried over anhydrous MgSO_4_ and evaporated on a rotary evaporator to afford a residue, which was purified by column chromatography (silica gel, EtOAc/*n*-hexane = 1/20) to give **20** as a mixture of *E*/*Z* geometric isomers. Pale-yellow oil, 4.30 g (72%). The sample was used directly in the next step without characterization.

Ethyl 2-((1S*,2S*,4S*)-2-(nitromethyl)bicyclo[2.2.1]hept-5-en-2-yl)acetate (**21**). To a stirred solution of compound **20** (3.00 g, 16.8 mmol) in CH_3_NO_2_ (30 mL) cooled at 0 °C under N_2_, 1,8-diazabicyclo[5.4.0]undec-7-ene (DBU, 8.40 g, 67.6 mmol) was added dropwise. After addition, the reaction mixture was refluxed at 85 °C overnight, after which TLC analysis indicated the completion of the reaction. On cooling to room temperature, the reaction mixture was poured into ice water (100 mL) and the obtained mixture was adjusted to pH 6 with 1 M HCl. The resulting mixture was extracted with CH_2_Cl_2_ (30 mL × 3), and the combined extracts were washed with brine (50 mL), dried over anhydrous MgSO_4_ and evaporated on a rotary evaporator to afford a residue, which was purified by column chromatography (silica gel, EtOAc/*n*-hexane = 3/97) to give **21**. Colorless oil, 1.25 g (31%). The sample of **21** was contaminated by a certain amount of an inseparable impurity and therefore used directly in the next step without collection of the structural characterization data.

2-((1S*,2S*,4S*)-2-(Nitromethyl)bicyclo[2.2.1]hept-5-en-2-yl)acetic acid (**22**). To a stirred solution of compound **21** (2.61 g, 10.9 mmol) in EtOH (25 mL) at room temperature was added dropwise a solution of LiOH•H_2_O (1.37 g, 32.7 mmol) in distilled water (5 mL). After addition, the reaction mixture was stirred for 5 h at this temperature, when TLC analysis indicated the completion of the reaction. The reaction mixture was adjusted to pH 1 with 1 M HCl, and the obtained mixture was extracted with CH_2_Cl_2_ (20 mL × 3). The combined extracts were washed with brine (20 mL), dried over anhydrous MgSO_4_ and evaporated on a rotary evaporator to afford a residue, which was purified by column chromatography (silica gel, EtOAc/*n*-hexane = 1/3) to give **22**. Pale-yellow oil, 2.18 g (95%). The sample of **22** was contaminated by a certain amount of an inseparable impurity and therefore used directly in the next step without collection of the structural characterization data.

*tert*-Butyl 2-((1S*,2S*,4S*)-2-(nitromethyl)bicyclo[2.2.1]hept-5-en-2-yl)acetate (**23**). To a stirred mixture of compound **22** (2.18 g, 10.3 mmol) and 2-methylpropene (10% in *i*-Pr_2_O, 20 mL) cooled at −15 °C under N_2_ was added dropwise concentrated H_2_SO_4_ (1 mL). After addition, the reaction mixture was stirred at room temperature for 2 days and subsequently poured into aqueous NaHCO_3_ (50 mL). The resulting mixture was extracted with EtOAc (20 mL × 3), and combined extracts were dried over anhydrous MgSO_4_ and evaporated on a rotary evaporator to give **23**. Colorless oil, 0.86 g (31%). The sample of **23** was contaminated by a certain amount of an inseparable impurity and therefore used directly in the next step without collection of the structural characterization data.

*tert*-Butyl 2-((1S*,2S*,4S*)-2-(aminomethyl)bicyclo[2.2.1]hept-5-en-2-yl)acetate (**24**) *p*-tosylate. To a stirred solution of compound **23** (1.05 g, 3.93 mmol) in a mixed solvent of EtOH (10 mL) and H_2_O (5 mL) were added iron powder (1.08 g, 19.3 mmol) and NH_4_Cl (0.42 g, 7.85 mmol). After addition, the reaction mixture was refluxed for 5 h under N_2_, when TLC analysis indicated the completion of the reaction. On cooling to room temperature, the reaction mixture was filtered off through celite, and the filtrate was extracted with EtOAc (20 mL × 3). The extract was washed with aqueous NaHCO_3_ (20 mL × 3), dried over anhydrous MgSO_4_ and evaporated on a rotary evaporator to afford a residue (0.79 g), which was dissolved in EtOAc (5 mL) followed by addition of *p*-TsOH•H_2_O (0.76 g, 4.00 mmol). The resulting mixture was stirred at room temperature until a large amount of crystals precipitated, which were collected via suction filtration and dried in vacuo at room temperature to give **24**•*p*-TsOH. White solid, 0.59 g (37%). m.p. 178.1 °C–179.3 °C. ^1^H NMR (500 MHz, DMSO-d_6_) *δ* 7.77 (bs, 2H), 7.47 (d, *J* = 8.0 Hz, 2H), 7.11 (d, *J* = 8.0 Hz, 2H), 6.24 (dd, *J* = 5.5 Hz and 3.0 Hz, 1H), 6.06 (dd, *J* = 5.5 Hz and 3.0 Hz, 1H), 3.04–3.19 (m, 2H), 2.82 (s, 1H), 2.75 (s, 1H), 2.29 (d, *J* = 16.0 Hz, 1H), 2.29 (s, 3H), 2.16 (d, *J* = 15.5 Hz, 1H), 1.63 (dd, *J* = 12.2 Hz and 3.8 Hz, 1H), 1.56 (d, *J* = 9.0 Hz, 1H), 1.41 (s, 9H), 1.34 (d, *J* = 9.0 Hz, 1H), 0.84 (dd, *J* = 12.2 Hz and 2.8 Hz, 1H); ^13^C NMR (126 MHz, DMSO-d_6_) *δ* 170.83, 145.68, 137.86, 137.66, 134.35, 128.08, 125.51, 80.40, 47.81, 46.65, 45.83, 42.97, 42.27, 40.17, 36.34, 27.74, 20.80; ESI-HR-MS: (*m*/*z*) calcd. for C_14_H_24_NO_2_ ([M+H]^+^) 238.1802, found: 238.1802.

2-((1S*,2S*,4S*)-2-(Aminomethyl)bicyclo[2.2.1]hept-5-en-2-yl)acetic acid (**3**) *p*-tosylate. Following the procedure for the synthesis of **2**•*p*-TsOH from **19**•*p*-TsOH, **3**•*p*-TsOH was prepared from **24**•*p*-TsOH (0.58 g, 1.42 mmol) with TFA (2 mL) in CH_2_Cl_2_ (3 mL) at room temperature. White solid, 0.29 g (58%). m.p. 172.8 °C–173.2 °C. ^1^H NMR (500 MHz, CD_3_OD) *δ* 7.70 (d, *J* = 8.0 Hz, 2H), 7.23 (d, *J* = 8.0 Hz, 2H), 6.26–6.28 (m, 1H), 6.12–6.13 (m, 1H), 3.31 (d, *J* = 14.0 Hz, 1H), 3.24 (d, *J* = 13.5 Hz, 1H), 2.89 (s, 1H), 2.84–2.85 (m, 1H), 2.44 (d, *J* = 17.0 Hz, 1H), 2.37 (d, *J* = 16.0 Hz, 1H), 2.37 (s, 3H), 1.67 (dd, *J* = 12.2 Hz and 3.8 Hz, 1H), 1.61 (d, *J* = 9.5 Hz, 1H), 1.48–1.51 (m, 1H), 1.02 (dd, *J* = 12.2 Hz and 2.8 Hz, 1H); ^13^C NMR (126 MHz, D_2_O) *δ* 176.51, 142.46, 139.36, 138.60, 134.00, 129.42, 125.33, 47.84, 47.22, 46.49, 42.67, 42.49, 40.53, 37.00, 20.44; ESI-HR-MS: (*m*/*z*) calcd. for C_10_H_16_NO_2_ ([M+H]^+^) 182.1176, found: 182.1176.

3-Bromocyclohex-1-ene (**26**). To a stirred solution of cyclohexene (**25**, 80.00 g, 0.974 mol) in CCl_4_ (600 mL) at room temperature, NBS (173.34 g, 0.974 mol) was added under N_2_. The resulting mixture was heated to 65 °C and AIBN (1.58 g, 9.62 mmol) was added in portions. After addition, the reaction mixture was heated slowly to 75 °C and the heating was stopped immediately once the solution began to boil to avoid violent boiling. Approximately 10 min later, the boiling subsided and the external temperature was raised to about 80 °C (gentle boiling) for 1 h, when TLC analysis indicated the completion of the reaction. On cooling to room temperature, the reaction mixture was filtered off through celite, the filtrate was evaporated on a rotary evaporator to afford a residue, which was purified by distillation under reduced pressure (*ca* 40 mmHg) to give crude **26** as a colorless oil (108.00 g). The sample of **26** was used directly in the next step without further purification and characterization.

Cyclohexa-1,3-diene (**27**). Crude **26** (108.00 g) prepared above and quinoline (200 mL) were placed into a round-bottomed flask equipped with fractional column. The reaction mixture was heated to 120 °C and the produced **27** was distilled out of the reaction mixture and subsequently purified by fractional distillation at atmospheric pressure. Colorless oil, 33.00 g (overall 42% for **25** to **27**). b.p. 78 °C–80 °C (literature value [29], 80 °C). ^1^H NMR (CDCl_3_, 500 MHz) *δ* 5.88–5.90 (m, 2H), 5.78–5.81 (m, 2H), 2.14–2.15 (m, 4H). The ^1^H NMR data were consistent with those reported in [30].

2-((1R*,2S*,4R*)-2-(aminomethyl)bicyclo[2.2.2]octan-2-yl)acetic acid (**4**) hydrobromide. The synthesis of compound **4**•HBr from compound **27** followed a procedure reported by our laboratory [17]. White solid, 0.20 g. m.p. 188.9 °C–190.5 °C. The ^1^H NMR and ^13^C NMR data were consistent with those reported in [17].

(1R*,5R*)-2,3-Dibromobicyclo[3.2.1]octa-2,6-diene (**35**). To a stirred solution of bicyclo[2.2.1]hepta-2,5-diene (**34**, 30.00 g, 0.326 mol) in dried *n*-hexane (300 mL) cooled at −35 °C was added *t*-BuOK (43.80 g, 0.390 mol) in portions, and then a solution of CHBr_3_ (98.70 g, 0.391 mol) in dried *n*-hexane (80 mL) was added dropwise at this temperature under N_2_. After addition, the reaction mixture was stirred at this temperature for 2 h, when TLC analysis indicated the completion of the reaction. The reaction mixture was poured into ice water (600 mL), and after the separation of the organic phase, the aqueous phase was extracted with EtOAc (300 mL × 3). The combined organic phases were washed with brine (200 mL), dried over anhydrous MgSO_4_ and evaporated on a rotary evaporator to give a brown oil (crude **35**, 84.10 g), which was used directly in the next step without further purification and characterization.

(1R*,5R*)-3-Bromobicyclo[3.2.1]octa-2,6-diene (**36**). To stirred dried THF (100 mL) cooled at 0 °C, LiAlH_4_ (9.60 g, 0.253 mol) and a solution of crude **35** (84.10 g, 0.319 mol) prepared above in dried THF (200 mL) were successively added. The resulting mixture was stirred at 35 °C for 24 h, at which point TLC analysis indicated the completion of the reaction. The reaction was cooled to room temperature and then cooled in an ice-water bath, and quenched by the addition of MeOH (20 mL) in a dropwise manner. The mixture thus obtained was filtered off through celite. The filtrate was adjusted to pH 7 with 1 M HCl and extracted with EtOAc (100 mL × 3). The combined extracts were washed with brine (100 mL), dried over anhydrous MgSO_4_ and evaporated on a rotary evaporator to afford a residue, which was purified by column chromatography (silica gel, *n*-hexane) to give **36**. Pale-yellow oil, 35.00 g (overall 58% for **34** to **36**). The sample was used directly in the next step without characterization.

(1S*,5S*)-3-Bromobicyclo[3.2.1]oct-3-en-6-yl formate (**37**). A solution of compound **36** (35.00 g, 0.189 mol) in HCO_2_H (230 mL) was stirred at room temperature for 2 days, when TLC analysis indicated the completion of the reaction. The reaction mixture was poured into ice water (600 mL), and the obtained mixture was adjusted to pH 7 with aqueous NaHCO_3_. The resulting mixture was extracted with EtOAc (200 mL × 3), and the combined extracts were washed with brine (200 mL), dried over anhydrous MgSO_4_ and evaporated on a rotary evaporator to give a pale-yellow oil (crude **37**, 29.10 g), which was a mixture of diastereomers (with a ratio of 25/75 as determined by ^1^H NMR) and used directly in the next step without further purification.

(1S*,5S*)-3-Bromobicyclo[3.2.1]oct-3-en-6-ol (**38**). To a stirred solution of crude **37** (29.10 g) in EtOH (300 mL) cooled at 0 °C was added dropwise a solution of NaOH (15.10 g, 0.378 mol) in water (40 mL). After addition, the reaction mixture was stirred at room temperature for 1 h, when TLC analysis indicated the completion of the reaction. The reaction mixture was evaporated on a rotary evaporator to afford a residue, which was then poured into water (150 mL). The mixture thus obtained was adjusted to pH 6 with 1 M HCl and extracted with EtOAc (100 mL × 3). The combined extracts were washed with brine (100 mL), dried over anhydrous MgSO_4_ and evaporated on a rotary evaporator to afford a residue, which was purified by column chromatography (silica gel, EtOAc/*n*-hexane = 1/10) to give **38**. Pale-yellow oil, 29.50 g (overall 77% for **36** to **38**). Compound **38** was supposed to be a diastereomeric mixture at α carbon of the OH group and was used directly in the next step without further purification and characterization.

(1*S*,5*R*)-Bicyclo[3.2.1]octan-6-ol (**39**). A mixture of compound **38** (20.00 g, 98.5 mol), Et_3_N (27.5 mL) and 10% Pd/C (1.50 g) in MeOH (320 mL) was subjected to a procedure of standard hydrogenation at 1.5 MPa at room temperature overnight, when TLC analysis indicated the completion of the reaction. The reaction mixture was filtered off through celite and the filtrate was evaporated on a rotary evaporator to afford a residue, which was dissolved in water (200 mL). The mixture thus obtained was adjusted to pH 6 with 1 M HCl and was then extracted with EtOAc (100 mL × 3). The combined extracts were washed with brine (100 mL), dried over anhydrous MgSO_4_ and evaporated on a rotary evaporator to give crude **39**. Colorless solid, 10.70 g (86%). Compound **39** was a diastereomeric mixture at α carbon of the OH group (with a ratio of 23/77 as determined by ^1^H NMR) and was used directly in the next step without further purification and characterization.

(1S*,5R*)-Bicyclo[3.2.1]octan-6-one (**40**). Preparation of Jones reagent: To a stirred distilled water (56 mL) cooled at 0 °C, CrO_3_ (17.80 g, 0.178 mol) was added in portions, and after addition, concentrated H_2_SO_4_ (16.1 mL) was added dropwise at this temperature. To a stirred solution of compound **39** (15.00 g, 0.119 mol) in acetone (80 mL) cooled at 0 °C, the freshly prepared Jones reagent described above was added dropwise. After addition, the reaction mixture was stirred at room temperature for 4 h, ta which point TLC analysis indicated the completion of the reaction. The reaction mixture was cooled in an ice-water bath and the excessive Jones reagent was quenched by slowly adding *i*-PrOH until the brown color turned green completely. The mixture was adjusted to pH 7 with aqueous NaHCO_3_ and then extracted with EtOAc (100 mL × 3). The combined extracts were washed with brine (100 mL), dried over anhydrous MgSO_4_ and evaporated on a rotary evaporator to afford a residue, which was purified by column chromatography (silica gel, EtOAc/*n*-hexane = 1/10) to give **40**. Colorless oil, 8.30 g (56%). ^1^H NMR (500 MHz, CDCl_3_) *δ* 2.59–2.60 (m, 1H), 2.33 (t, *J* = 5.5 Hz, 1H), 2.26 (dd, *J* = 18.5 Hz and 7.0 Hz, 1H), 2.07 (dd, *J* = 18.5 Hz and 3.5 Hz, 1H), 1.96–2.01 (m, 1H), 1.82–1.86 (m, 1H), 1.57–1.76 (m, 6H); ^13^C NMR (126 MHz, CDCl_3_) *δ* 222.53, 46.36, 43.76, 37.38, 32.33, 30.81, 30.68, 19.00; ESI-HR-MS: (*m*/*z*) calcd. for C_8_H_13_O ([M+H]^+^) 125.0961, found: 125.0963.

Dimethyl 2-((1S*,5R*)-bicyclo[3.2.1]octan-6-ylidene)malonate (**41**). To a stirred mixed solvent of dried THF (5 mL) and dried CH_2_Cl_2_ (20 mL) cooled at −15 °C under N_2_ were added successively TiCl_4_ (2.30 g, 12.1 mmol) and dimethyl malonate (1.60 g, 12.1 mol) both in a dropwise manner. After addition, a solution of **40** (0.50 g, 4.03 mmol) in dried CH_2_Cl_2_ (5 mL) was added dropwise to the reaction mixture, followed by dropwise addition of pyridine (1.90 g, 24.0 mmol). The reaction mixture was then stirred at room temperature overnight, when TLC analysis indicated the completion of the reaction. The reaction mixture was poured into ice water (20 mL), and after separation of the organic phase, the aqueous phase was extracted with CH_2_Cl_2_ (20 mL × 3). The combined extracts were washed with brine (50 mL), dried over anhydrous MgSO_4_ and evaporated on a rotary evaporator to afford a residue, which was purified by column chromatography (silica gel, EtOAc/*n*-hexane = 1/10) to give **41**. Colorless oil, 0.36 g. The sample of **41** contained a certain amount of inseparable unreacted **40** and therefore was not pure enough for the collection of structural characterization. It was used directly in the next step without further purification and characterization.

Dimethyl 2-((1S*,5R*,6R*)-6-cyanobicyclo[3.2.1]octan-6-yl)malonate (**42**). To a stirred solution of crude **41** (0.36 g, deemed to be 1.51 mmol) and K_2_CO_3_ (0.24 g, 1.82 mmol) in MeOH (10 mL) cooled at 0 °C under N_2_ was added dropwise TMSCN (0.67 g, 6.75 mmol). After addition, the reaction mixture was refluxed for 24 h, when TLC analysis indicated the completion of the reaction. On cooling to room temperature, the reaction mixture was poured into ice water (20 mL) and the obtained mixture was extracted with EtOAc (10 mL × 3). The combined extracts were washed with aqueous NaHCO_3_ (10 mL), dried over anhydrous MgSO_4_ and evaporated on a rotary evaporator to afford a residue, which was purified by column chromatography (silica gel, EtOAc/*n*-hexane = 1/10) to give **42**. Colorless oil, 0.29 g (overall 27% for **40** to **42**). ^1^H NMR (500 MHz, CDCl_3_) *δ* 3.84 (s, 3H), 3.82 (s, 3H), 3.79 (s, 1H), 2.61–2.64 (m, 1H), 2.52 (dd, *J* = 14.5 Hz and 7.5 Hz, 1H), 2.40–2.43 (m, 1H), 2.20–2.25 (m, 1H), 1.71 (dd, *J* = 14.5 Hz and 1.5 Hz, 1H), 1.58–1.65 (m, 3H), 1.54–1.57 (m, 2H), 1.49–1.52 (m, 1H), 1.33–1.44 (m, 1H); ^13^C NMR (126 MHz, CDCl_3_) *δ* 167.47, 167.12, 124.12, 53.52, 53.23, 53.15, 45.33, 43.79, 40.82, 38.78, 34.06, 31.51, 26.96, 18.86; ESI-HR-MS: (*m*/*z*) calcd. for C_14_H_19_NO_4_Na ([M+Na]^+^) 288.1206, found: 288.1206.

Methyl 2-((1S*,5R*,6S*)-6-cyanobicyclo[3.2.1]octan-6-yl)acetate (**43**). To a stirred solution of compound **42** (2.30 g, 8.67 mmol) in DMF (20 mL) at room temperature, NaCl (1.00 g, 17.1 mmol) and H_2_O (0.30 g, 16.6 mmol) were added. The reaction mixture was refluxed for 5 h under N_2_, after which TLC analysis indicated the completion of the reaction. On cooling to room temperature, the reaction mixture was poured into ice water (50 mL) and the obtained mixture was extracted with EtOAc (10 mL × 4). The combined extracts were washed with brine (20 mL × 3), dried over anhydrous MgSO_4_ and evaporated on a rotary evaporator to afford a residue, which was purified by column chromatography (silica gel, EtOAc/*n*-hexane = 4/96) to give **43**. Colorless oil, 0.90 g (50%). ^1^H NMR (500 MHz, CDCl_3_) *δ* 3.76 (s, 3H), 2.86 (d, *J* = 16.5 Hz, 1H), 2.80 (d, *J* = 16.5 Hz, 1H), 2.49–2.54 (m, 2H), 2.38–2.42 (m, 1H), 2.14–2.18 (m, 1H), 1.60–1.65 (m, 3H), 1.52–1.58 (m, 3H), 1.48–1.52 (m, 1H), 1.33–1.44 (m, 1H); ^13^C NMR (126 MHz, CDCl_3_) *δ* 170.63, 125.97, 52.21, 43.80, 42.25, 40.97, 39.10, 37.41, 34.33, 31.59, 27.05, 18.80; ESI-HR-MS: (*m*/*z*) calcd. for C_12_H_18_NO_2_ ([M+H]^+^) 208.1332, found: 208.1334.

(1S*,5R*,6S*)-Spiro[bicyclo[3.2.1]octane-6,3′-pyrrolidin]-5′-one (**44**). To a stirred solution of compound **43** (0.30 g, 1.45 mmol) and NiCl_2_•6H_2_O (0.80 g, 3.37 mmol) in MeOH (10 mL) cooled at 0 °C under N_2_ was added NaBH_4_ (0.60 g, 15.9 mmol) in portions. After addition, the reaction mixture was stirred at room temperature overnight, when TLC analysis indicated the completion of the reaction. The reaction mixture was filtered off through celite, and the filtrate was diluted with water (30 mL) and extracted with EtOAc (10 mL × 3). The combined extracts were washed with brine (30 mL), dried over anhydrous MgSO_4_ and evaporated on a rotary evaporator to afford a white solid, which was triturated with *n*-hexane (3 mL) at room temperature to give **44**. White solid, 0.15 g (58%). m.p. 126.1 °C–127.2 °C. ^1^H NMR (500 MHz, CDCl_3_) *δ* 5.58 (bs, 1H), 3.21 (d, *J* = 9.5 Hz, 1H), 3.17 (d, *J* = 9.0 Hz, 1H), 2.53 (d, *J* = 17.0 Hz, 1H), 2.40 (d, *J* = 16.5 Hz, 1H), 2.26 (s, 1H), 1.87–1.94 (m, 2H), 1.62–1.70 (m, 2H), 1.59 (dd, *J* = 14.0 Hz and 2.0 Hz, 1H), 1.46–1.53 (m, 4H), 1.42–1.46 (m, 2H); ^13^C NMR (126 MHz, CDCl_3_) *δ* 178.57, 58.86, 48.64, 43.71, 39.03, 38.93, 34.97, 32.36, 28.99, 18.95; ESI-HR-MS: (*m*/*z*) calcd. for C_11_H_18_NO ([M+H]^+^) 180.1383, found: 180.1384.

2-((1S*,5R*,6S*)-6-(Aminomethyl)bicyclo[3.2.1]octan-6-yl)acetic acid (**5**) hydrobromide. A solution of compound **44** (0.45 g, 2.51 mmol) in 40% HBr (10 mL) was refluxed overnight under N_2_; TLC analysis indicated the completion of the reaction. On cooling to room temperature, the reaction mixture was evaporated on a rotary evaporator to afford a residue, which was dissolved in distilled water (50 mL). The obtained mixture was extracted with EtOAc (50 mL × 3) to remove any unreacted starting material, and the aqueous layer was evaporated on a rotary evaporator to give a solid, which was triturated at room temperature with EtOAc (5 mL) to give **5**•HBr. White solid, 0.31 g (44%). m.p. 184.5 °C–186.0 °C. ^1^H NMR (500 MHz, CD_3_OD) *δ* 3.10 (d, *J* = 13.5 Hz, 1H), 3.02 (d, *J* = 13.5 Hz, 1H), 2.80 (d, *J* = 16.5 Hz, 1H), 2.76 (d, *J* = 17.0 Hz, 1H), 2.26–2.30 (m, 1H), 1.92–1.99 (m, 2H), 1.72 (dd, *J* = 14.0 Hz and 7.5 Hz, 1H), 1.51–1.67 (m, 6H), 1.40–1.48 (m, 2H); ^13^C NMR (126 MHz, CD_3_OD) *δ* 176.62, 48.26, 45.68, 41.36, 40.49, 38.79, 37.42, 35.80, 33.45, 28.93, 20.04; ESI-HR-MS: (*m*/*z*) calcd. for C_11_H_20_NO_2_ ([M+H]^+^) 198.1489, found: 198.1489.

(1S*,5R*)-6-(Nitromethylene)bicyclo[3.2.1]octane (**45**). Following the procedure for the synthesis of **11** from **9**, **45** was prepared from **40** (1.00 g, 8.05 mmol) with CH_3_NO_2_ (7.5 mL) and piperidine (0.34 g, 3.99 mmol) in refluxing benzene (20 mL). Pale-yellow oil, 0.40 g (30%). Compound **50** was a mixture of *E*/*Z* geometric isomers and was used directly in the next step without further purification and characterization.

*tert*-Butyl 2-((1S*,5R*,6R*)-6-(nitromethyl)bicyclo[3.2.1]octan-6-yl)acetate (**46**). Following the procedure for the synthesis of **12** from **11**, **46** was prepared from **45** (0.40 g, 2.39 mmol) with diisopropylamine (0.72 g, 7.12 mmol), *n*-BuLi (1.6 M solution in THF, 4.5 mL, 7.20 mmol) and CH_3_CO_2_Bu*^t^* (0.83 g, 7.15 mmol) in dried THF (9 mL). Colorless oil, 0.40 g (59%). ^1^H NMR (500 MHz, CDCl_3_) *δ* 4.97 (d, *J* = 12.5 Hz, 1H), 4.91 (d, *J* = 12.5 Hz, 1H), 2.44 (d, *J* = 3.0 Hz, 2H), 2.26–2.30 (m, 1H), 2.11–2.13 (m, 1H), 1.94–1.97 (m, 1H), 1.77 (dd, *J* = 14.5 Hz and 7.5 Hz, 1H), 1.68–1.73 (m, 1H), 1.57–1.60 (m, 2H), 1.50–1.55 (m, 3H), 1.47 (d, *J* = 4.5 Hz, 1H), 1.45 (s, 9H), 1.37 (d, *J* = 12.0 Hz, 1H). ESI-HR-MS: (*m*/*z*) calcd. for C_15_H_25_NO_4_Na ([M+Na]^+^) 306.1676, found: 306.1678.

2-((1S*,5R*,6R*)-6-(Nitromethyl)bicyclo[3.2.1]octan-6-yl)acetic acid (**47**). Following the procedure for the synthesis of **13** from **12**, **47** was prepared from **46** (0.84 g, 2.96 mmol) with TFA (10 mL) in CH_2_Cl_2_ (10 mL). White solid, 0.50 g (74%). m.p. 100.7 °C–102.6 °C. ^1^H NMR (500 MHz, CDCl_3_) *δ* 5.00 (d, *J* = 12.5 Hz, 1H), 4.93 (d, *J* = 12.5 Hz, 1H), 3.75 (bs, 1H), 2.66 (d, *J* = 17.5 Hz, 1H), 2.62 (d, *J* = 17.5 Hz, 1H), 2.31 (s, 1H), 2.12–2.14 (m, 1H), 1.94–1.98 (m, 1H), 1.76 (dd, *J* = 14.5 Hz and 7.5 Hz, 1H), 1.68–1.72 (m, 1H), 1.49–1.66 (m, 6H), 1.40 (d, *J* = 12.0 Hz, 1H); ^13^C NMR (126 MHz, CDCl_3_) *δ* 176.43, 77.57, 45.62, 42.19, 40.93, 39.50, 38.31, 34.49, 32.40, 27.72, 19.10; ESI-HR-MS: (*m*/*z*) calcd. for C_11_H_16_NO_4_ ([M-H]^−^) 226.1085, found: 226.1082.

2-((1S*,5R*,6R*)-6-(Aminomethyl)bicyclo[3.2.1]octan-6-yl)acetic acid (**6**). Following the procedure for the synthesis of **1** from **13**, **6** was prepared from **47** (0.50 g, 2.20 mmol) with Pd(OH)_2_ (50 mg) in MeOH (10 mL). White solid, 60 mg (14%). m.p. 149.3 °C–154.0 °C. ^1^H NMR (500 MHz, CD_3_OD) *δ* 3.25 (d, *J* = 13.0 Hz, 1H), 3.16 (d, *J* = 13.0 Hz, 1H), 2.50 (d, *J* = 15.0 Hz, 1H), 2.46 (d, *J* = 15.0 Hz, 1H), 2.27–2.31 (m, 1H), 2.07–2.12 (m, 1H), 1.88 (s, 1H), 1.77 (dd, *J* = 14.0 Hz and 7.5 Hz, 1H), 1.51–1.68 (m, 6H), 1.48 (d, *J* = 14.0 Hz, 1H), 1.36 (d, *J* = 11.5 Hz, 1H); ^13^C NMR (126 MHz, CD_3_OD) *δ* 180.00, 51.70, 46.16, 46.08, 43.89, 39.61, 39.45, 36.20, 33.57, 28.81, 19.93; ESI-HR-MS: (*m*/*z*) calcd. for C_11_H_20_NO_2_ ([M+H]^+^) 198.1489, found: 198.1490.

(1S*,5S*)-3-Bromobicyclo[3.2.1]oct-3-en-6-one (**48**). Following the procedure for the synthesis of **40** from **39**, **48** was prepared from **38** (19.00 g, 93.6 mmol) with a Jones reagent prepared from CrO_3_ (14.00 g, 0.140 mol), H_2_O (42 mL) and concentrated sulfuric acid (12.7 mL) in acetone (120 mL) at 0 °C. Pale-yellow oil, 5.00 g (27%). ^1^H NMR (500 MHz, CDCl_3_) *δ* 6.06 (dt, *J* = 8.0 Hz and 1.0 Hz, 1H), 2.94–2.99 (m, 1H), 2.88–2.91 (m, 1H), 2.63–2.67 (m, 1H), 2.36–2.44 (m, 2H), 2.28 (dd, *J* = 18.8 Hz and 3.2 Hz, 1H), 1.94–1.98 (m, 1H), 1.84 (dd, *J* = 12.0 Hz and 3.5 Hz, 1H). The ^1^H NMR data were consistent with those reported in [31].

(1S*,5S*)-3-Bromospiro[bicyclo[3.2.1]octane-6,2′-[1,3]dioxolan]-3-ene (**49**). To a stirred solution of compound **48** (5.00 g, 24.9 mmol) in toluene (50 mL) were added *p*-TsOH•H_2_O (0.95 g, 4.99 mmol) and ethylene glycol (3.10, 49.9 mmol). The reaction mixture was refluxed and produced water was removed by a Dean–Stark apparatus. The reaction was complete within the typical 12 h, as indicated by TLC analysis. On cooling to room temperature, the reaction mixture was poured into ice water (100 mL) and the obtained mixture was adjusted to pH 7 with aqueous NaHCO_3_. The resulting mixture was extracted with EtOAc (50 mL × 3), and the combined extracts were washed with brine (50 mL), dried over anhydrous MgSO_4_ and evaporated on a rotary evaporator to afford a residue, which was purified by column chromatography (silica gel, EtOAc/*n*-hexane = 1/10) to give **49**. Pale-yellow oil, 5.10 g (84%). ^1^H NMR (500 MHz, CDCl_3_) *δ* 6.09 (dt, *J* = 7.0 Hz and 1.0 Hz, 1H), 3.84–3.97 (m, 4H), 2.77–2.82 (m, 1H), 2.35–2.38 (m, 2H), 2.28–2.32 (m, 1H), 2.14–2.19 (m, 1H), 1.89–1.94 (m, 2H), 1.63 (dd, *J* = 11.2 Hz and 2.8 Hz, 1H). The ^1^H NMR data were consistent with those reported in [31].

(1S*,5S*)-Spiro[bicyclo[3.2.1]octane-6,2′-[1,3]dioxolan]-3-ene (**50**). To a stirred solution of compound **49** (1.70 g, 6.94 mmol) in dried THF (20 mL) cooled at −78°C under N_2_, *n*-BuLi (1.6 M solution in THF, 13 mL, 20.8 mmol) was added dropwise. After addition, the reaction mixture was stirred at −78°C for 2 h, after which TLC analysis indicated the completion of the reaction. The reaction mixture was then quenched by the dropwise addition of water (5 mL) at this temperature, and the resulting mixture was poured into saturated aqueous NH_4_Cl (50 mL). The obtained mixture was extracted with EtOAc (20 mL × 3), and the combined extracts were dried over anhydrous MgSO_4_ and evaporated on a rotary evaporator to afford a residue, which was purified by column chromatography (silica gel, EtOAc/*n*-hexane = 1/20) to give **50**. Colorless oil, 0.65 g (56%). ^1^H NMR (500 MHz, CDCl_3_) *δ* 5.77–5.81 (m, 1H), 5.58–5.61 (m, 1H), 3.87–3.99 (m, 4H), 2.40–2.46 (m, 1H), 2.29–2.36 (m, 2H), 2.12–2.17 (m, 1H), 1.93–1.98 (m, 1H), 1.88–1.91 (m, 1H), 1.85 (dd, *J* = 14.5 Hz and 2.5 Hz, 1H), 1.59–1.61 (m, 1H).

(1S*,5S*)-Bicyclo[3.2.1]oct-3-en-6-one (**51**). To a stirred solution of compound **50** (0.65 g, 3.91 mmol) in acetone (7 mL), *p*-TsOH (0.80 g, 4.65 mmol) and H_2_O (0.35 mL) were added. The reaction mixture was refluxed for 24 h, after which TLC analysis indicated the completion of the reaction. On cooling to room temperature, the reaction mixture was poured into aqueous NaHCO_3_ (20 mL) and the obtained mixture was extracted with EtOAc (10 mL × 3). The combined extracts were washed with brine (10 mL), dried over anhydrous MgSO_4_ and evaporated on a rotary evaporator to afford a residue, which was purified by column chromatography (silica gel, EtOAc/*n*-hexane = 1/20) to give **51**. Yellow oil, 0.27 g (57%). ^1^H NMR (500 MHz, CDCl_3_) *δ* 5.74 (d, *J* = 4.5 Hz, 2H), 2.83–2.85 (m, 1H), 2.57–2.64 (m, 2H), 2.38 (dd, *J* = 18.5 Hz and 7.5 Hz, 1H), 2.19 (dd, *J* = 18.5 Hz and 3.5 Hz, 1H), 2.02 (d, *J* = 17.5 Hz, 1H), 1.90–1.95 (m, 1H), 1.76 (dd, *J* = 11.5 Hz and 3.5 Hz, 1H); ^13^C NMR (126 MHz, CDCl_3_) *δ* 213.08, 128.52, 126.13, 46.98, 43.88, 34.51, 32.13, 30.43; ESI-HR-MS: (*m*/*z*) calcd. for C_8_H_11_O ([M+H]^+^) 123.0804, found: 123.0807. The ^1^H NMR [31] and ^13^C NMR [32] data were in consistent with those reported.

(1S*,5S*)-7-(Nitromethylene)bicyclo[3.2.1]oct-2-ene (**52**). Following the procedure for the synthesis of **11** from **9**, **52** was prepared from **51** (1.18 g, 9.66 mmol) with CH_3_NO_2_ (5 mL) and piperidine (0.47 g, 5.52 mmol) in refluxing benzene (10 mL). Yellow oil, 0.80 g (50%). Compound **57** was a mixture of *E*/*Z* geometric isomer and was used directly in the next step without further purification and characterization.

*tert*-Butyl 2-((1S*,5S*,6R*)-6-(nitromethyl)bicyclo[3.2.1]oct-3-en-6-yl)acetate (**53**). Following the procedure for the synthesis of **12** from **11**, **53** was prepared from **52** (0.80 g, 4.84 mmol) with diisopropylamine (1.50 g, 14.8 mmol), *n*-BuLi (1.6 M solution in THF, 9 mL, 14.4 mmol) and CH_3_CO_2_Bu*^t^* (1.69 g, 14.5 mmol) in dried THF (14 mL). Colorless oil, 0.50 g (37%). ^1^H NMR (500 MHz, CDCl_3_) *δ* 5.90–5.93 (m, 1H), 5.63–5.66 (m, 1H), 4.90 (d, *J* = 12.5 Hz, 1H), 4.82 (d, *J* = 12.5 Hz, 1H), 2.47 (d, *J* = 1.5 Hz, 2H), 2.38–2.44 (m, 3H), 1.97–2.02 (m, 1H), 1.90–1.94 (m, 1H), 1.84–1.89 (m, 1H), 1.67 (d, *J* = 11.0 Hz, 1H), 1.46 (s, 9H), 1.37 (d, *J* = 14.0 Hz, 1H); ^13^C NMR (126 MHz, CDCl_3_) *δ* 170.84, 131.35, 127.78, 81.26, 79.80, 52.40, 42.65, 42.55, 41.50, 37.83, 33.51, 33.41, 28.24; ESI-HR-MS: (*m*/*z*) calcd. for C_15_H_23_NNaO_4_ ([M+Na]^+^) 304.1519, found: 304.1521.

*tert*-Butyl 2-((1S*,5S*,6R*)-6-(aminomethyl)bicyclo[3.2.1]oct-3-en-6-yl)acetate (**54**) *p*-tosylate. Following the procedure for the synthesis of **19**•*p*-TsOH from **18**, **54**•*p*-TsOH was prepared from **53** (0.50 g, 1.78 mmol) with iron powder (0.50 g, 8.95 mmol) and NH_4_Cl (0.20 g, 3.74 mmol) in a mixed solvent of EtOH (5 mL) and H_2_O (2.5 mL). White solid, 178 mg (24%). m.p. 176.3 °C–177.7 °C. ^1^H NMR (500 MHz, DMSO-d_6_) *δ* 7.68 (bs, 3H), 7.48 (d, *J* = 8.0 Hz, 2H), 7.11 (d, *J* = 8.0 Hz, 2H), 5.87–5.90 (m, 1H), 5.59–5.62 (m, 1H), 3.11–3.18 (m, 1H), 3.02–3.08 (m, 1H), 2.42 (s, 2H), 2.31–2.34 (m, 2H), 2.29 (s, 3H), 2.13–2.15 (m, 1H), 1.89–1.93 (m, 1H), 1.75–1.84 (m, 2H), 1.50 (d, *J* = 11.5 Hz, 1H), 1.42 (s, 9H), 1.23 (d, *J* = 13.5 Hz, 1H); ^13^C NMR (126 MHz, DMSO-d_6_) *δ* 170.44, 145.76, 137.58, 130.61, 128.04, 127.32, 125.48, 80.59, 51.40, 44.00, 41.79, 40.98, 40.44, 37.12, 33.08, 32.91, 27.79, 20.77; ESI-HR-MS: (*m*/*z*) calcd. for C_15_H_26_NO_2_ ([M+H]^+^) 252.1958, found: 252.1959.

2-((1S*,5S*,6R*)-6-(Aminomethyl)bicyclo[3.2.1]oct-3-en-6-yl)acetic acid (**7**) *p*-tosylate. Following the procedure for the synthesis of **2**•*p*-TsOH from **19**•*p*-TsOH, **7**•*p*-TsOH was prepared from **54**•*p*-TsOH (0.17 g, 0.401 mmol) with TFA (2 mL) in CH_2_Cl_2_ (3 mL). White solid, 90 mg (61%). m.p. 188.5 °C–189.6 °C. ^1^H NMR (500 MHz, D_2_O) *δ* 7.70 (d, *J* = 8.5 Hz, 2H), 7.38 (d, *J* = 8.5 Hz, 2H), 5.86–5.89 (m, 1H), 5.70–5.73 (m, 1H), 3.36 (s, 2H), 2.61 (s, 2H) 2.38–2.43 (m, 5H), 2.27–2.29 (m, 1H), 1.98–2.03 (m, 1H), 1.86–1.93 (m, 2H), 1.67 (d, *J* = 11.5 Hz, 1H), 1.36 (d, *J* = 14.0 Hz, 1H); ^13^C NMR (126 MHz, D_2_O) *δ* 175.95, 142.47, 139.35, 129.73, 129.41, 128.64, 125.32, 50.86, 45.45, 42.32, 41.34, 41.09, 37.05, 33.22, 33.03, 20.44; ESI-HR-MS: (*m*/*z*) calcd. for C_11_H_18_NO_2_ ([M+H]^+^) 196.1332, found: 196.1334.

Cycloheptanol (**56**). To a solution of cycloheptanone (**55**, 69.00 g, 0.615 mol) in a mixed solvent of methanol (450 mL) and water (45 mL) cooled at 0 °C was added NaBH_4_ (11.63 g, 0.307 mol) in a portionwise manner. After addition, the reaction mixture was stirred for another 2 h at room temperature, after which TLC analysis indicated the completion of the reaction. The reaction mixture was evaporated on a rotary evaporator to afford a residue, which was diluted in aqueous NaOH (1 M, 300 mL), and the resulting mixture was extracted with EtOAc (200 mL × 3). The combined extracts were washed successively with aqueous NaOH (1 M, 100 mL) and brine (100 mL), dried over anhydrous MgSO_4_ and evaporated on a rotary evaporator to give **56**. Colorless oil, 64.77 g (92%). ^1^H NMR (500 MHz, CDCl_3_) *δ* 3.82–3.87 (m, 1H), 1.89–1.94 (m, 2H), 1.62–1.68 (m, 2H), 1.57–1.59 (m, 1H), 1.52–1.56 (m, 5H), 1.37–1.44 (m, 2H). The ^1^H NMR data were consistent with those reported in [33].

Cycloheptene (**57**). Compound **56** (64.77 g, 0.567 mol) was mixed with *p*-TsOH•H_2_O (0.70 g, 3.68 mmol), and the mixture was then stirred at 160 °C for 11 h with the removal of produced water by a Dean–Stark apparatus. On cooling to room temperature, the reaction mixture was washed successively with aqueous NaHCO_3_ (100 mL) and brine (100 mL) and dried over anhydrous MgSO_4_ to give **57**. Colorless oil, 57.42 g (100%). ^1^H NMR (500 MHz, CDCl_3_) *δ* 5.78–5.80 (m, 2H), 2.10–2.14 (m, 4H), 1.70–1.75 (m, 2H), 1.48–1.53 (m, 4H). The ^1^H NMR data were consistent with those reported in [34].

3-Bromocyclohept-1-ene (**58**). To a stirred solution of compound **57** (58.30 g, 0.606 mol) in CCl_4_ (400 mL), NBS (107.89 g, 0.606 mol) was added. The reaction mixture was heated to 65 °C followed by the addition of AIBN (0.99 g, 6.03 mmol) in portions. After addition, the reaction mixture was heated slowly to 75 °C and the heating was stopped immediately once the reaction mixture began to boil. Approximately 10 min later, the boiling subsided and the reaction mixture was refluxed for another 1 h, after which TLC analysis indicated the completion of the reaction. The reaction mixture was cooled to room temperature and filtered off through celite. The filtrate was washed successively with aqueous Na_2_S_2_O_3_ (100 mL × 4) and brine (100 mL), dried over anhydrous MgSO_4_ and evaporated on a rotary evaporator to give a pale-yellow oil (crude **58**, 133.23 g), which was used directly in the next step without further purification and characterization.

Cyclohepta-1,3-diene (**59**). Crude **58** (133.23 g) and quinoline (225 mL) were placed into a round-bottomed flask equipped with a fractional column. The reaction mixture was heated to 165 °C and the produced **59** was distilled out of the reaction mixture and purified by fractional distillation at atmospheric pressure. Colorless oil, 40.03 g. Crude **59** was used directly in the next step without further purification and characterization.

(3aR*,4S*,8R*,8aS*)-4,5,6,7,8,8a-Hexahydro-1H-4,8-ethenocyclohepta[c]furan-1,3(3aH)-dione (**60**). A stirred solution of compound **59** (15.00 g, 0.159 mol) and maleic anhydride (15.62 g, 0.159 mol) in toluene (80 mL) was refluxed under N_2_ overnight, after which TLC analysis indicated the completion of the reaction. On cooling to room temperature, the reaction mixture was evaporated on a rotary evaporator to afford a residue, which was purified by column chromatography (silica gel, EtOAc/*n*-hexane = 1/5) to give **60**. White solid, 14.24 g (overall 33% for **57** to **60**). ^1^H NMR (500 MHz, DMSO-d_6_) *δ* 6.17 (dd, *J* = 5.0 Hz and 3.5 Hz, 2H), 3.59 (s, 2H), 2.80–2.83 (m, 2H), 1.62–1.68 (m, 4H), 1.46–1.49 (m, 2H). The ^1^H NMR data were consistent with those [35].

(3aR*,4S*,8R*,8aS*)-Hexahydro-1H-4,8-ethanocyclohepta[c]furan-1,3(3aH)-dione (**61**). A mixture of compound **60** (14.24 g, 73.3 mmol) and 10% Pd/C (1.42 g) in MeOH (100 mL) was subjected to a procedure of standard hydrogenation at atmospheric pressure (balloon) at room temperature overnight, after which TLC analysis indicated the completion of the reaction. The reaction mixture was filtered off through celite, and the filtrate was evaporated on a rotary evaporator to give **61**. White solid, 14.18 g (99%). Compound **61** was used directly in the next step without characterization.

(1R*,5S*,6R*,7S*)-Bicyclo[3.2.2]nonane-6,7-dicarboxylic acid (**62**). A solution of compound **61** (14.18 g, 73.0 mmol) in 10% aqueous NaOH (150 mL) was stirred at room temperature until the solid was completely dissolved. The mixture was acidified by aqueous 20% H_3_PO_4_ to pH 1 and a large amount of precipitates formed. The precipitates were collected via suction filtration, washed with water and dried in vacuo at room temperature to give **62**. White solid, 12.98 g (84%). ^1^H NMR (500 MHz, DMSO-d_6_) *δ* 11.88 (bs, 2H), 2.85 (s, 2H), 2.35 (s, 2H), 1.74–1.78 (m, 2H), 1.59–1.69 (m, 3H), 1.48–1.57 (m, 3H), 1.42–1.46 (m, 2H). The ^1^H NMR data were consistent with those reported in [36].

(1S*)-Bicyclo[3.2.2]non-6-ene (**63**). Compound **62** (5.00 g, 23.6 mmol) was added to a stirred mixture of Pb(OAc)_4_ (15.67 g, 35.3 mmol) in pyridine (100 mL) under N_2_. The reaction mixture was stirred at 75 °C for 30 min, after which TLC analysis indicated the completion of the reaction. On cooling to room temperature, the reaction mixture was poured into water (300 mL) and the obtained mixture was extracted with pentane (100 mL × 4). The combined extracts were washed successively with 1 M HCl (200 mL × 3) and brine (200 mL), dried over anhydrous MgSO_4_ and evaporated on a rotary evaporator at 20 °C to give **63**. Pale-yellow oil, 2.90 g (100%). Compound **63** was used directly in the next step without characterization.

(1S*,6R*)-Bicyclo[3.2.2]nonan-6-ol (**64**). To a stirred solution of compound **63** (3.52 g, 28.8 mmol) in dried THF (25 mL) cooled at 0 °C, BH_3_•Me_2_S (2.0 M solution in THF, 6.0 mL, 12.0 mmol) was added dropwise under N_2_. After addition, the reaction mixture was stirred for 2 h at room temperature, after which TLC analysis indicated the completion of the reaction. EtOH (25 mL) was added to the reaction mixture. The resulting mixture was cooled to 0 °C, followed by dropwise addition of aqueous NaOH (3 M, 4 mL) and 30% H_2_O_2_ (3.6 mL) successively. After addition, the reaction mixture was stirred at room temperature for 6 h, when TLC analysis indicated the completion of the reaction. The reaction mixture was poured into ice water (50 mL) and the obtained mixture was extracted with EtOAc (20 mL × 3). The combined extracts were washed with 1 M HCl (50 mL) and brine (50 mL), dried over anhydrous MgSO_4_ and evaporated on a rotary evaporator to give a residue, which was purified by column chromatography (silica gel, EtOAc/*n*-hexane = 1/10) to give **64**. White solid, 3.92 g (97%). ^1^H NMR (500 MHz, CDCl_3_) *δ* 4.05–4.08 (m, 1H), 2.13–2.18 (m, 1H), 1.92–1.99 (m, 2H), 1.85–1.88 (m, 1H), 1.69–1.76 (m, 1H), 1.58–1.65 (m, 5H), 1.50–1.56 (m, 2H), 1.43–1.48 (m, 2H), 1.33 (bs, 1H). The ^1^H NMR data were consistent with those reported in [37].

(1S*)-Bicyclo[3.2.2]nonan-6-one (**65**). Following the procedure for the synthesis of **40** from **39**, **65** was prepared from **64** (3.92 g, 28.0 mmol) with CrO_3_ (4.19 g, 41.9 mmol), H_2_O (15 mL) and concentrated H_2_SO_4_ (3.8 mL) in acetone (25 mL). White solid, 1.91 g (49%). ^1^H NMR (500 MHz, CDCl_3_) *δ* 2.51–2.54 (m, 1H), 2.31–2.34 (m, 3H), 1.88–1.92 (m, 2H), 1.75–1.82 (m, 4H), 1.70–1.73 (m, 1H), 1.64–1.68 (m, 2H), 1.47–1.53 (m, 1H); ^13^C NMR (126 MHz, CDCl_3_) *δ* 217.32, 47.01, 44.41, 34.21, 30.96, 28.52, 24.40, 23.25, 21.65; ESI-HR-MS: (*m*/*z*) calcd. for C_9_H_15_O ([M+H]^+^) 139.1117, found: 139.1118.

Dimethyl 2-((1S*)-bicyclo[3.2.2]nonan-6-ylidene)malonate (**66**). Following the procedure for the synthesis of **41** from **40**, crude **66** was prepared from **65** (1.91 g, 13.8 mmol) with TiCl_4_ (7.86 g, 41.4 mmol), CH_2_(CO_2_CH_3_)_2_ (5.48 g, 41.5 mmol) and pyridine (6.56 g, 82.9 mol) in a mixed solvent of dried THF (12 mL) and dried CH_2_Cl_2_ (60 mL). Colorless oil, 3.34 g. The crude sample of **66** was used in the next step without further purification and characterization.

Methyl 2-((1S*,6S*)-6-cyanobicyclo[3.2.2]nonan-6-yl)acetate (**67**). To a stirred mixture of crude **66** (3.69 g, deemed to be 14.6 mmol) and K_2_CO_3_ (2.43 g, 17.6 mmol) in MeOH (40 mL) cooled at 0 °C under N_2_ TMSCN (6.53 g, 65.8 mmol) was added dropwise. After addition, the reaction mixture was refluxed for 24 h, when TLC analysis indicated the completion of the reaction. On cooling to room temperature, the reaction mixture was poured into ice water (100 mL) and the obtained mixture was extracted with EtOAc (30 mL × 3). The combined extracts were washed with aqueous NaHCO_3_ (50 mL) and brine (50 mL), dried over anhydrous MgSO_4_ and evaporated on a rotary evaporator to afford a residue, which was purified by column chromatography (silica gel, EtOAc/*n*-hexane = 1/10) to give **67**. White solid, 0.99 g (overall 29% for **65** to **67**). m.p. 58 °C–59 °C; ^1^H NMR (500 MHz, CDCl_3_) *δ* 3.75 (s, 3H), 2.75 (d, *J* = 15.5 Hz, 1H), 2.64 (d, *J* = 16.0 Hz, 1H), 2.43 (dd, *J* = 15.0 Hz and 7.5 Hz, 1H), 2.19–2.28 (m, 2H), 2.12–2.15 (m, 1H), 1.83–1.91 (m, 1H), 1.71–1.80 (m, 2H), 1.55–1.70 (m, 6H), 1.45–1.52 (m, 1H); ^13^C NMR (126 MHz, CDCl_3_) *δ* 170.03, 125.23, 52.12, 42.86, 38.89, 36.19, 36.04, 34.57, 29.64, 28.83, 24.12, 23.51, 21.20; ESI-HR-MS: (*m*/*z*) calcd. for C_13_H_20_NO_2_ ([M+H]^+^) 222.1489, found: 222.1489.

(1S*,6S*)-Spiro[bicyclo[3.2.2]nonane-6,3′-pyrrolidin]-5′-one (**68**). Following the procedure for the synthesis of **44** from **43**, **68** was prepared from **67** (1.30 g, 5.87 mmol) with NiCl_2_•6H_2_O (2.79 g, 11.7 mmol) and NaBH_4_ (2.22 g, 58.7 mmol) in MeOH (30 mL) at 0 °C to room temperature. White solid, 0.60 g (53%). m.p. 127.8 °C–129.4 °C. ^1^H NMR (500 MHz, CDCl_3_) *δ* 5.84 (bs, 1H), 3.29 (d, *J* = 9.0 Hz, 1H), 3.18 (d, *J* = 9.0 Hz, 1H), 2.33 (d, *J* = 16.0 Hz, 1H), 2.16 (d, *J* = 16.0 Hz, 1H), 2.04–2.07 (m, 1H), 1.94 (dd, *J* = 14.5 Hz and 2.0 Hz, 1H), 1.88 (s, 2H), 1.77–1.81 (m, 1H), 1.69–1.76 (m, 2H), 1.59–1.66 (m, 6H), 1.48–1.54 (m, 1H); ^13^C NMR (126 MHz, CDCl_3_) *δ* 178.52, 57.86, 46.56, 42.40, 41.98, 38.88, 35.53, 31.84, 29.62, 23.78, 23.03, 21.75; ESI-HR-MS: (*m*/*z*) calcd. for C_12_H_20_NO ([M+H]^+^) 194.1539, found: 194.1541.

2-((1S*,6S*)-6-(Aminomethyl)bicyclo[3.2.2]nonan-6-yl)acetic acid (**8**) hydrobromide. Following the procedure for the synthesis of **5**•HBr from **44**, **8**•HBr was prepared from **68** (0.48 g, 2.48 mmol) in refluxing 40% HBr (10 mL) under N_2_. White solid, 81 mg (11%). m.p. 175.8 °C–179.8 °C. ^1^H NMR (500 MHz, CD_3_OD) *δ* 3.14 (d, *J* = 13.5 Hz, 1H), 3.11 (d, *J* = 13.5 Hz, 1H), 2.63 (d, *J* = 15.5 Hz, 1H), 2.59 (d, *J* = 15.5 Hz, 1H), 2.06–2.08 (m, 1H), 1.95–2.00 (m, 1H), 1.82–1.91 (m, 2H), 1.57–1.74 (m, 9H), 1.52 (dd, *J* = 15.0 Hz and 6.5 Hz, 1H); ^13^C NMR (126 MHz, CD_3_OD) *δ* 175.61, 48.60, 42.41, 38.36, 38.11, 35.15, 34.87, 32.40, 30.67, 25.00, 23.80, 22.32; ESI-HR-MS: (*m*/*z*) calcd. for C_12_H_22_NO_2_ ([M+H]^+^) 212.1645, found: 212.1647.

### 3.2. BCAT1 Enzyme Inhibition Assay

The compounds to be tested or PBS was added to a 384-well plate, followed by the addition of assay buffer (50 mM ammonium sulfate, 1 mM EDTA, 2 mM DTT, 0.01% BSA, 0.5 μM PLP, 50 mM HEPES buffer (pH 7.4)), 0.01 U LeuDH enzyme (#E0217, Nanjing Duly Biotechnology Co., Ltd., Nanjing, China) and 15 ng BCAT1 enzyme (#ME17NO0265, Sino Biological, Beijing, China). Subsequently, 40 μM α-KG, 100 μM Leucine and 10 μM NADH substrate mixture were added to initiate the reaction. Fluorescence changes in NADH were dynamically monitored using the Spark cyto (Tecan, Männedorf, Switzerland). The rate of NADH fluorescence decay was calculated to assess enzyme activity.

### 3.3. Cell Proliferation Assay

Cell culture and chemical reagents: Human NSCLC NCI-H1975 cells were purchased from American Type Culture Collection (ATCC, Manassas, VA, USA) and cultured in RPMI 1640 medium with 10% FBS in a cell incubator (37 °C, 5% CO_2_). ASK120067-resistant cells (67R) were constructed, cultured, and authenticated as described previously [26]. Gabapentin (#T0702) was purchased from TargetMol (Boston, MA, USA).

A sulforodamine B (SRB) colorimetric assay was employed to detect the inhibitory effect of compounds on cell proliferation. Cells in the logarithmic growth phase were seeded into 96-well plates and cultured overnight. After incubation with the indicated compounds for 120 h, the culture medium was discarded. Then, 100 μL of 10% pre-cooled trichloroacetic acid (TCA) was added to each well to fix the cells at 4 °C for 1 h. The wells were then washed five times with distilled water. After drying, 100 μL of SRB dye (4 mg/mL SRB dissolved in 1% glacial acetic acid) was added to each well, and the cells were stained for 15 min at room temperature. After washing with 1% glacial acetic acid for five times, the plate was dried again. To dissolve the dye, 150 μL of 10 mM Tris solution was added to each well. The optical density (OD) was measured at 560 nm using a multi-well VersaMax spectrophotometer (Molecular Devices, Sunnyvale, CA, USA). The relative inhibition rate of the compounds on cell proliferation was calculated using the following formula: Inhibition rate (%) = [1 − (A560_treated_/A560_control_)] × 100%. The IC_50_ values were determined from the dose–response curves using the logit method.

## 4. Conclusions

In summary, inspired by the unique chemical structure of **WQQ-345** discovered in our earlier study, we proceeded to carry out two rounds of SAR study to further explore the optimal structure based on **WQQ-345**, and a bicyclo[3.2.1]octene-bearing GABA derivative **7** was discovered, which was 6-fold more potent than the parent compound **WQQ-345** in the BCAT1 enzymatic inhibitory activity assay, effectively suppressed the growth of 67R cells that highly expressed BCAT1, and was resistant to third-generation TKI.

## Figures and Tables

**Table 1:** BCAT1 inhibitory activity of synthesized compounds **1**–**8**, **WQQ-345** and gabapentin.

Compound	BCAT1 Inhibitory Activity (IC_50_, mM) ^1^
**WQQ-345**•HBr	4.86 ± 1.98
**1**	2.04 ± 0.59
**2**•*p*-TsOH	2.04 ± 0.98
**3**•*p*-TsOH	3.96 ± 2.83
**4**•HBr	1.71 ± 1.00
**5**•HBr	4.20 ± 1.84
**6**	1.54 ± 0.47
**7**•*p*-TsOH	0.78 ± 0.19
**8**•HBr	14.52 ± 6.39
gabapentin	5.07 ± 1.06

^1^ IC_50_ values are expressed as mean ± SD (n = 3).

**Table 2:** Summary of IC_50_ values of proliferation inhibition of BCAT1 inhibitors **1**, **2**, **4**, **7** and gabapentin in 67R cells and NCI-H1975 cells for 120 h (n = 3).

Compound	NCI-H1975 IC_50_ (mM) ^1^	67R IC_50_ (mM) ^1^
**1**	7.82 ± 0.48	2.60 ± 0.93
**2**•*p*-TsOH	8.99 ± 0.67	2.71 ± 0.33
**4**•HBr	8.08 ± 0.96	1.38 ± 0.37
**7**•*p*-TsOH	7.26 ± 0.73	1.64 ± 0.49
gabapentin	>10	3.53 ± 0.22

^1^ IC_50_ values are expressed as mean ± SD (n = 3).

## Data Availability

The data presented in this study are available on request from the corresponding authors.

## Supplementary Materials

The following supporting information can be downloaded at: https://www.mdpi.com/article/10.3390/molecules30040904/s1. The synthetic procedure of **WQQ-345** hydrobromide; single-crystal X-ray diffraction results of compounds **44**, **68** and **S4**; and copies of all the ^1^H NMR, ^13^C NMR and HR-MS spectra for synthesized compounds.

## Abbreviations

The following abbreviations are used in this manuscript:

AIBN	2,2′-azodiisobutyronitrile
BCAA	branched-chain amino acid
BCAT	branched-chain amino acid aminotransferase
BCAT1	branched-chain amino acid aminotransferase 1
BCAT2	branched-chain amino acid aminotransferase 2
CCDC	Cambridge Crystallographic Data Centre
EGFR	epidermal growth factor receptor
ESI	electrospray ionization
GABA	γ-aminobutyric acid
HR-MS	high-resolution mass spectrometry
α-KIC	α-ketoisocaproate
LDA	lithium diisopropylamide
LeuDH	leucine dehydrogenase
NADH	nicotinamide adenine dinucleotide
NBS	*N*-bromosuccinimide
NSCLC	non-small-cell lung cancer
SAR	structure–activity relationship
TFA	trifluoroacetic acid
TLC	thin-layer chromatography
TKI	tyrosine kinase inhibitor
