# Supplementary material for: Design, Synthesis and Biological Activity Study of γ-Aminobutyric Acid (GABA) Derivatives Containing Bridged Bicyclic Skeletons as BCAT1 Inhibitors
Source: Molecules. 2025 Feb 15;30(4):904. doi: 10.3390/molecules30040904 (PMC11858294; doi:10.3390/molecules30040904)
Supplement: Supplementary file 1 [file molecules-30-00904-s001.zip › molecules-3454798-supplementary.pdf]

Supplementary Materials

Design, Synthesis and Biological Activity Study of γ-Aminobutyric Acid (GABA) Derivatives Containing Bridged Bicyclic Skeletons as BCAT1 Inhibitors

Wen Luo [1,2,†], Zilu Pan [3,4,†], Xinyuan Zhu [2,5,†], Yan Li [3,†], Yong Li [1,2], Yudi Zhang [3,4,6], Jiamin Pan [1,2], Jian Ding [3], Hua Xie [1,2,3,4,*] and Guilong Zhao [1,2,3,4,5,*]

[1] School of Pharmaceutical Sciences, Southern Medical University, Guangzhou 510515, China; luowen56286@163.com (W.L.); liyong554@zidd.ac.cn (Y.L.); panjiamin987@zidd.ac.cn (J.P.)
[2] Zhongshan Institute for Drug Discovery, Shanghai Institute of Materia Medica, Chinese Academy of Sciences, Zhongshan 528400, China; zhuxinyuan623@zidd.ac.cn
[3] Shanghai Institute of Materia Medica, Chinese Academy of Sciences, Shanghai 201203, China; s20-panzilu@simm.ac.cn (Z.P.); yanli@simm.ac.cn (Y.L.); zhangyd2022@shanghaitech.edu.cn (Y.Z.); jding@simm.ac.cn (J.D.)
[4] University of Chinese Academy of Sciences, Beijing 100049, China
[5] School of Pharmacy, China Pharmaceutical University, Nanjing 211198, China
[6] School of Life Science and Technology, ShanghaiTech University, Shanghai 201210, China
* Correspondence: hxie@simm.ac.cn (H.X.); zhao_guilong@126.com (G.Z.)
† These authors contributed equally to this work.

1. Chemistry

1.1 General

Unless stated otherwise, all the chemicals were obtained commercially (Bide Pharmatech Co., Ltd., Shanghai, China) and used without further purification. All the dried solvents were prepared by standard methods. Reaction progress was monitored by thin-layer chromatography (TLC) on commercially available precoated TLC silica gel plate (Xinnuo Chemical Co., Ltd., Yantai, China). Purifications on flash column chromatography were performed on silica gel column (200-300 mesh, (Xinnuo Chemical Co., Ltd., Yantai, China)) eluting with EtOAc/*n*-hexane (ratio was expressed as v/v). Melting points were measured in open capillaries with an SGW X-4A microscopic melting point apparatus (Shanghai INESA Physico-Optical Instrument Co., Ltd., Shanghai, China) and are uncorrected. All ^{1}H and ^{13}C NMR spectra were recorded on a Bruker Ascend 500 spectrometer (Bruker Switzerland AG, Fällanden, Switzerland) with $CDCl_3$, DMSO-d_6, CD_3OD or D_2O as solvent and TMS (for ^{1}H NMR) or known chemical shifts of carbon signals of deuterated solvents (for ^{13}C NMR) as internal standard. High-resolution mass spectra (HR-MS) were determined with a Thermo Scientific Exactive Plus mass spectrometer (Thermo Fisher Scientific, Bremen, Germany) using electrospray ionization (ESI) and Orbitrap techniques. Hydrogenolysis/hydrogenation requiring pressures higher than atmospheric pressure were conducted on an AE250 hydrogenator (Labe Instrument Co., Ltd., Shanghai, China) equipped with a TH-5300H high-pressure hydrogen generator (Oushisheng Technology, Co., Ltd., Beijing, China), and the reaction temperature and H_2 pressure could be controlled automatically and continuously. Single-crystal X-ray diffractions were performed on a Bruker APEX-II CCD four-circle diffractometer (Bruker AXS Inc., Madison, WI, USA) equipped with graphite-monochromatic Mo Kα radiation (λ = 0.71073 Å) at 150.00/100.00 K. Reflections were collected by using a φ-ω scan mode. Data reduction and cell refinement were carried out using the SAINT V8.40B (Bruker, 2016) or SAINT 8.38A (Bruker, 2018) software. The structures were solved by direct method and refined with SHELXL 2018[1]. The crystallographic data have been deposited at Cambridge Crystallographic Data Centre (CCDC).

*1.2 Synthesis of **WQQ-345•HBr***

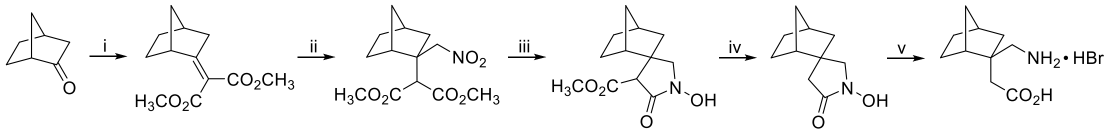

9 **S1** **S2** **S3** **S4** **WQQ-345**·HBr

Scheme S1. Synthetic route to compound **WQQ-345** hydrobromide. *Reagents and conditions*: (i) $CH_2(CO_2CH_3)_2$, $TiCl_4$/pyridine, dried THF/CH_2Cl_2, -15°C-rt, N_2, 72%; (ii) CH_3NO_2, DBU, rt, 61%; (iii) H_2 (balloon), $Pd(OH)_2$, MeOH, rt; (iv) NaCl/H_2O, DMF, reflux, 38% (overall for **S2** to **S4)**; (v) 40% HBr, reflux, N_2, 45%.

Dimethyl 2-((1*R**,4*S**)-bicyclo[2.2.1]heptan-2-ylidene)malonate (**S1**). To a stirred mixed solvent of dried THF (250 mL) and dried CH_2Cl_2 (1000 mL) cooled at -15°C under N_2 were successively added $TiCl_4$ (115.36 g, 0.608 mol) and dimethyl malonate (115.36 g, 0.608 mol) both in a dropwise manner. After addition, a solution of **9** (50.00 g, 0.454 mol) in dried CH_2Cl_2 (250 mL) was added dropwise to the reaction mixture, followed by dropwise addition of pyridine (95.86 g, 1.21 mol). The resulting mixture was stirred at room temperature overnight, when

TLC analysis indicated the completion of the reaction. The reaction mixture was carefully poured into ice-water (1000 mL), and the mixture thus obtained was extracted with CH_2Cl_2 (300 mL × 3). The combined extracts were washed with brine (200 mL), dried over anhydrous $MgSO_4$ and evaporated on a rotary evaporator to afford a residue, which was purified by column chromatography (silica gel, EtOAc/*n*-hexane = 1/20) to give **S1**. Colorless oil, 73.60 g (72%). ^{1}H NMR (500 MHz, $CDCl_3$) δ 3.80 (s, 3H), 3.74 (s, 3H), 3.26 (d, *J* = 5.0 Hz, 1H), 2.57 (dt, *J* = 18.7 and 3.8 Hz, 1H), 2.47 (t, *J* = 4.5 Hz, 1H), 2.37 (dd, *J* = 18.8 Hz and 3.8 Hz, 1H), 1.76-1.82 (m, 1H), 1.62-1.67 (m, 1H), 1.44-1.47 (m, 1H), 1.38-1.43 (m, 2H), 1.23-1.29 (m, 1H); ^{13}C NMR (126 MHz, $CDCl_3$) δ 171.96, 166.88, 165.52, 117.53, 52.17, 51.86, 45.28, 41.07, 39.38, 35.98, 27.97, 27.37; ESI-HR-MS: (*m*/*z*) calcd. for $C_{12}H_{17}O_4$ ([M+H]$^+$) 225.1121, found: 225.1122.

Dimethyl 2-((1*R**,2*R**,4*S**)-2-(nitromethyl)bicyclo[2.2.1]heptan-2-yl)malonate (**S2**). To a stirred solution of compound **S1** (75.20 g, 0.335 mol) in CH_2Cl_2 (200 mL) at room temperature was added CH_3NO_2 (250 mL), followed by dropwise addition of DBU (99.62 g, 0.654 mol) at 0°C. After addition, the reaction mixture was stirred at room temperature overnight, when TLC analysis indicated the completion of the reaction. The reaction mixture was poured into ice-water (500 mL), and the obtained mixture was extracted with CH_2Cl_2 (200 mL × 3). The combined extracts were washed with brine (200 mL), dried over anhydrous $MgSO_4$ and evaporated on a rotary evaporator to afford a residue, which was purified by column chromatography (silica gel, EtOAc/*n*-hexane = 5/95) to give **S2**. Pale yellow oil, 58.40 g (61%). ^{1}H NMR (500 MHz, $CDCl_3$) δ 5.05 (d, *J* = 13.0 Hz, 1H), 4.71 (d, *J* = 12.5 Hz, 1H), 3.75 (s, 3H), 3.74 (s, 3H), 3.52 (s, 1H), 2.52 (t, *J* = 1.5 Hz, 1H), 2.32 (t, *J* = 4.0 Hz, 1H), 1.69-1.73 (m, 1H), 1.60-1.67 (m, 1H), 1.55-1.58 (m, 1H), 1.46-1.54 (m, 3H), 1.31-1.34 (m, 1H), 1.17-1.23 (m, 1H); ^{13}C NMR (126 MHz, $CDCl_3$) δ 169.09, 168.66, 80.48, 56.62, 52.80, 52.59, 46.24, 45.54, 41.39, 37.14, 37.00, 28.03, 24.72; ESI-HR-MS: (*m*/*z*) calcd. for $C_{13}H_{20}NO_6$ ([M+H]$^+$) 286.1285, found: 286.1285.

Methyl (1*R**,2*R**,4*S**)-1'-hydroxy-5'-oxospiro[bicyclo[2.2.1]heptane-2,3'-pyrroli-dine]-4'-carboxylate (**S3**). A mixture of compound **S2** (58.40 g, 0.205 mol) and $Pd(OH)_2$ (6.00 g) in MeOH (500 mL) was subjected to a procedure of standard hydrogenolysis at atmospheric pressure (balloon) at room temperature overnight, when TLC analysis indicated the completion of the reaction. The reaction mixture was filtered off through celite, and the filtrate was evaporated on a rotary evaporator to afford a pale yellow oil (crude **S3**, 50.10 g), which was a mixture of diastereomeric isomers and was used directly in the next step without further purification and characterization.

(1*R**,2*S**,4*S**)-1'-Hydroxyspiro[bicyclo[2.2.1]heptane-2,3'-pyrrolidin]-5'-one (**S4**). To a stirred solution of compound **S3** (50.10 g, 0.276 mol) in DMF (400 mL) was added NaCl (26.30 g, 0.450 mol) and H_2O (8.1 mL). The reaction mixture was refluxed for 6 h under N_2, when TLC analysis indicated the completion of the reaction. On cooling to room temperature, the reaction mixture was diluted with CH_2Cl_2 (500 mL) and filtered off, and the filtrate was washed with water (300 mL × 5). The organic phase was dried over anhydrous $MgSO_4$ and evaporated on a rotary evaporator to afford a white solid, which was triturated with a mixed solvent (30 mL, EtOAc/*n*-hexane = 1/5) to give **S4**. White solid, 14.00 g (overall 38% for **S2** to **S4**). m.p. 131.3°C-133.9°C. ^{1}H NMR (500 MHz, $CDCl_3$) δ 9.76 (bs, 1H), 3.54 (d, *J* = 9.0 Hz, 1H), 3.33 (d, *J* = 9.0 Hz, 1H), 2.54 (d, *J* = 17.0 Hz, 1H), 2.30 (t, *J* = 4.5 Hz, 1H), 2.22 (d, *J* = 17.0 Hz, 1H), 2.07 (s, 1H), 1.77-1.81 (m, 1H), 1.51-1.56 (m, 1H), 1.41-1.47 (m, 3H), 1.31-1.33 (m, 1H), 1.22 (dd, *J* = 12.5 Hz and 2.5 Hz, 1H), 1.12-1.18 (m, 1H); ^{13}C NMR (126 MHz, CD_3OD) δ 172.11, 65.57, 48.43, 47.19, 42.11, 40.43, 39.01, 38.83, 29.61, 24.79; ESI-HR-MS: (*m*/*z*) calcd. for $C_{10}H_{16}NO_2$ ([M+H]$^+$) 182.1176, found: 182.1176.

Compound **S4** (10 mg) was dissolved in CH_2Cl_2 (1 mL) followed by addition of *n*-hexane (3 mL). The resulting solution was filtered off and then left to evaporate at room temperature. Within 2-3 days, single-crystals suitable for single-crystal X-ray diffraction were obtained. A

crystal with dimension of 0.2 mm × 0.16 mm × 0.13 mm was selected for X-ray diffraction analysis using the method described above. The selected crystal data, structure refinement and geometric parameters are collected in Table S1. ORTEP drawing is shown in Figure S1.

2-((1*R**,2*S**,4*S**)-2-(Aminomethyl)bicyclo[2.2.1]heptan-2-yl)acetic acid (**WQQ-345**) hydrobromide. Following the procedure for the synthesis of **5**•HBr from **44**, **WQQ-345**•HBr was prepared from **S4** (21.00 g, 0.116 mol) in refluxing 40% HBr (150 mL). White solid, 13.90 g (45%). m.p. 162.2°C-166.0°C. ^{1}H NMR (500 MHz, CD_3OD) δ 3.17 (d, *J* = 13.5 Hz, 1H), 2.92 (d, *J* = 13.5 Hz, 1H), 2.67 (d, *J* = 16.5 Hz, 1H), 2.54 (d, *J* = 16.5 Hz, 1H), 2.29-2.30 (m, 2H), 1.60-1.67 (m, 3H), 1.46-1.53 (m, 2H), 1.33-1.36 (m, 1H), 1.19-1.25 (m, 1H), 1.10 (dd, *J* = 12.8 Hz and 2.8 Hz, 1H); ^{13}C NMR (126 MHz, CD_3OD) δ 176.34, 47.79, 43.95, 43.77, 42.82, 40.59, 38.60, 38.18, 28.96, 25.50; ESI-HR-MS: (m/z) calcd. for $C_{10}H_{19}NO_2$ ($[M+H]^+$) 184.1332, found: 184.1333.

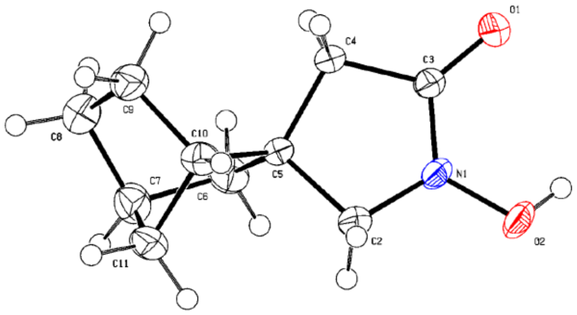

Figure S1. ORTEP drawing of compound **S4** (thermal ellipsoids both set at 50% probability. Carbon: grey; Nitrogen: blue; Oxygen: red)

*1.3 X-ray data of compound **44** and compound **68***

Compound **44** (10 mg) was dissolved in CH_2Cl_2 (1 mL) followed by addition of *n*-hexane (3 mL).The solution was filtered off and then left to evaporate at room temperature. Within 2-3 days, single-crystals suitable for single-crystal X-ray diffraction were obtained. A crystal with dimensions of 0.2 mm × 0.16 mm × 0.14 mm was selected for X-ray diffraction using the method described above. The selected crystal data, structure refinement and geometric parameters are collected in Table S1. ORTEP drawing is shown in Figure S2.

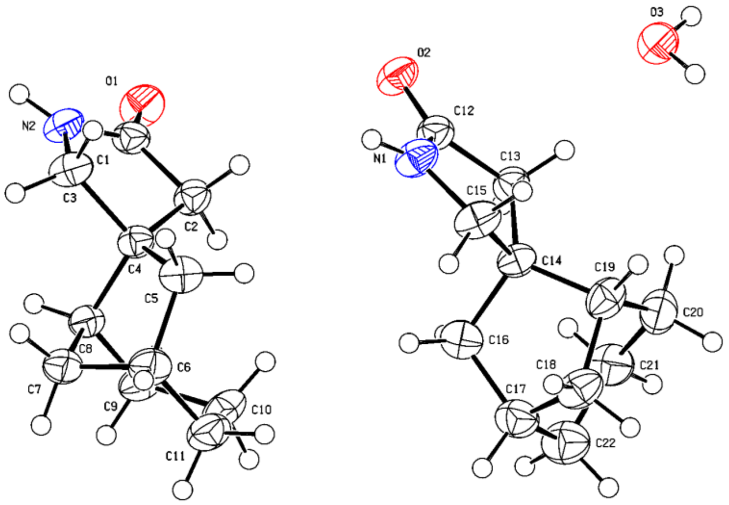

Figure S2. ORTEP drawing of compound **44** (thermal ellipsoids both set at 50% probability. Carbon: grey; Nitrogen: blue; Oxygen: red)

Compound **68** (10 mg) was dissolved in CH_2Cl_2 (1 mL) followed by addition of *n*-hexane (3 mL). The solution was filtered off and left to evaporate at room temperature. Within 2-3 days, single-crystals suitable for single-crystal X-ray diffraction were obtained. A crystal with dimensions of 0.17 mm × 0.14 mm × 0.08 mm was selected for X-ray diffraction using the method described above. The selected crystal data, structure refinement and geometric parameters are collected in Table S1. ORTEP drawing is shown in Figure S3.

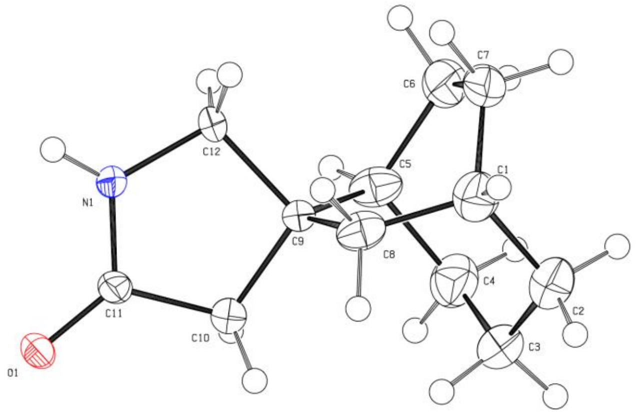

Figure S3. ORTEP drawing of compound **68** (thermal ellipsoids both set at 50% probability. Carbon: grey; Nitrogen: blue; Oxygen: red)

Table S1. Selected crystal data and structure for compound **44**, **68** and **S4.**

Compound	44	68	S4
CCDC	2335868	2335869	2335866
Formula	$2(C_{11}H_{17}NO)•H_2O$	$C_{12}H_{19}NO$	$C_{10}H_{15}NO_2$
$M/g·mol^{-1}$	376.53	193.28	181.23
Crystal system	Triclinic	Monoclinic	Monoclinic
Space group	*P*-1	$P2_1/C$	$P2_1/C$
Temperature (K)	150.00	100.00	150.00
a, *b*, *c* (Å)	6.5349(6), 10.9369(8), 15.6423(13)	12.6013(17), 7.3390(10), 11.8098(16)	12.1891(4), 6.3594(2), 13.1183(5)
α, *β*, *γ* (°)	108.654(4), 100.381(3), 93.236(3)	90, 106.557(5), 90	90, 116.2370(10), 90
V (Å^3)	1034.07(15)	1046.9(2)	912.10(5)
Z	2	4	4
ρ_c/g cm^{-3}	1.209	1.226	1.320
Radiation type	Mo *Kα*	Mo *Kα*	Mo *Kα*
F(000)	412	412	392
Crystal size (mm)	0.2×0.16×0.14	0.17×0.14×0.08	0.2×0.16×0.13
Absorption μ/mm^{-1}	0.080	0.077	0.092
Max. *T*	0.751	0.745	0.752
Min. *T*	0.603	0.407	0.638
Final *R* indices [$I > 2\sigma(I)$] R_1	0.0620	0.0901	0.0642
wR_2	0.1727	0.2752	0.1821
R indices (all data) R_1	0.1240	0.1108	0.0743
No. of reflections	4186	1977	2091
No. of parameters	244	200	119
$D\rho_{max}$, $D\rho_{min}$ (e Å^{-3})	0.601, -0.249	0.749, -0.752	0.603, -0.308

Reference

1. Sheldrick, G.M. Crystal structure refinement with SHELXL. *Acta Crystallogr. C* **2015**, *71*, 3–8. https://doi.org/10.1107/s2053229614024218.

2. Copies of ^{1}H NMR, ^{13}C NMR and HR-MS spectra

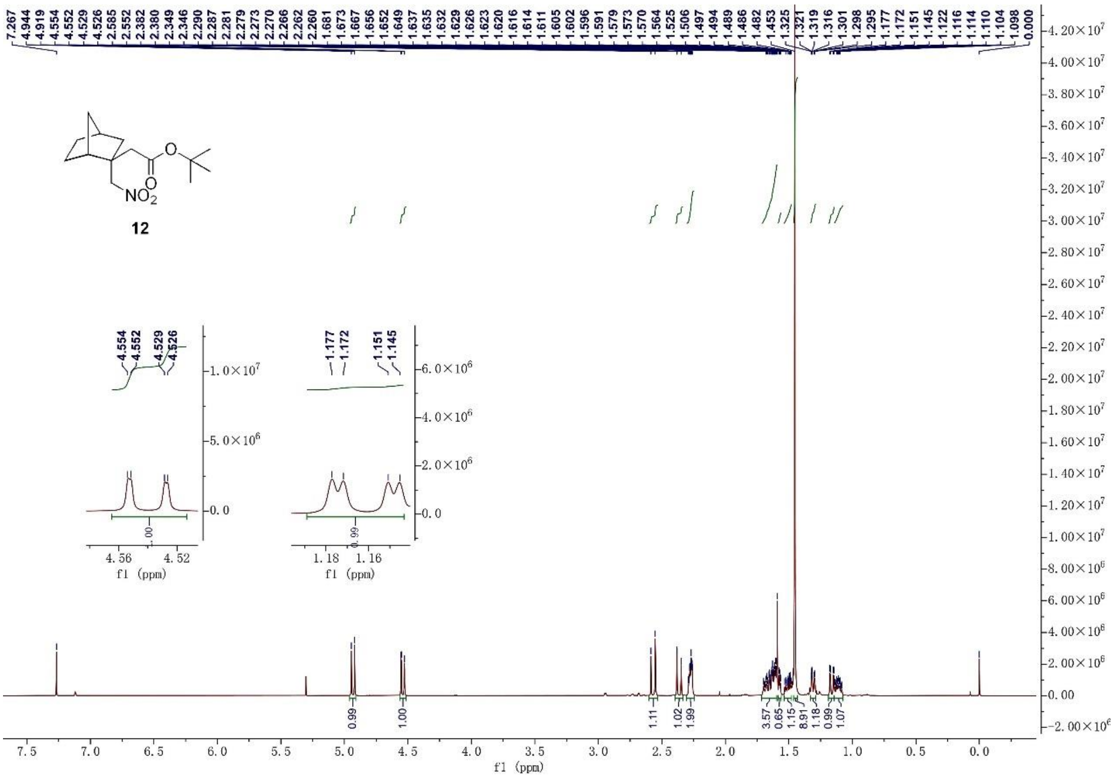

Figure S4. ^{1}H NMR spectrum of **12** (500 MHz, $CDCl_3$)

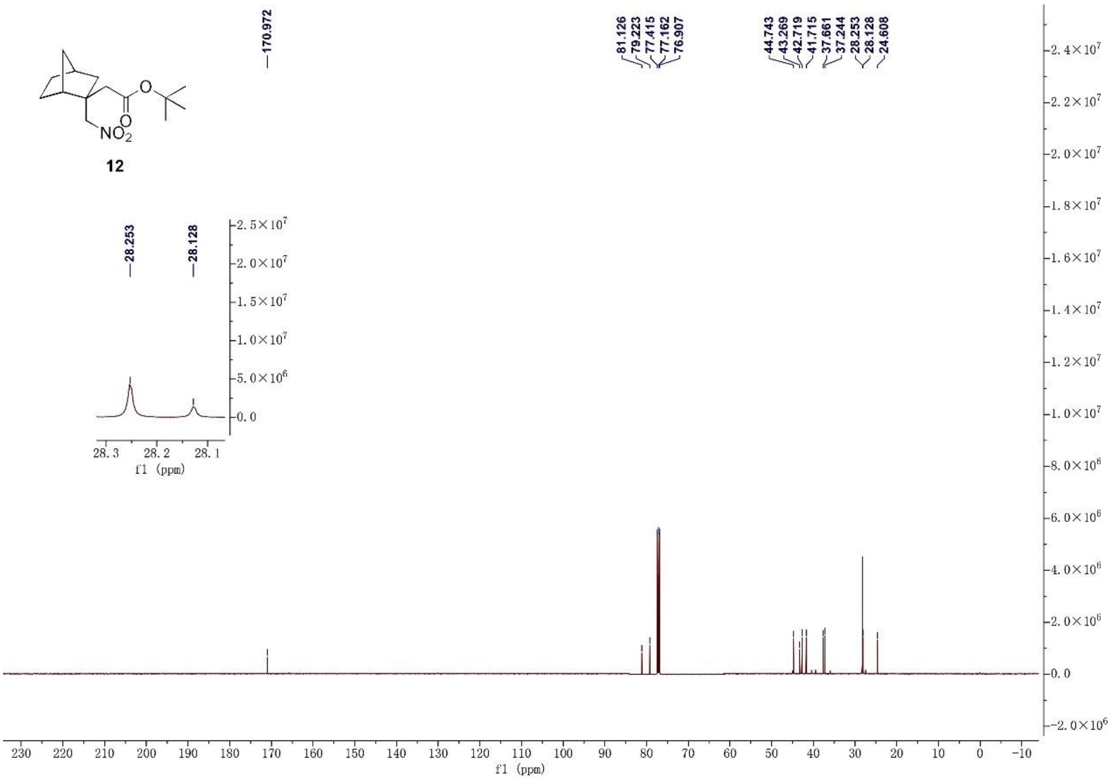

Figure S5. ^{13}C NMR spectrum of **12** (126 MHz, $CDCl_3$)

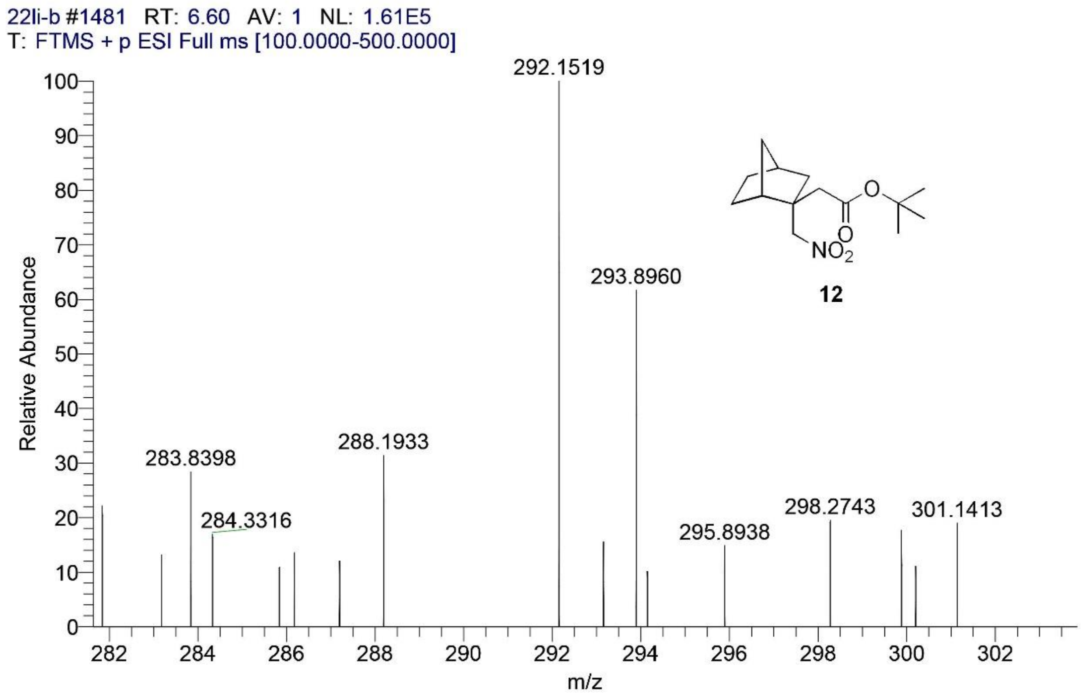

Figure S6. HR-MS spectrum of **12**

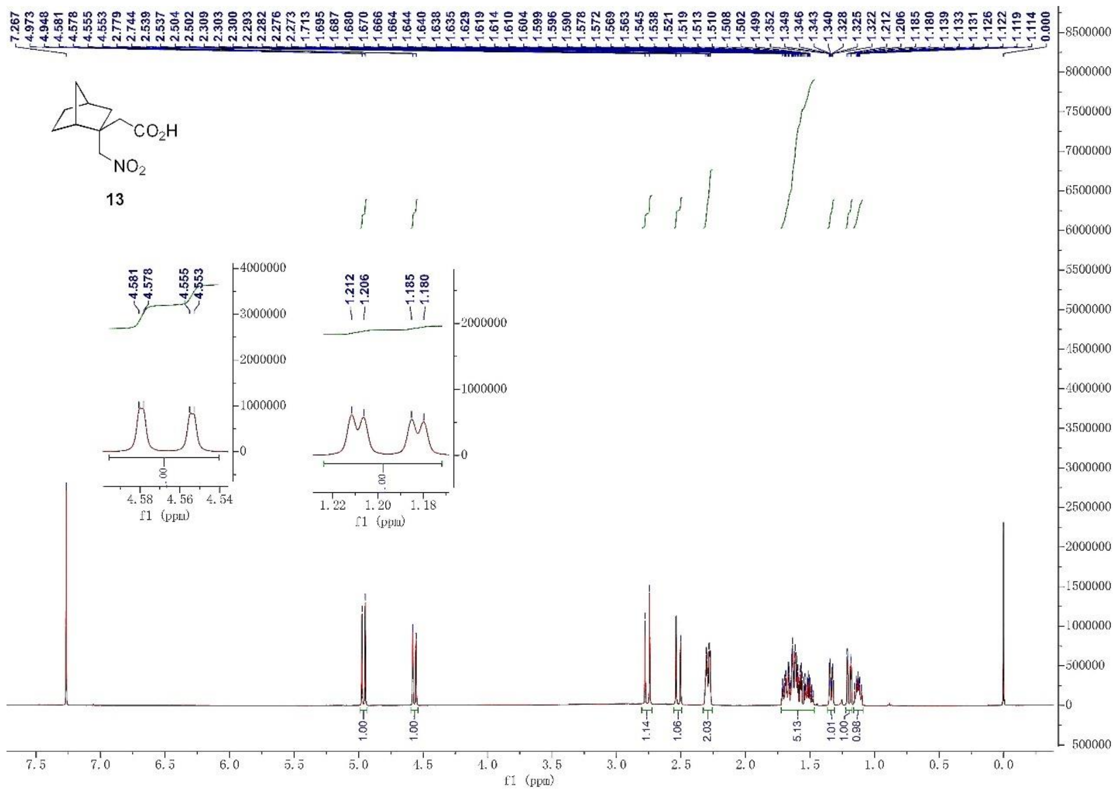

Figure S7. ^{1}H NMR spectrum of **13** (500 MHz, $CDCl_3$)

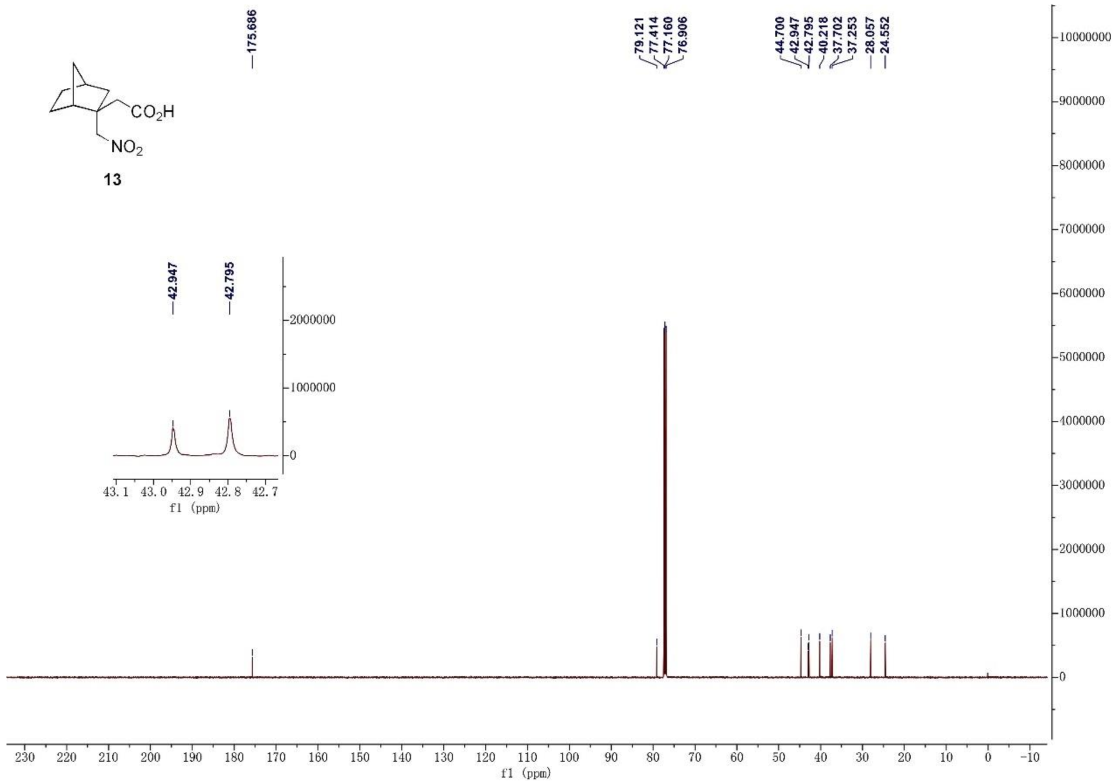

Figure S8. ^{13}C NMR spectrum of **13** (126 MHz, $CDCl_3$)

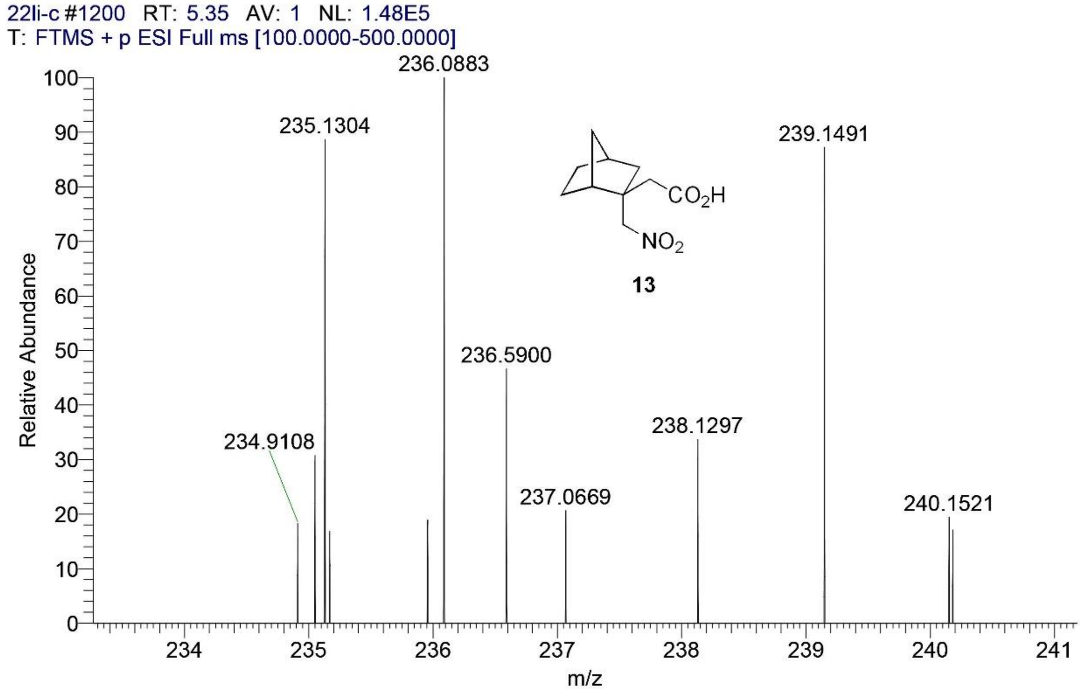

Figure S9. HR-MS spectrum of **13**

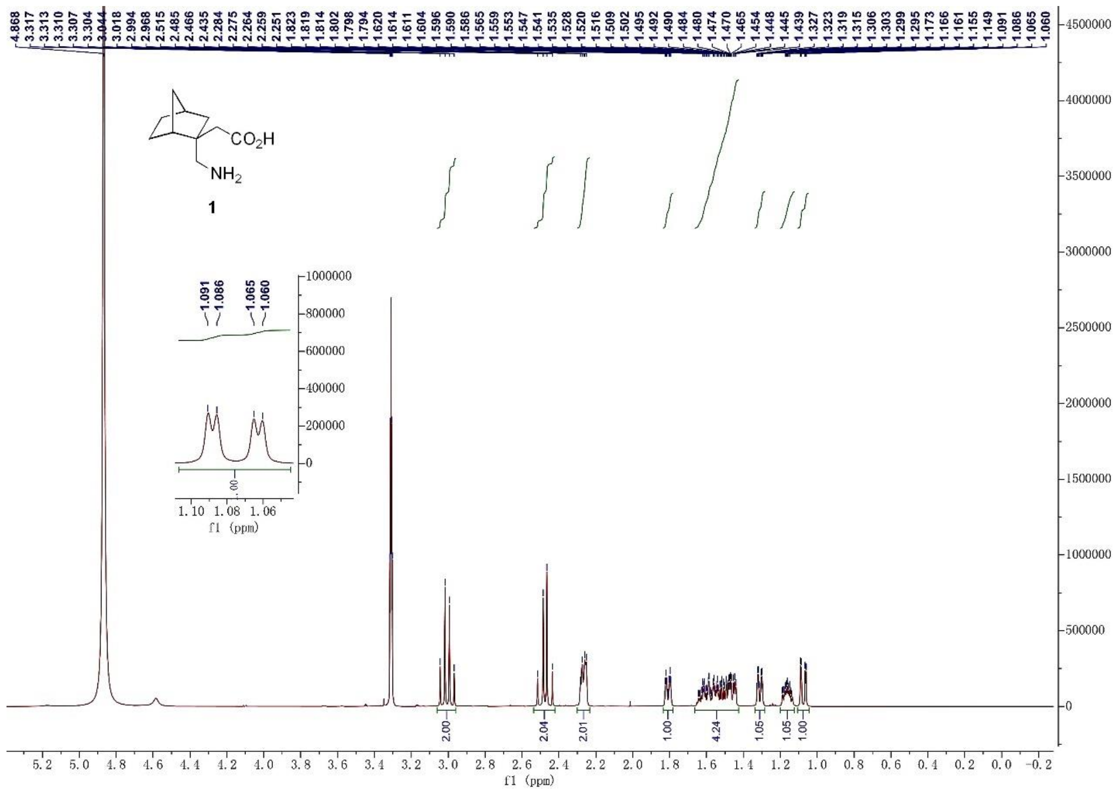

Figure S10. ^{1}H NMR spectrum of **1** (500 MHz, CD_3OD)

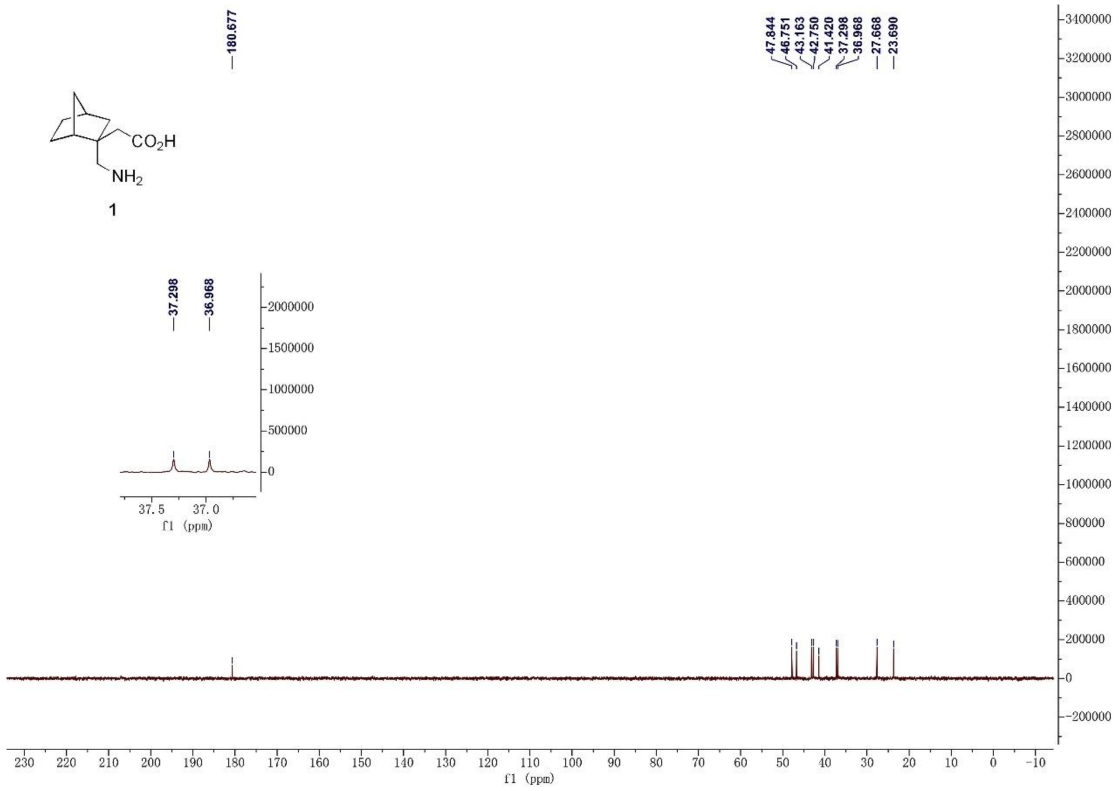

Figure S11. ^{13}C NMR spectrum of **1** (126 MHz, D_2O)

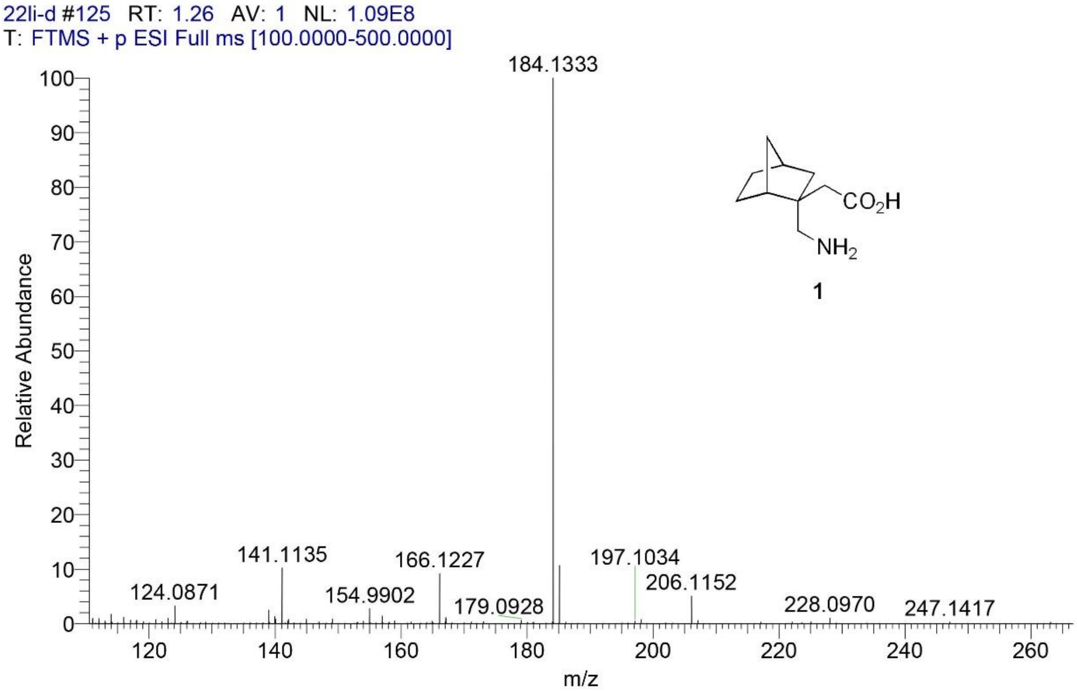

Figure S12. HR-MS spectrum of **1**

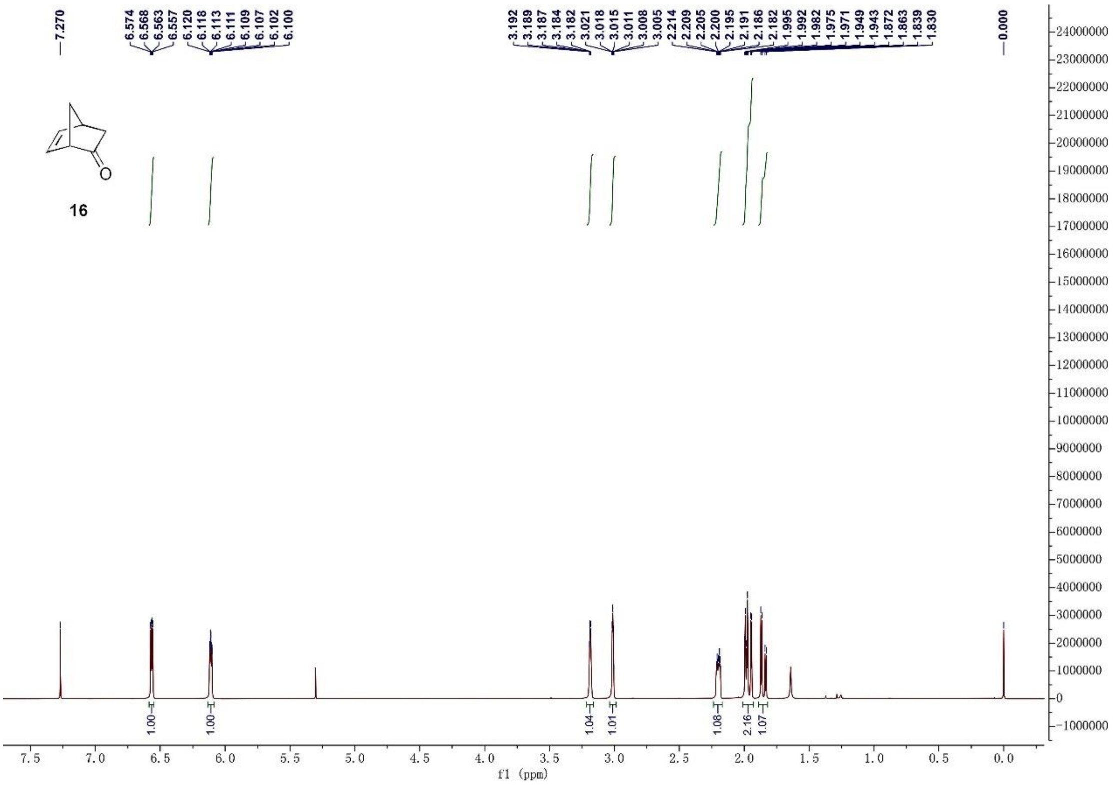

Figure S13. ^{1}H NMR spectrum of **16** (500 MHz, $CDCl_3$)

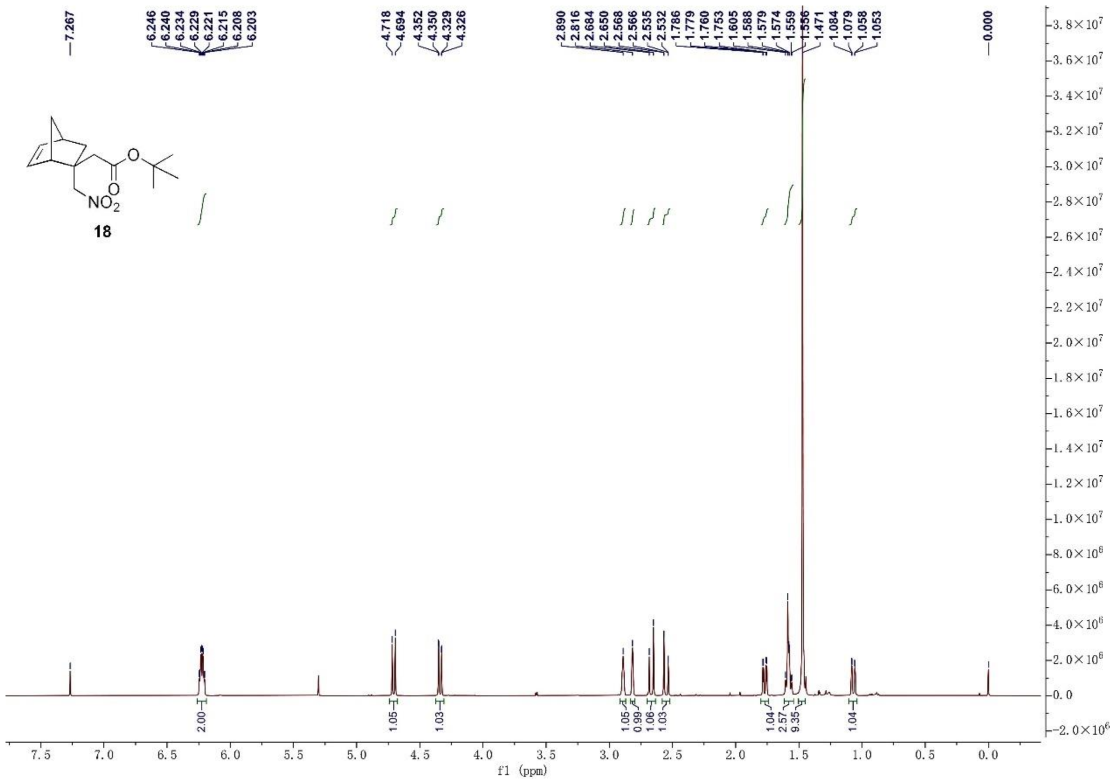

Figure S14. ^{1}H NMR spectrum of **18** (500 MHz, $CDCl_3$)

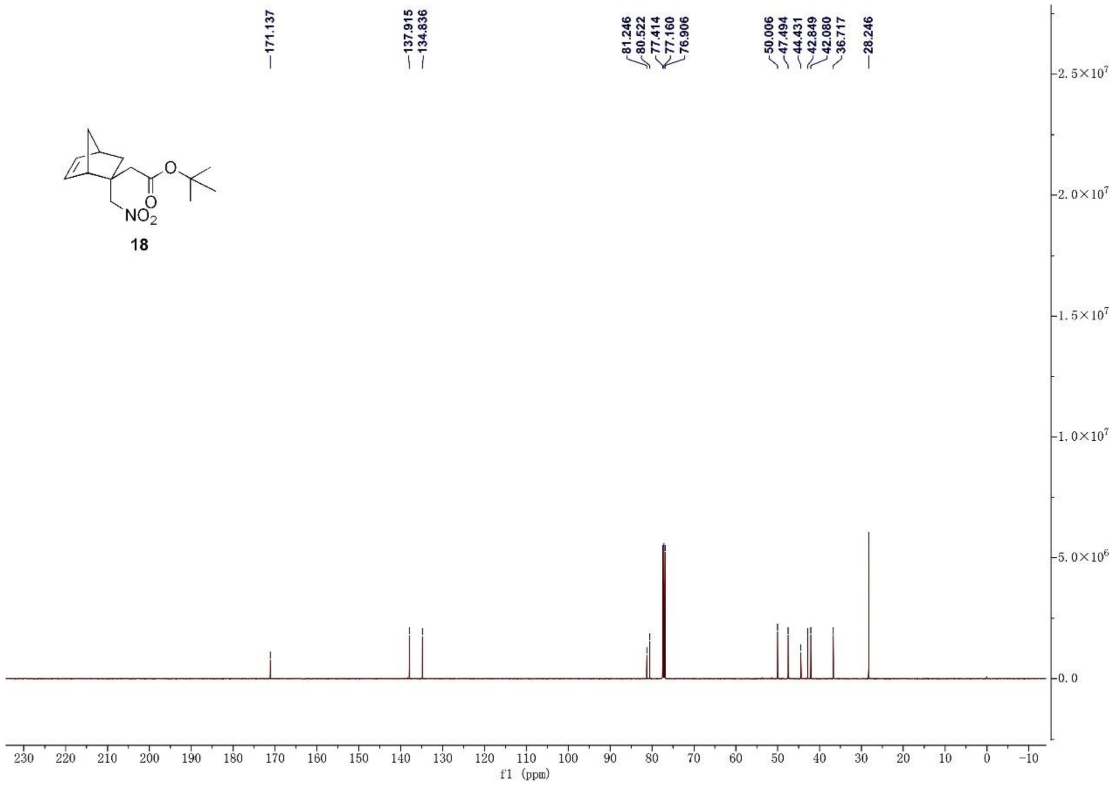

Figure S15. ^{13}C NMR spectrum of **18** (126 MHz, $CDCl_3$)

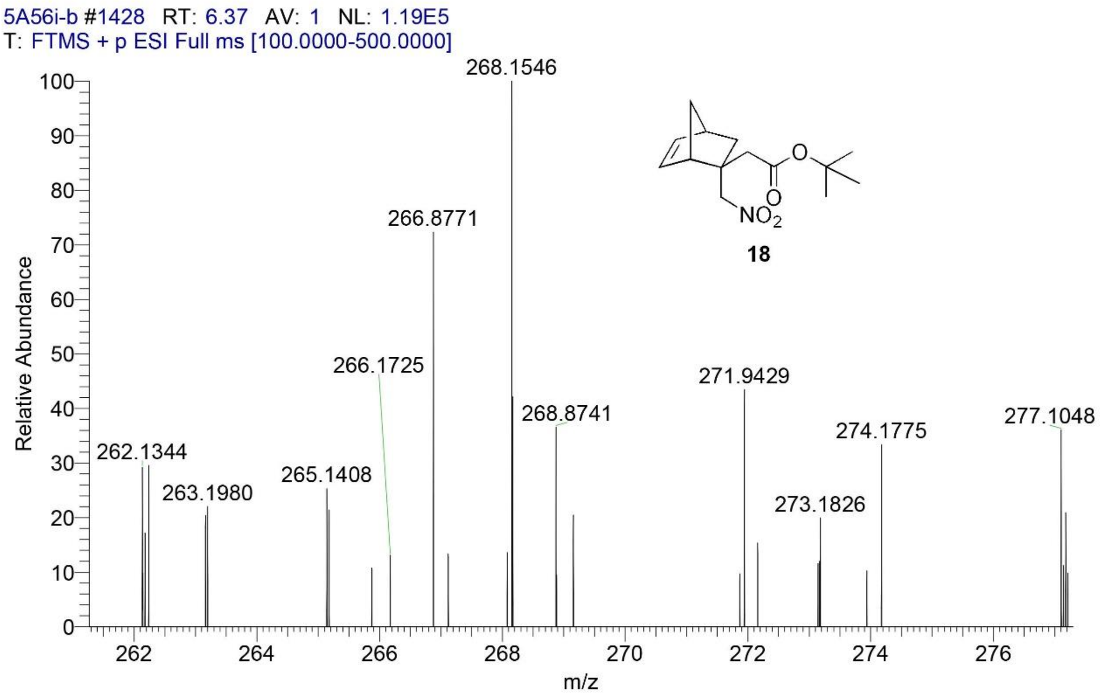

Figure S16. HR-MS spectrum of **18**

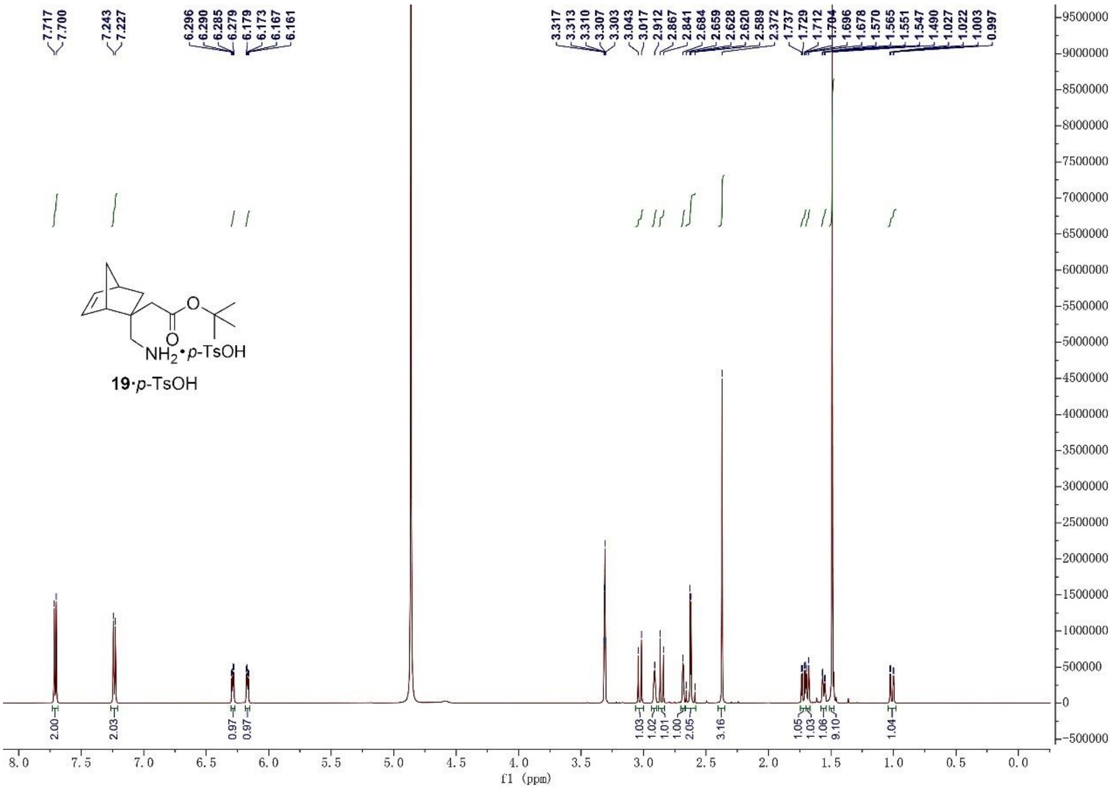

Figure S17. ^{1}H NMR spectrum of **19•**p-TsOH (500 MHz, CD_3OD)

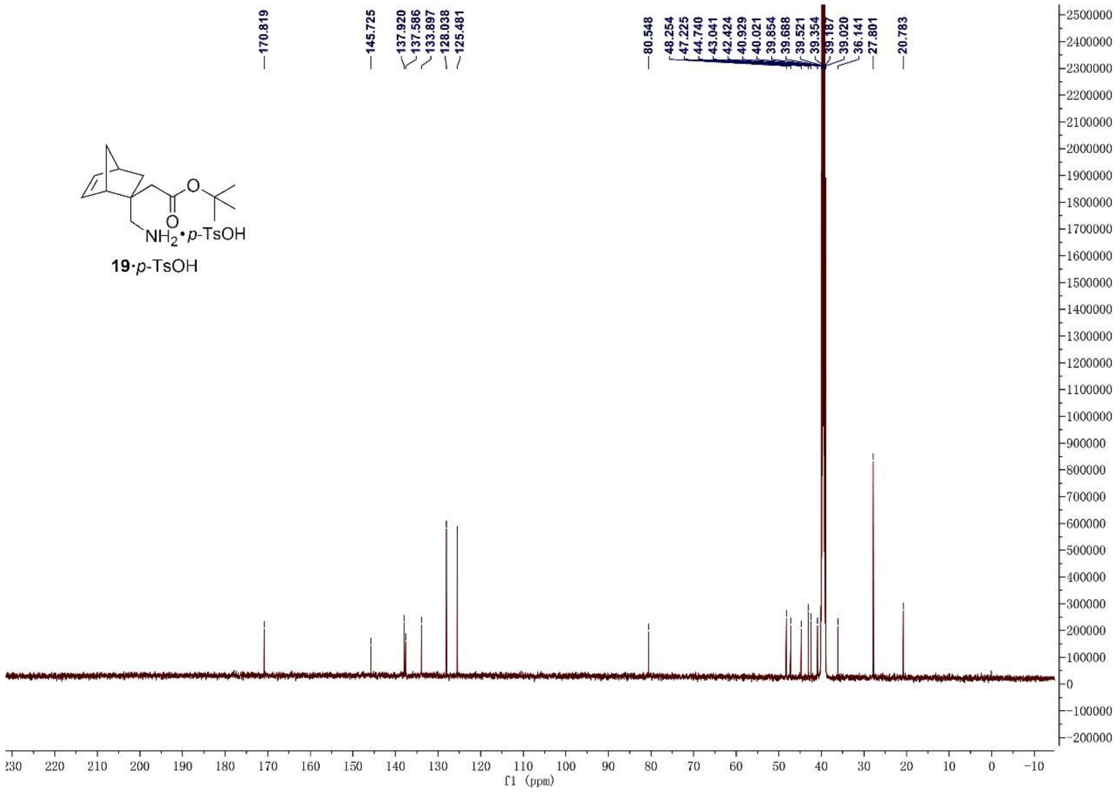

Figure S18. ^{13}C NMR spectrum of **19•***p*-TsOH (126 MHz, CD_3OD)

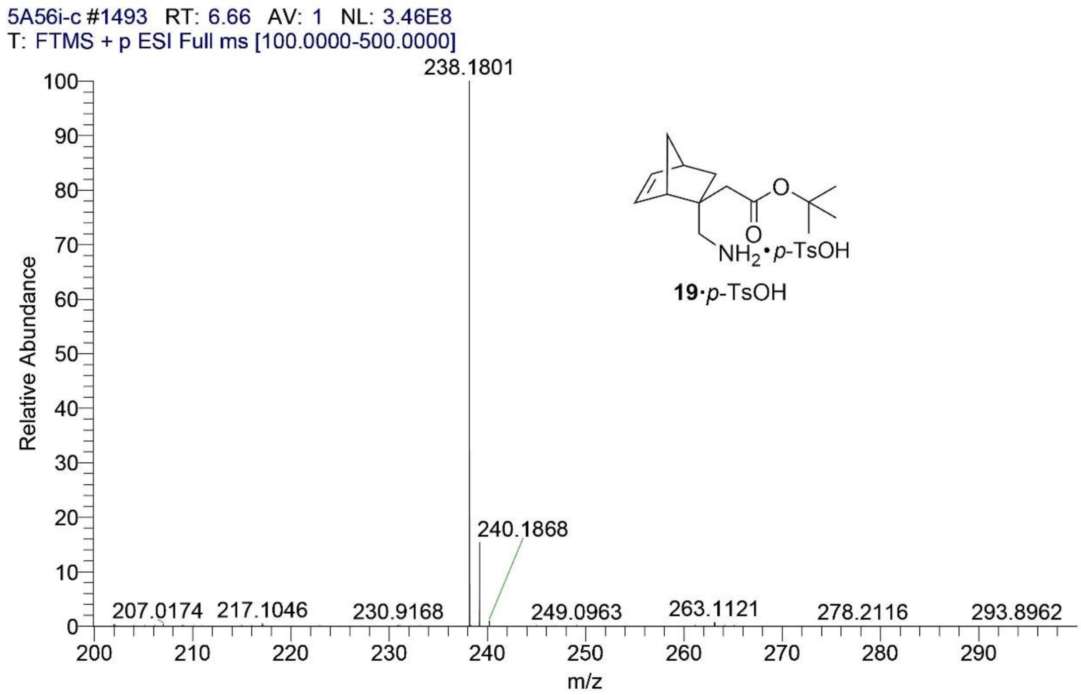

Figure S19. HR-MS spectrum of **19•***p*-TsOH

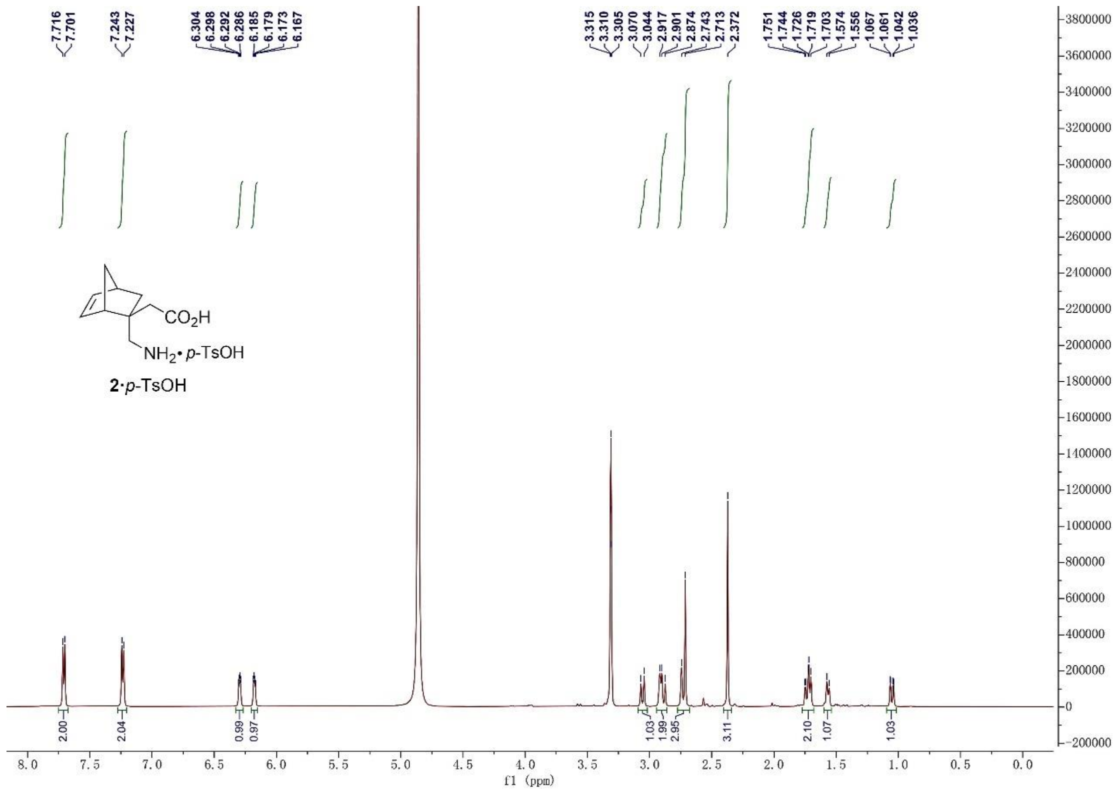

Figure S20. ^{1}H NMR spectrum of **2**•*p*-TsOH (500 MHz, CD_3OD)

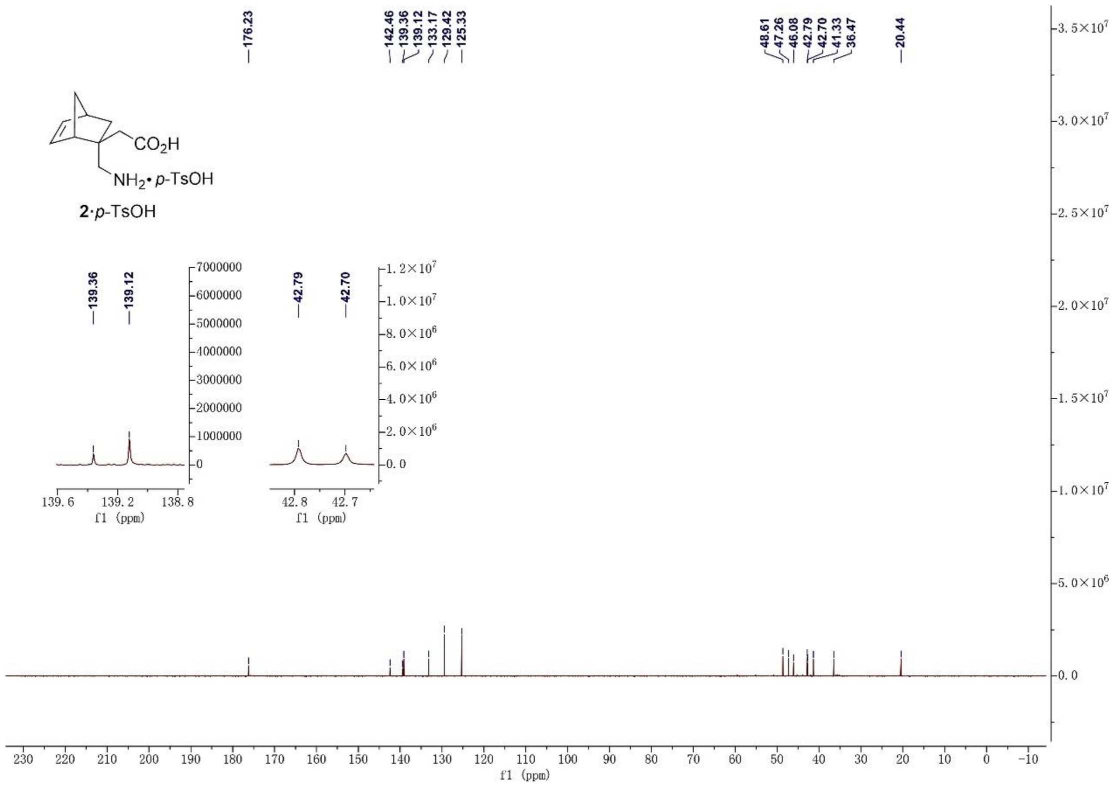

Figure S21. ^{13}C NMR spectrum of **2**•*p*-TsOH (126 MHz, D_2O)

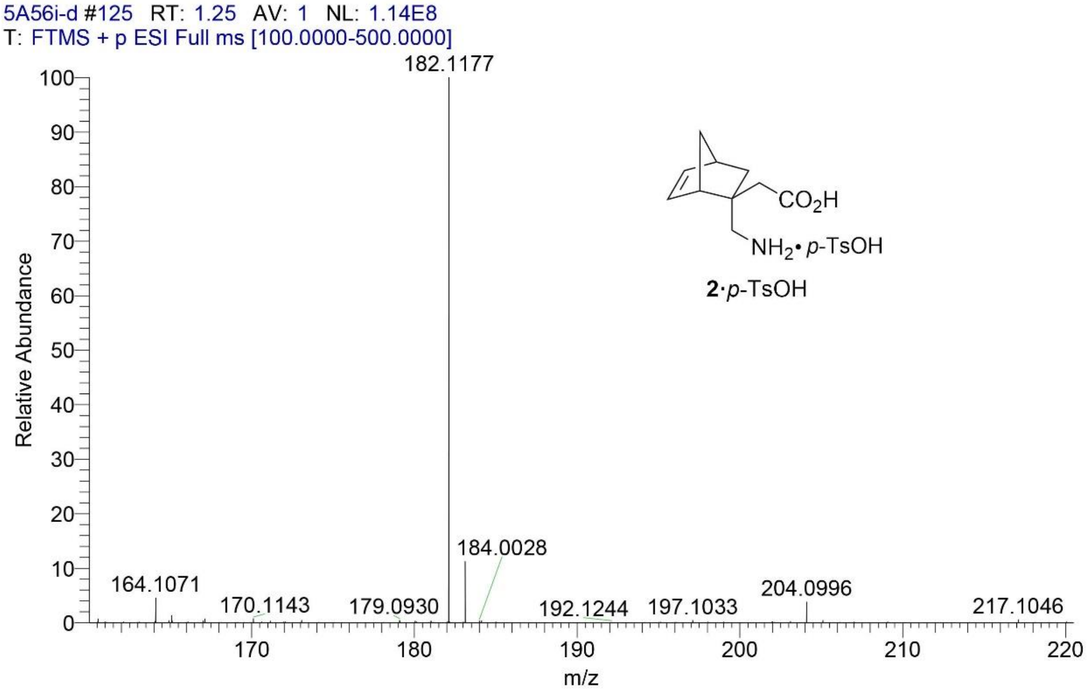

Figure S22. HR-MS spectrum of **2•***p*-TsOH

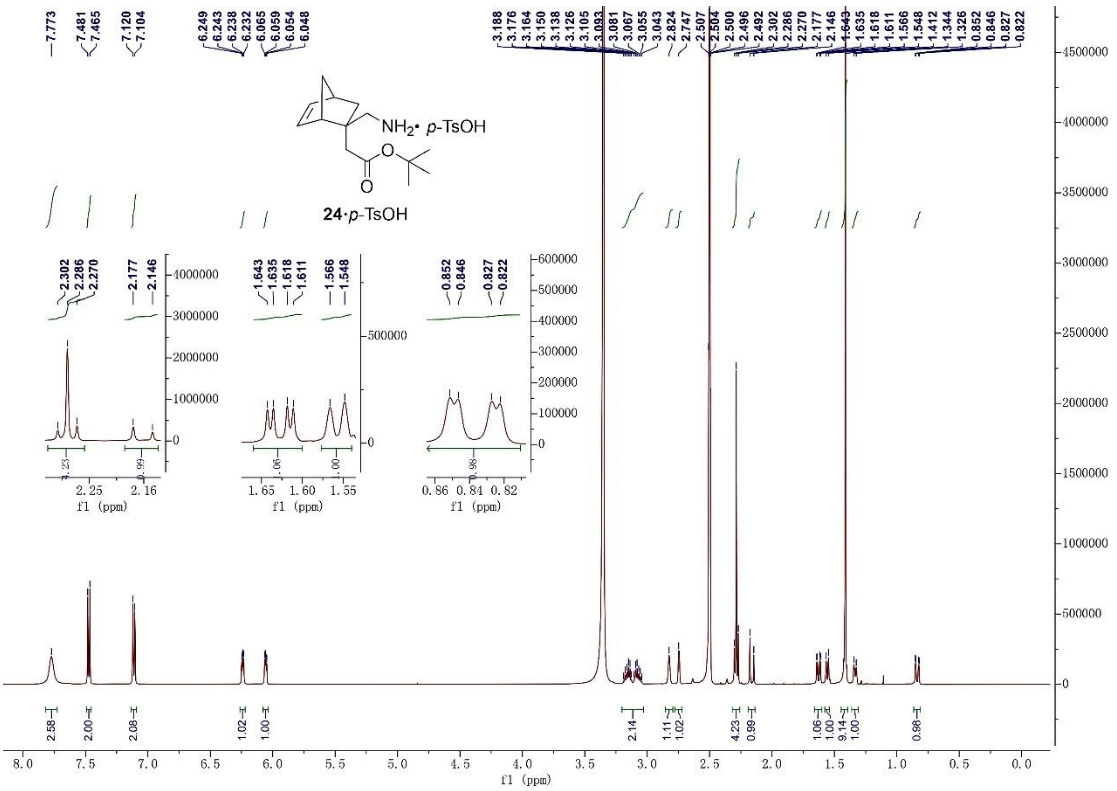

Figure S23. ^{1}H NMR spectrum of **24•***p*-TsOH (500 MHz, DMSO)

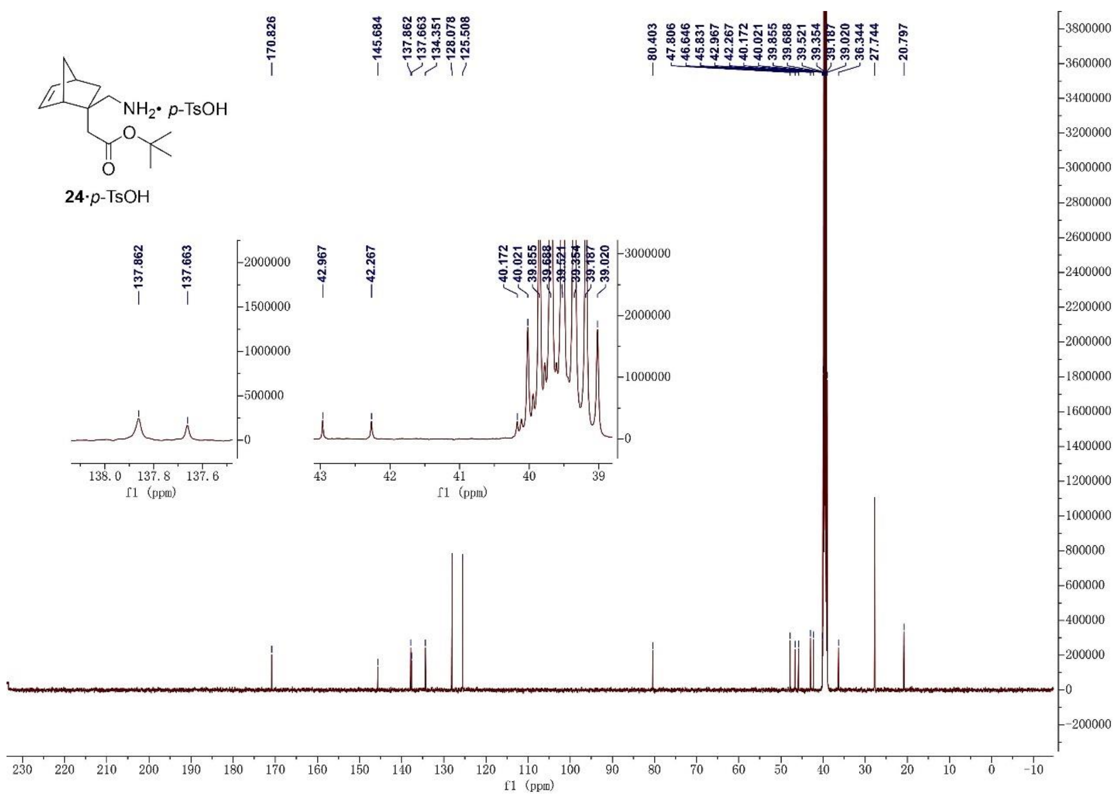

Figure S24. ^{13}C NMR spectrum of **24•***p*-TsOH (126 MHz, DMSO)

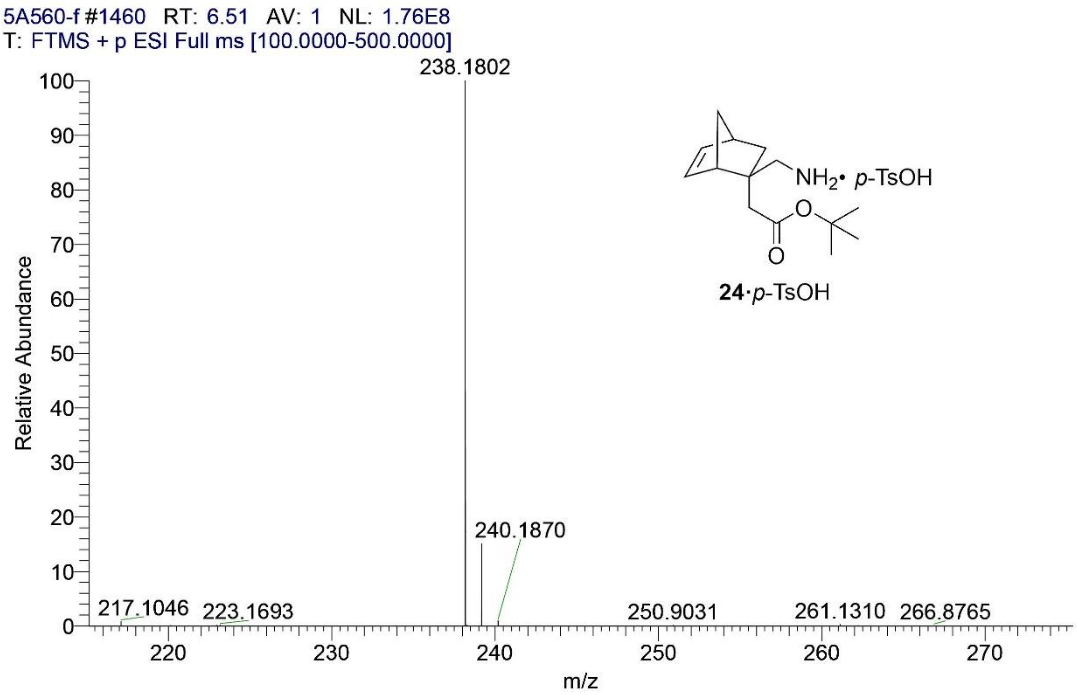

Figure S25. HR-MS spectrum of **24•***p*-TsOH

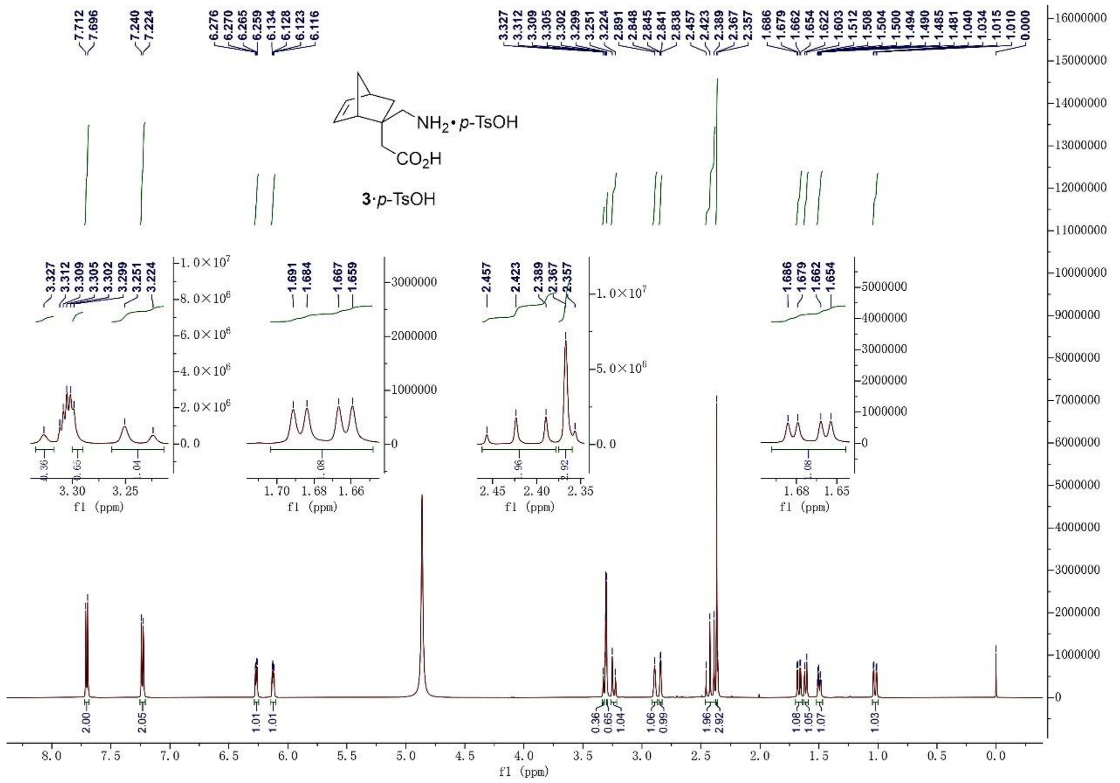

Figure S26. ^{1}H NMR spectrum of **3•**p-TsOH (500 MHz, CD_3OD)

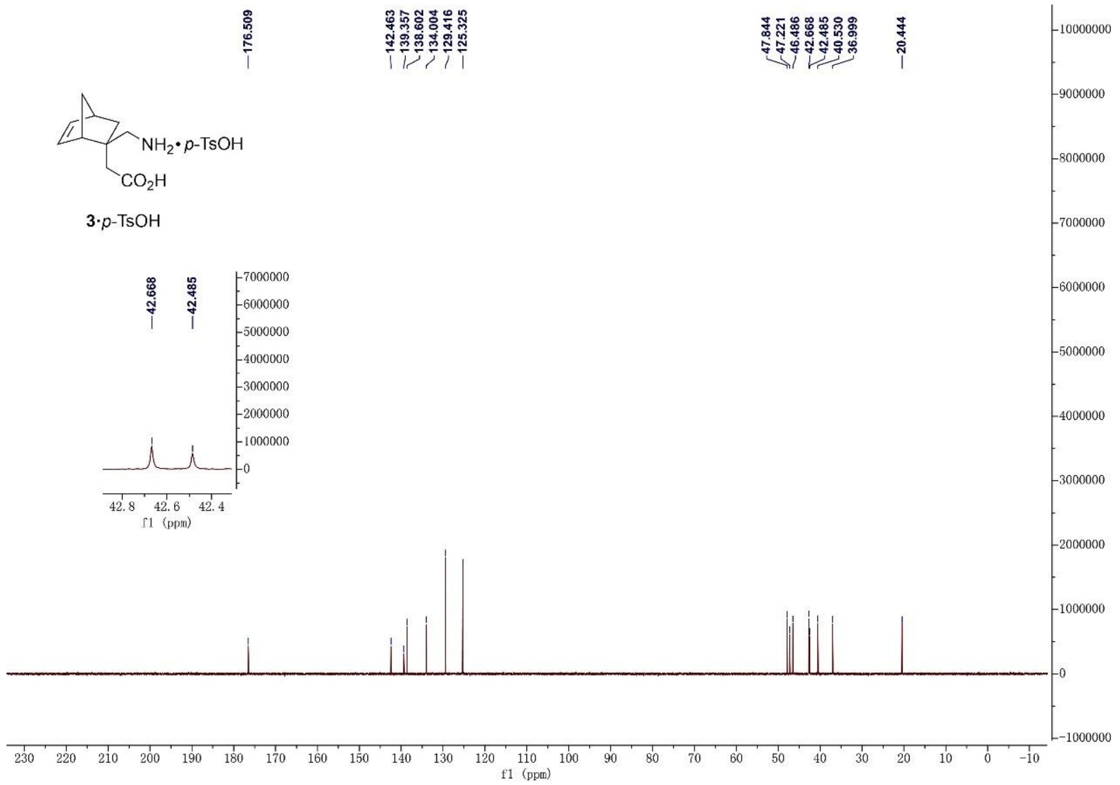

Figure S27. ^{13}C NMR spectrum of **3•**p-TsOH (126 MHz, D_2O)

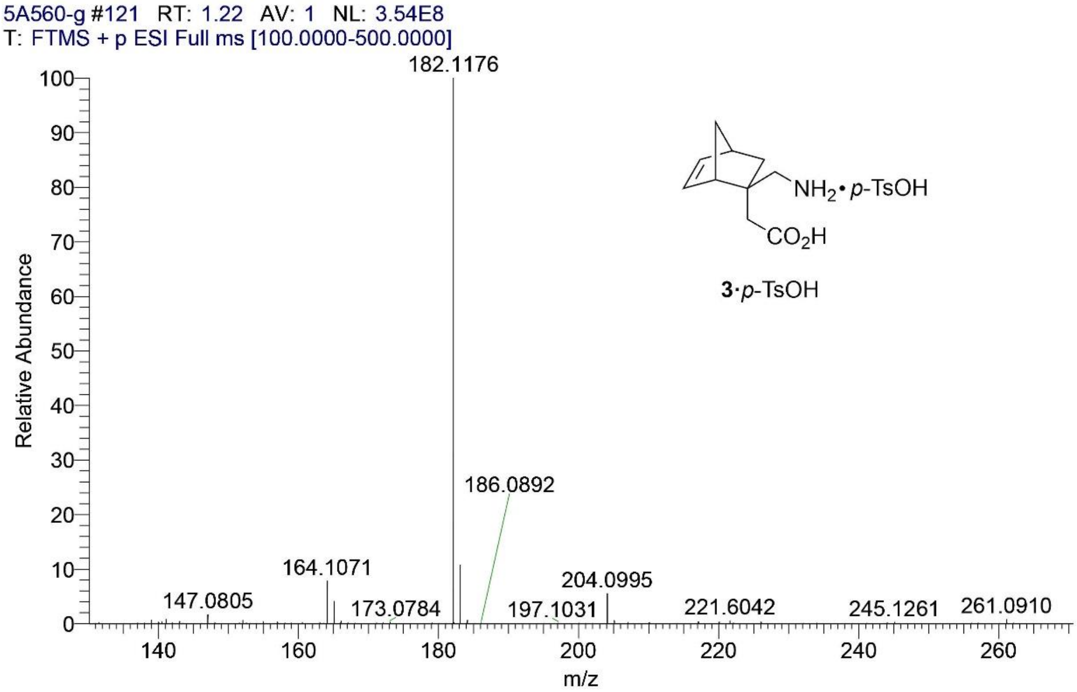

Figure S28. HR-MS spectrum of **3•***p*-TsOH

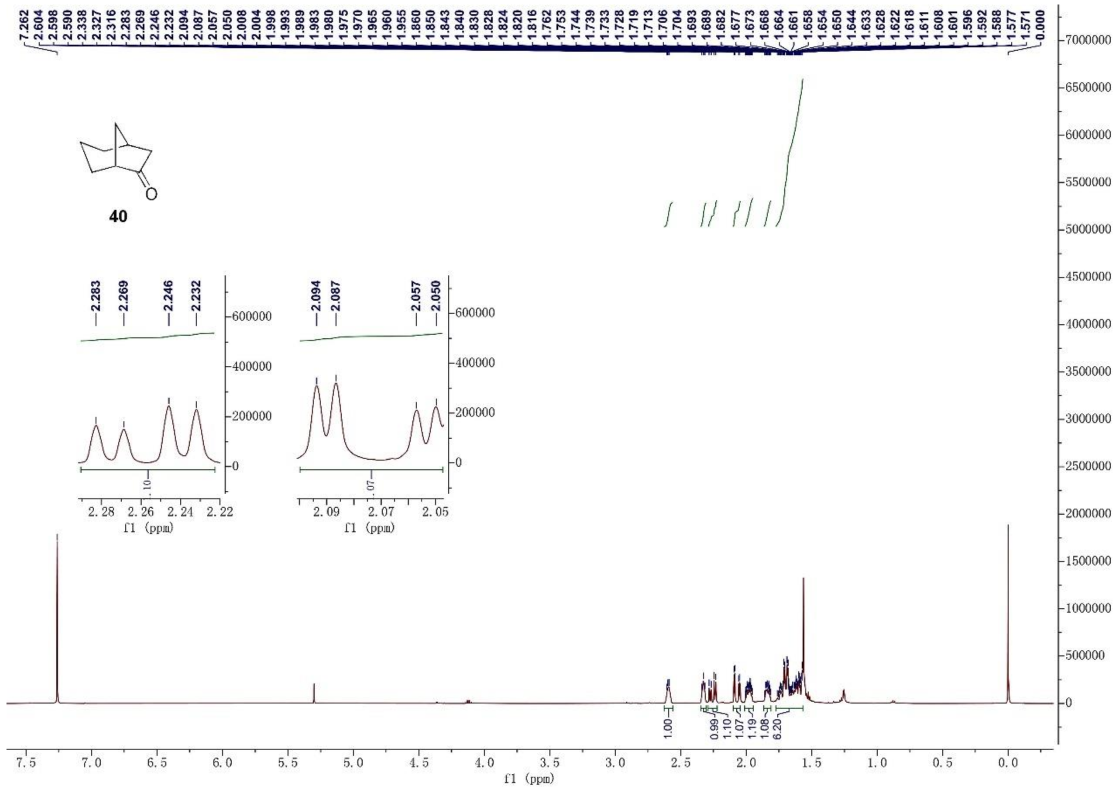

Figure S29. ^{1}H NMR spectrum of **40** (500 MHz, $CDCl_3$)

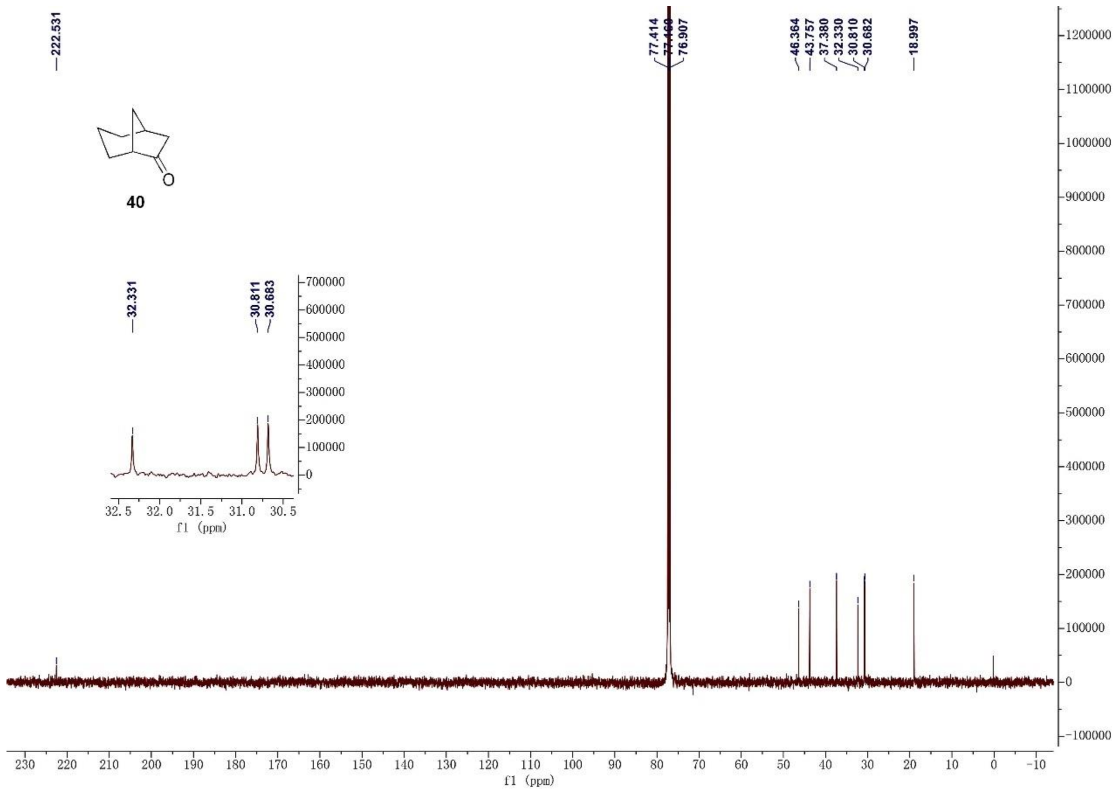

Figure S30. ^{13}C NMR spectrum of **40** (126 MHz, $CDCl_3$)

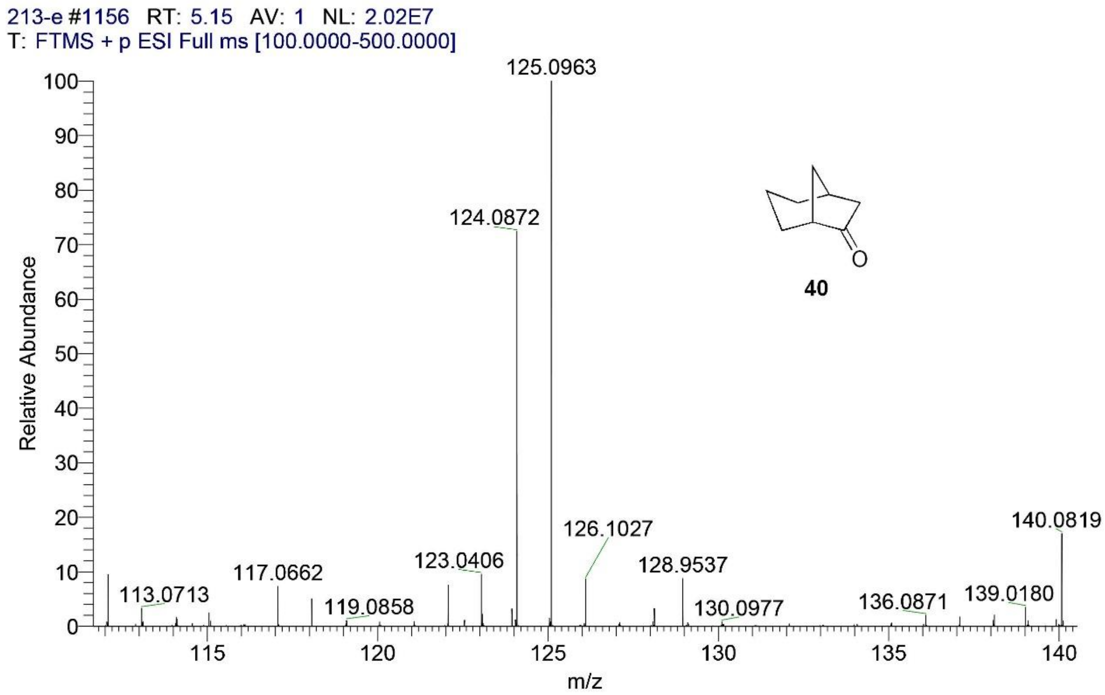

Figure S31. HR-MS spectrum of **40**

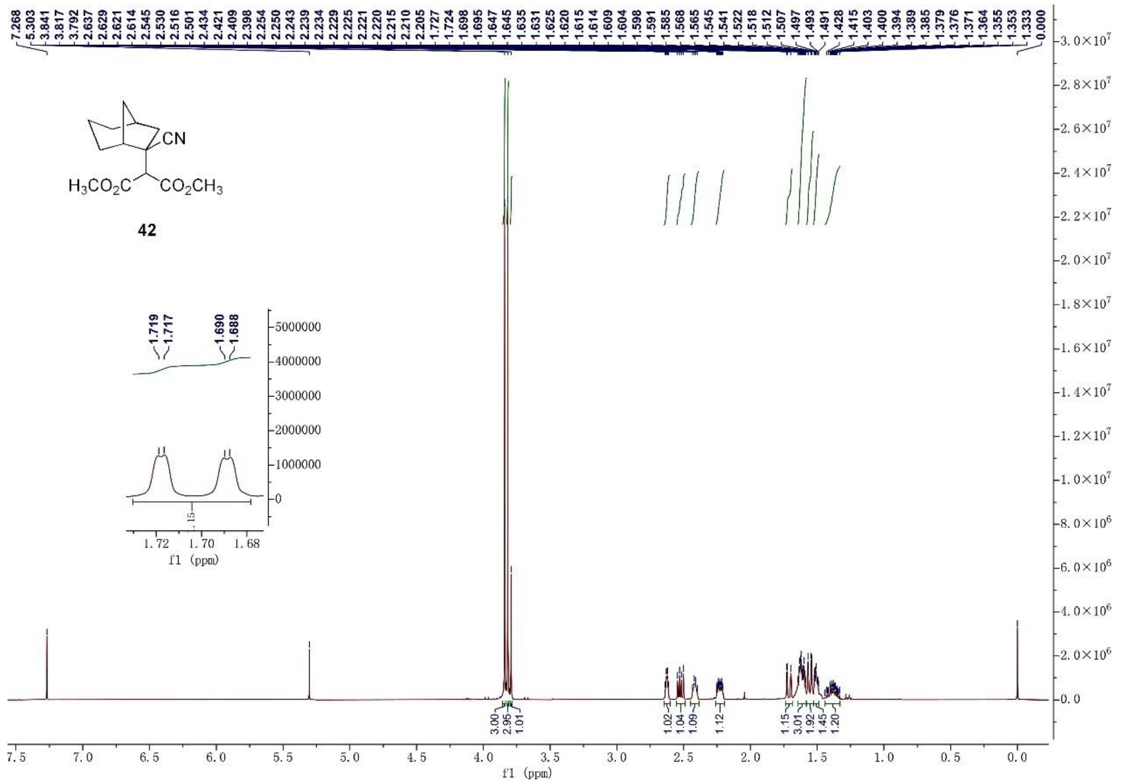

Figure S32. ^{1}H NMR spectrum of **42** (500 MHz, $CDCl_3$)

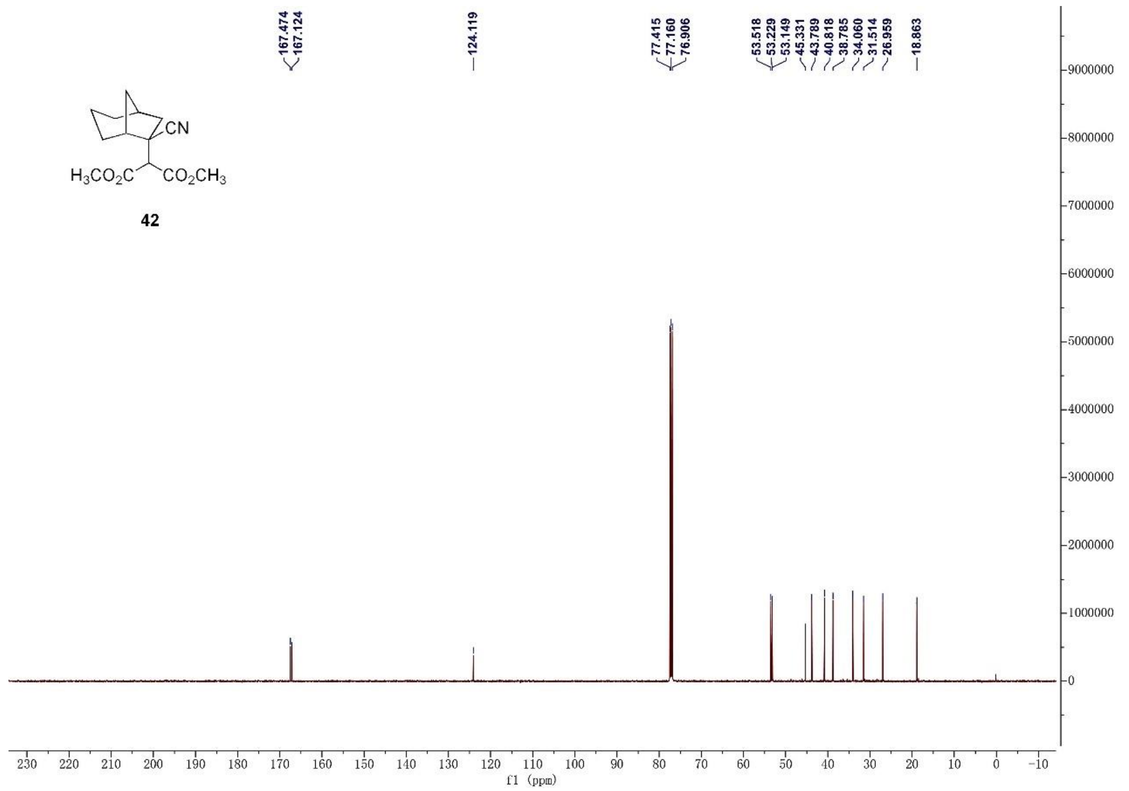

Figure S33. ^{13}C NMR spectrum of **42** (126 MHz, $CDCl_3$)

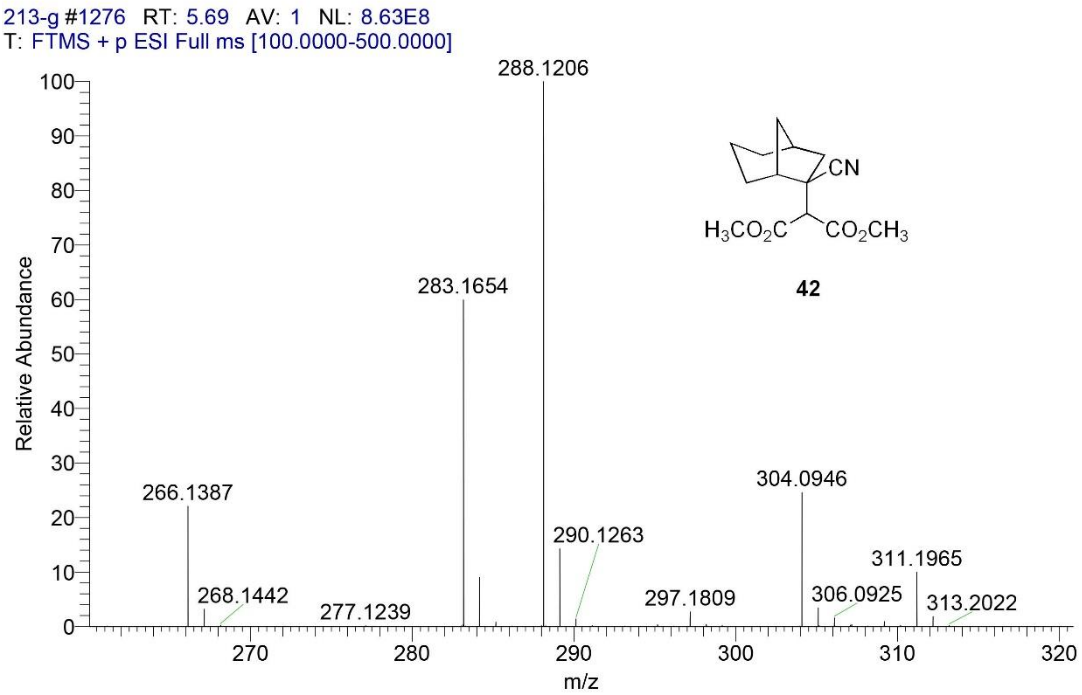

Figure S34. HR-MS spectrum of **42**

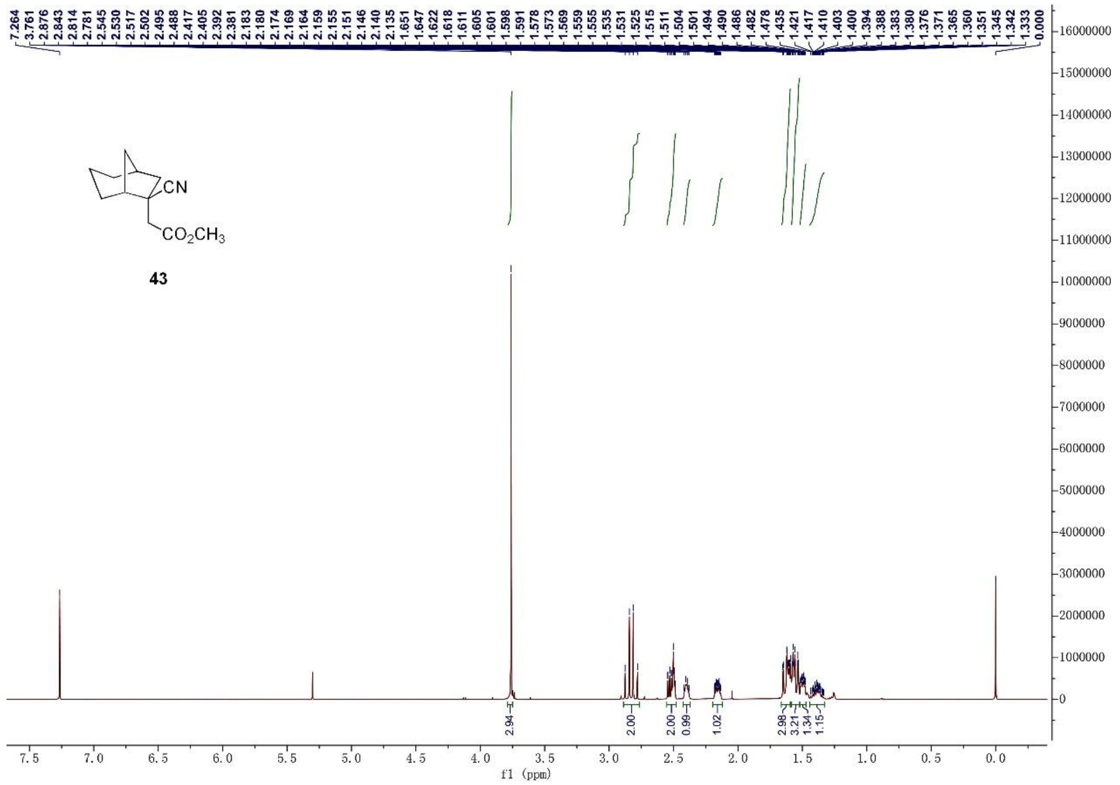

Figure S35. ^{1}H NMR spectrum of **43** (500 MHz, $CDCl_3$)

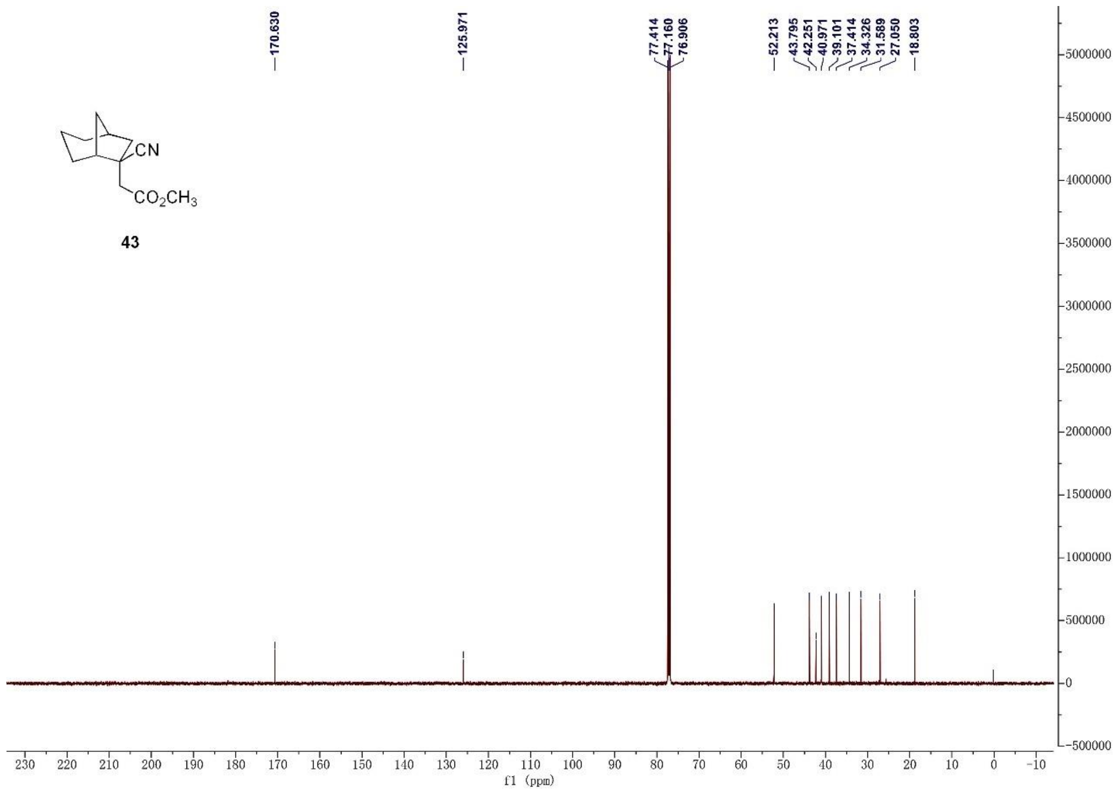

Figure S36. ^{13}C NMR spectrum of **43** (126 MHz, $CDCl_3$)

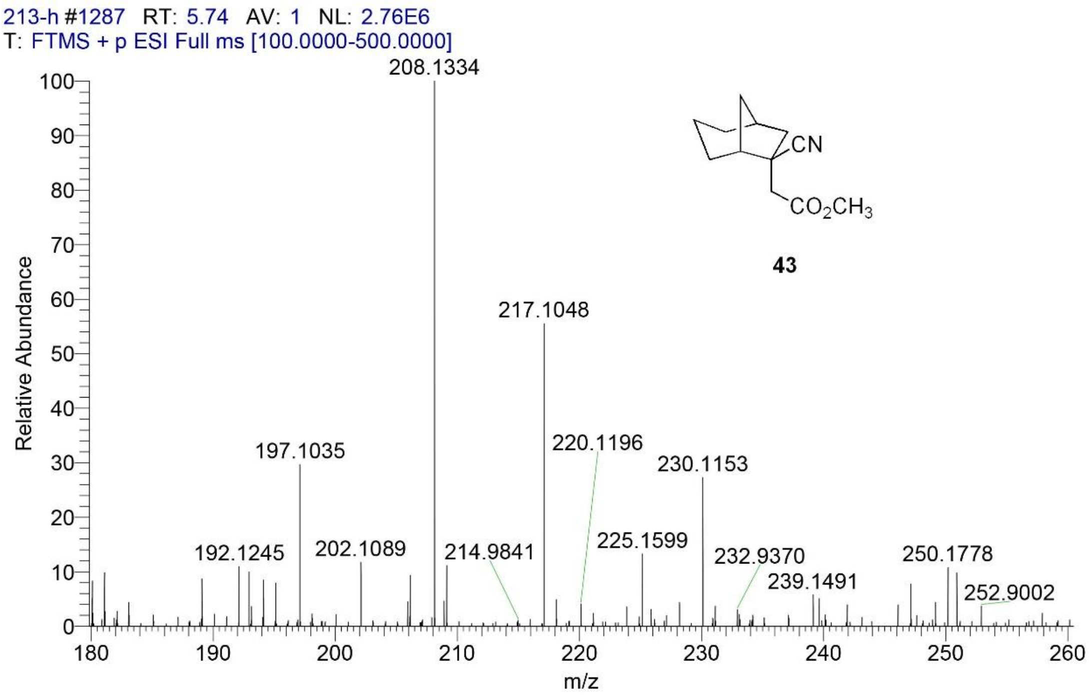

Figure S37. HR-MS spectrum of **43**

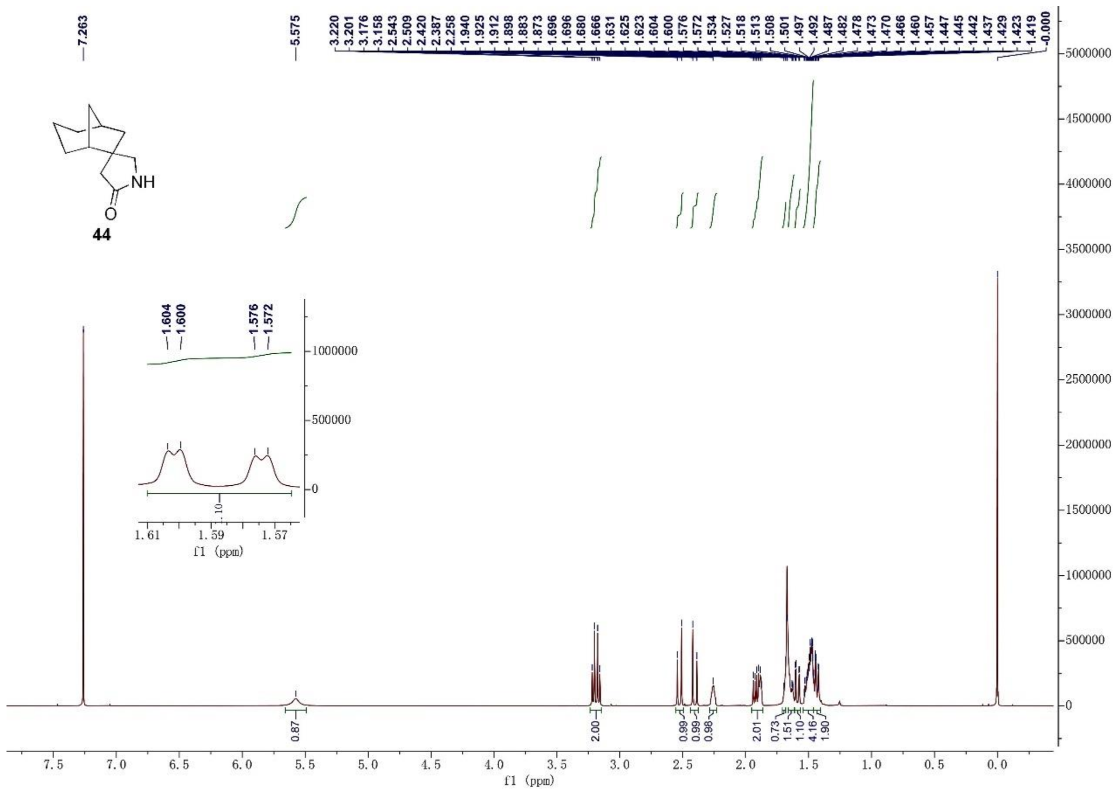

Figure S38. ^{1}H NMR spectrum of **44** (500 MHz, $CDCl_3$)

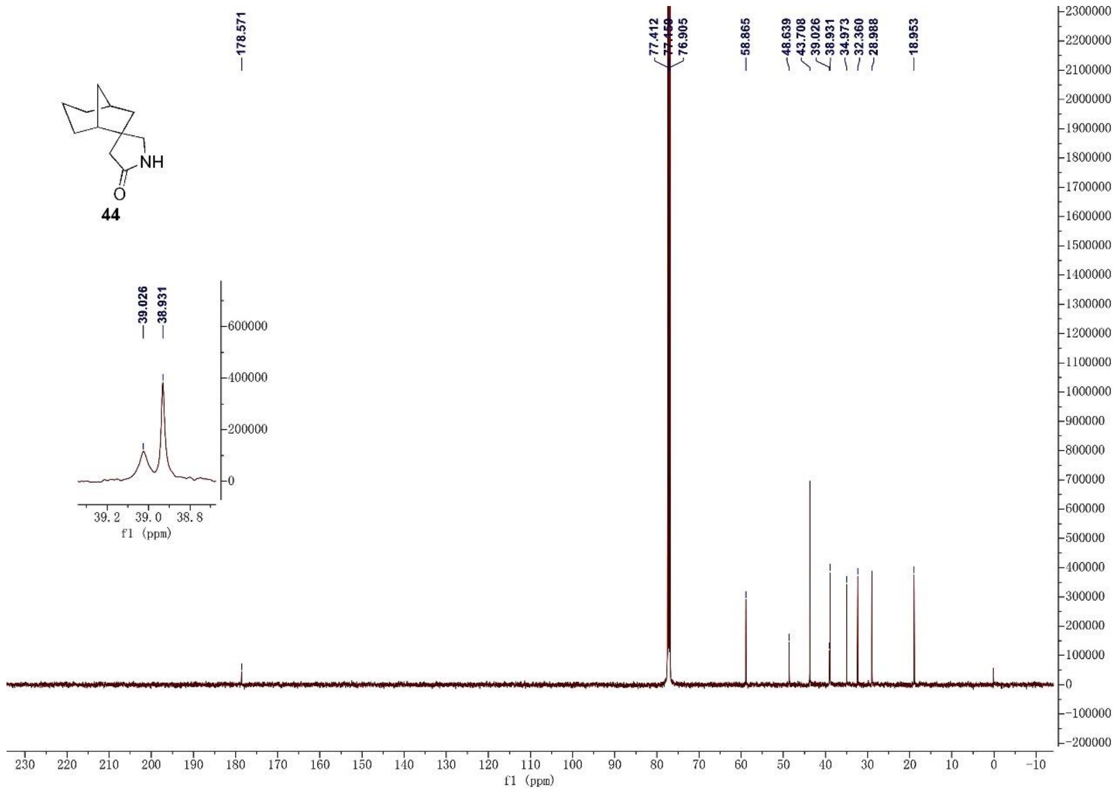

Figure S39. ^{13}C NMR spectrum of **44** (126 MHz, $CDCl_3$)

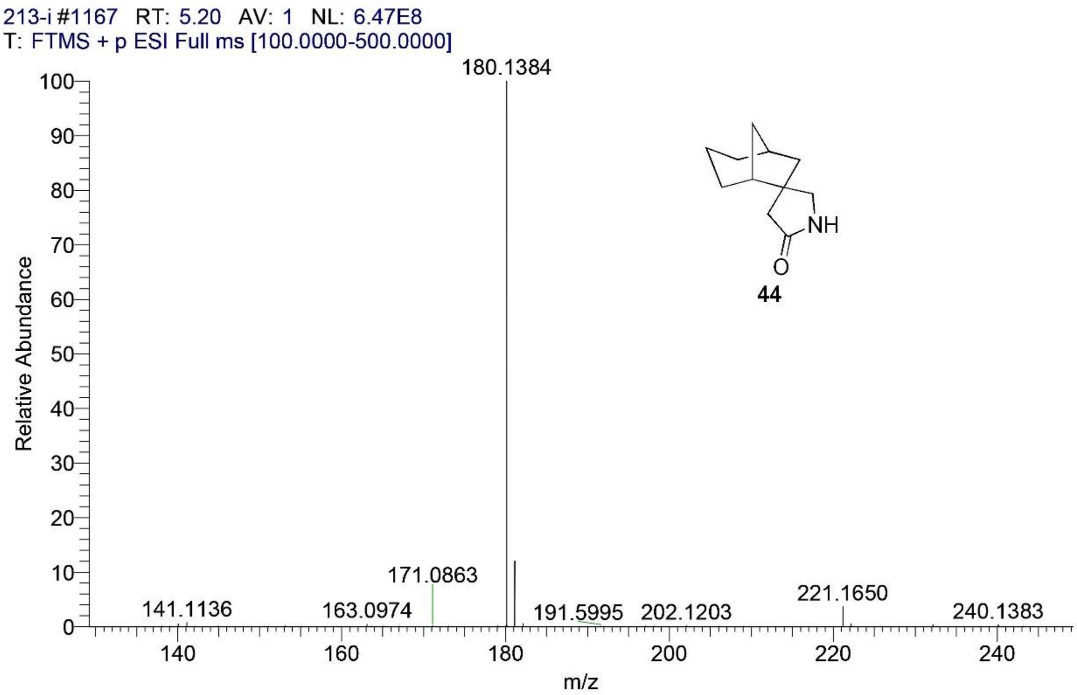

Figure S40. HR-MS spectrum of **44**

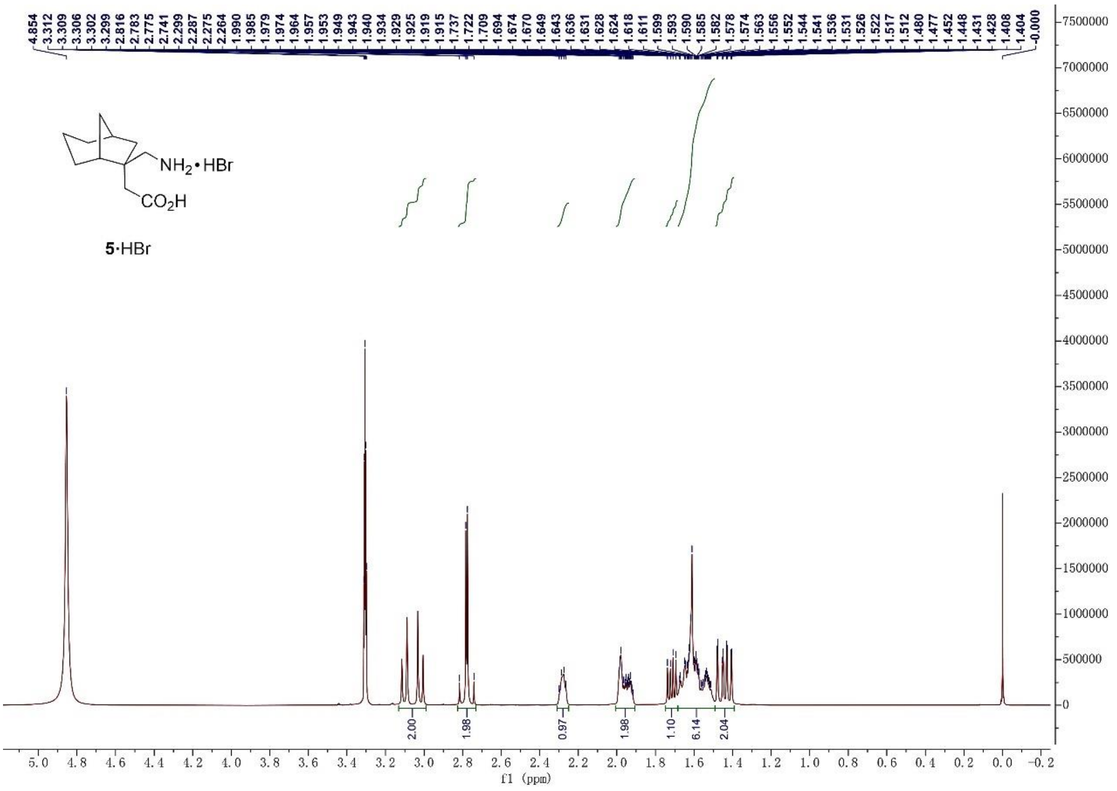

Figure S41. ^{1}H NMR spectrum of **5**•HBr (500 MHz, CD_3OD)

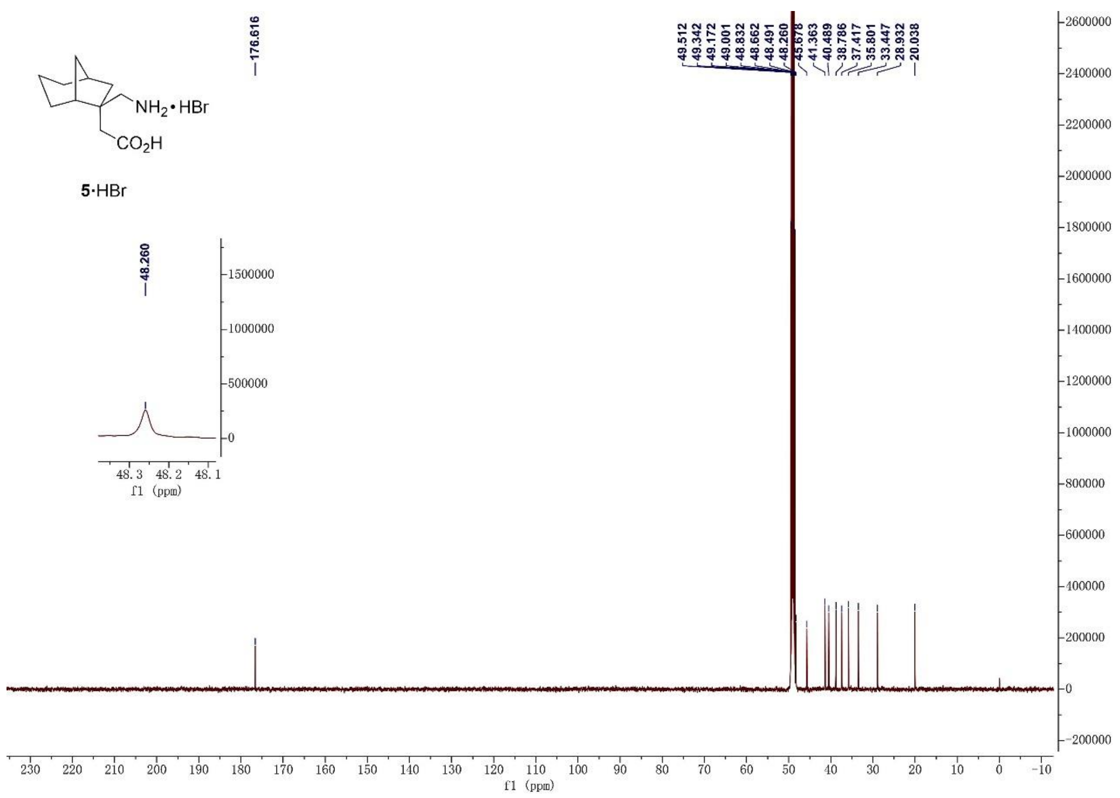

Figure S42. ^{13}C NMR spectrum of **5**•HBr (126 MHz, CD_3OD)

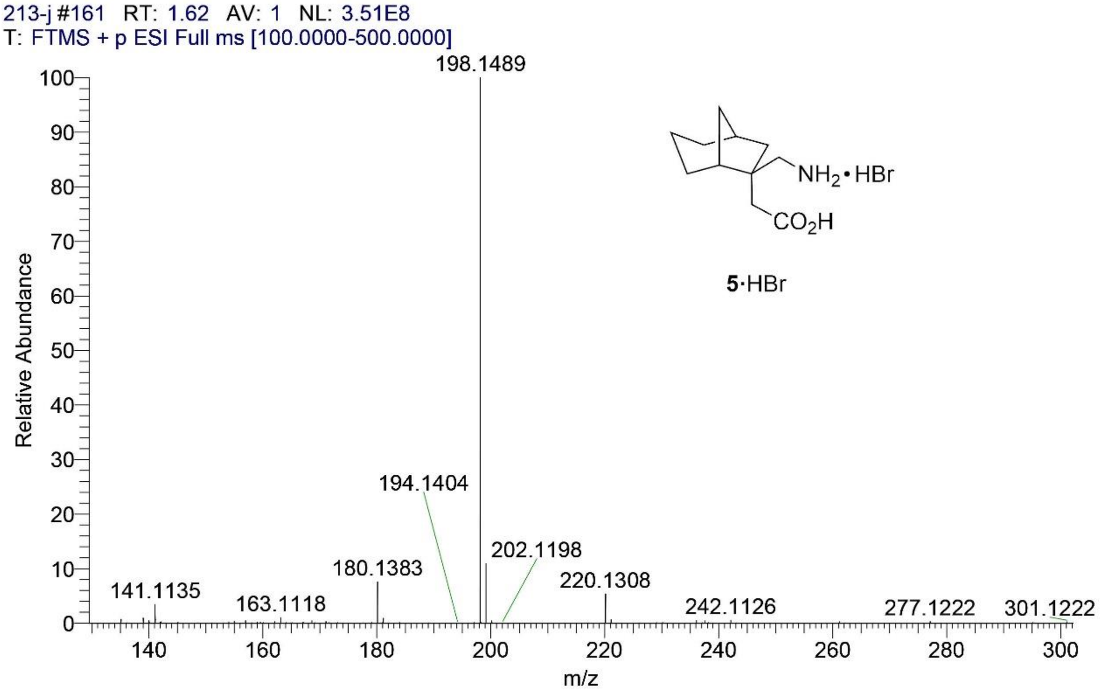

Figure S43. HR-MS spectrum of **5**•HBr

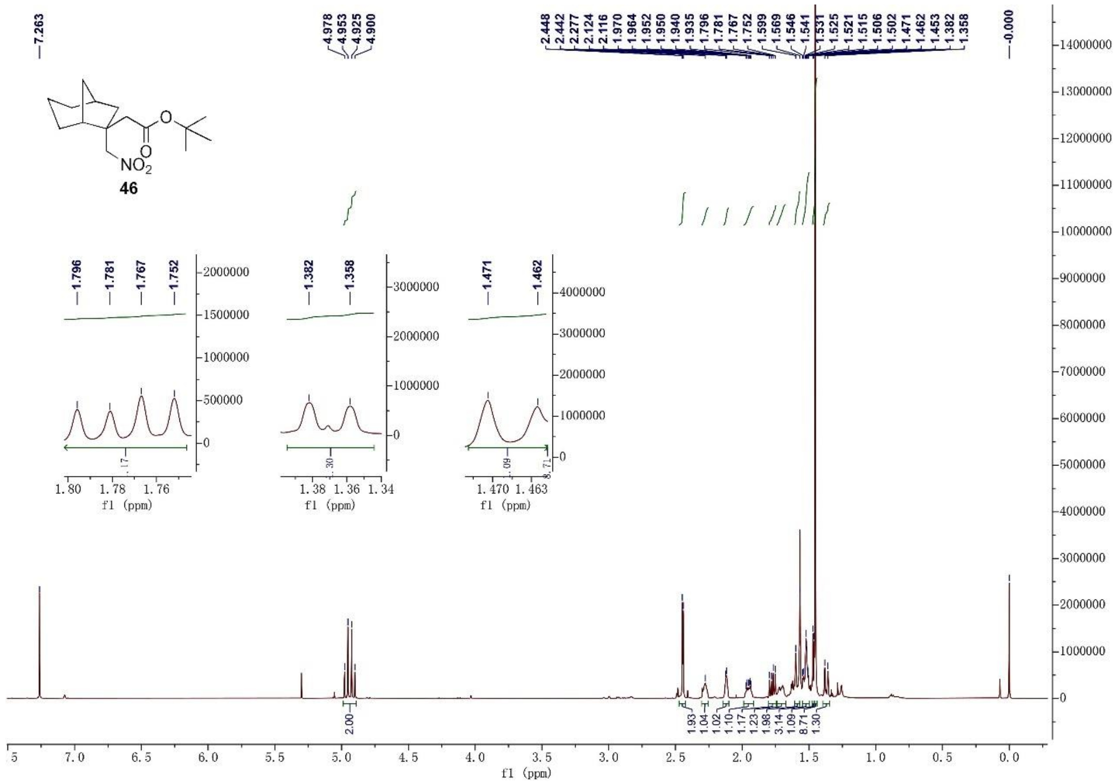

Figure S44. ^{1}H NMR spectrum of **46** (500 MHz, $CDCl_3$)

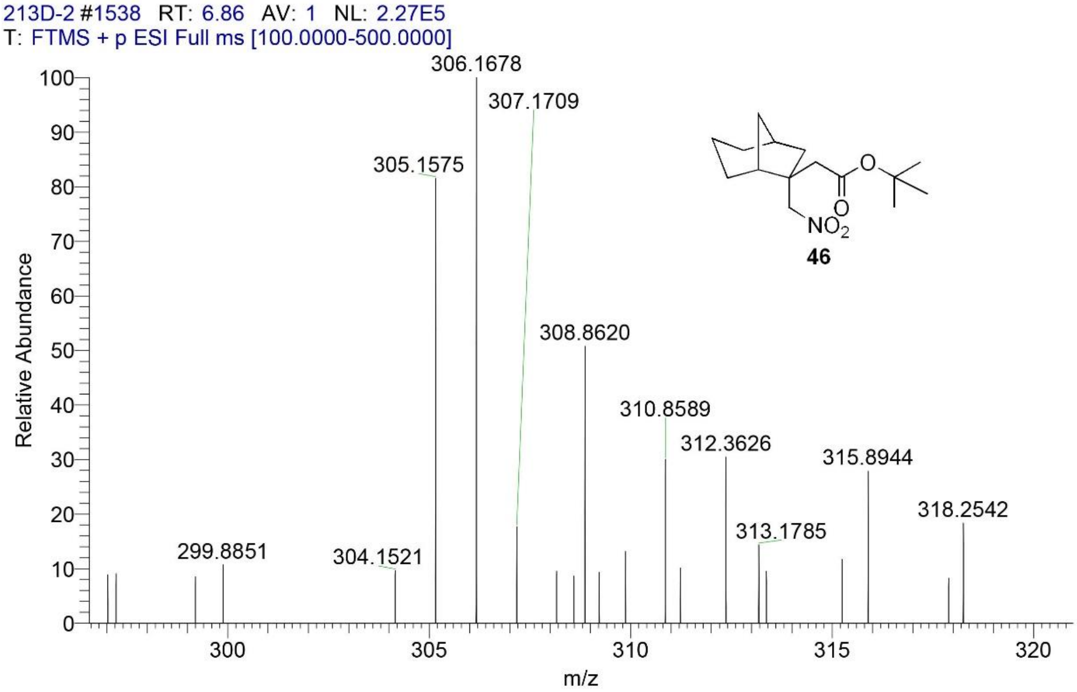

Figure S45. HR-MS spectrum of **46**

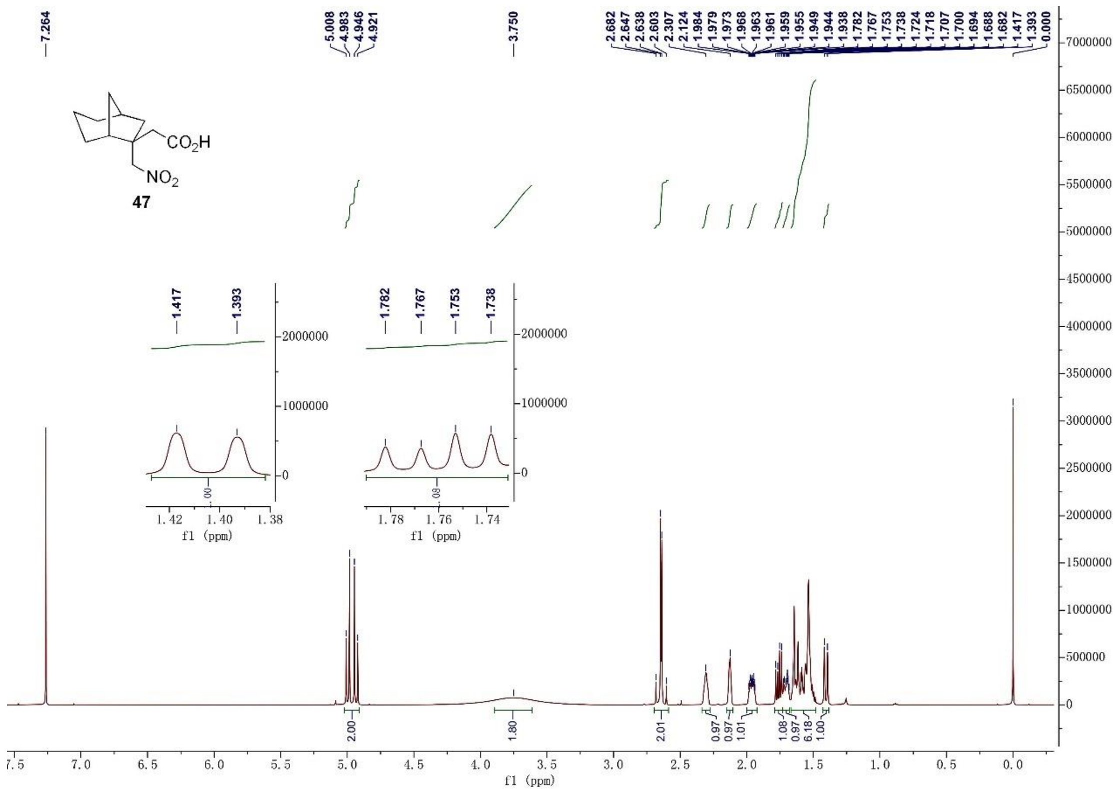

Figure S46. ^{1}H NMR spectrum of **47** (500 MHz, $CDCl_3$)

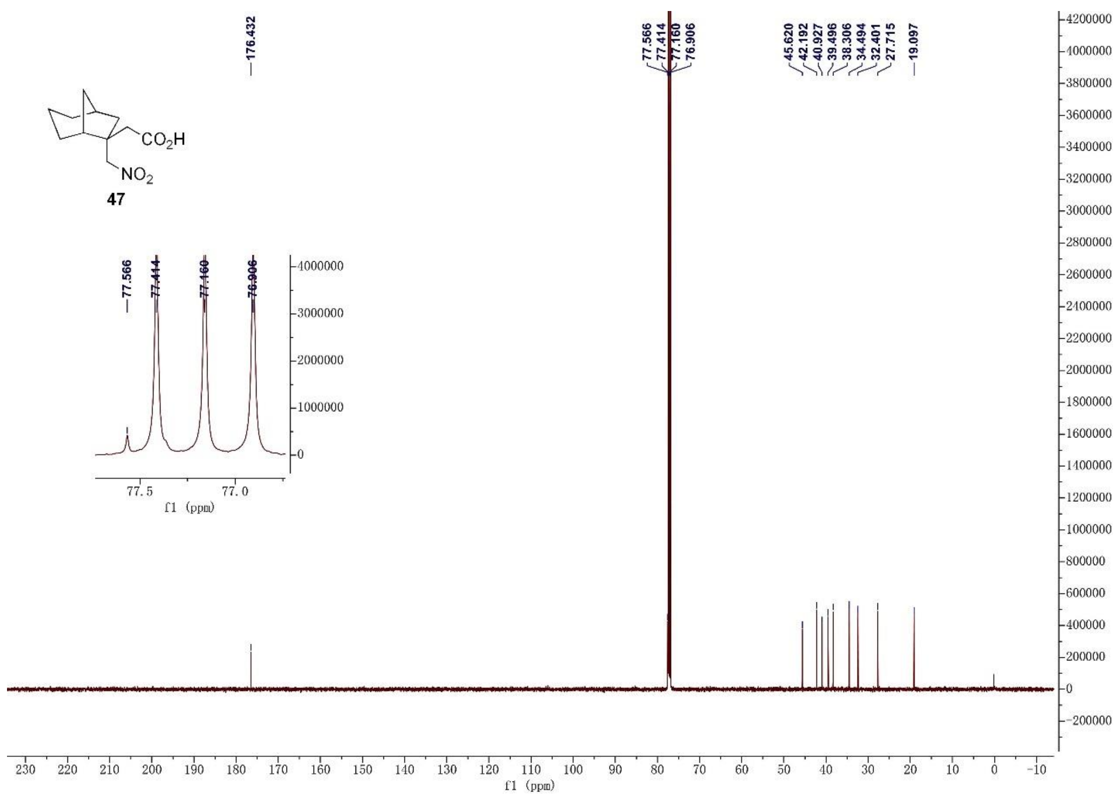

Figure S47. ^{13}C NMR spectrum of **47** (126 MHz, $CDCl_3$)

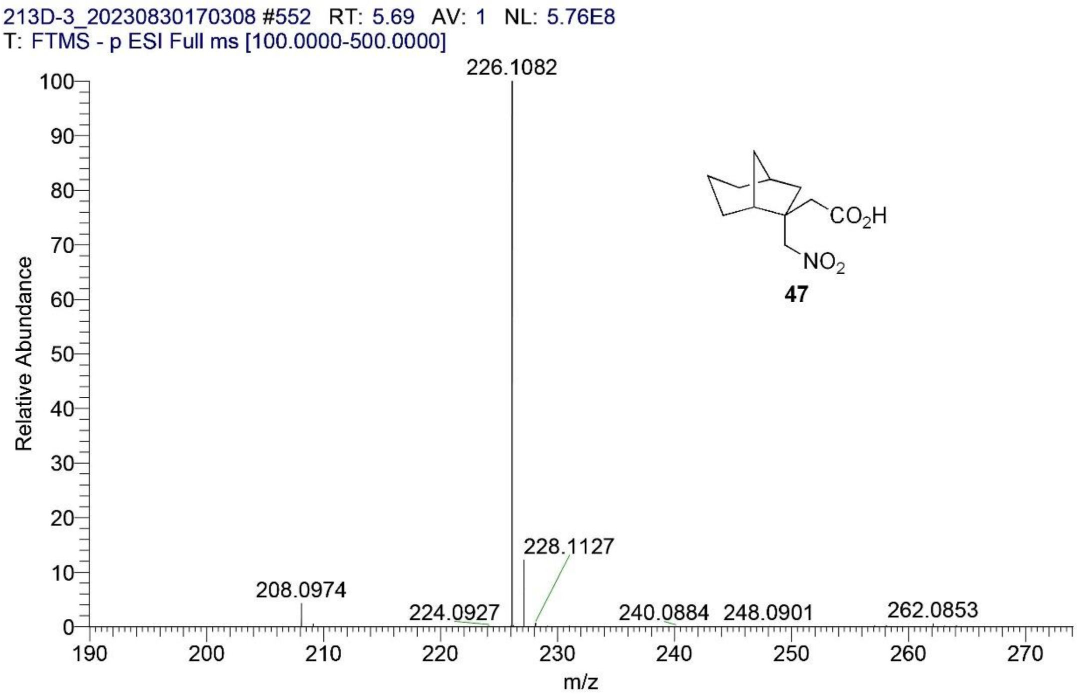

Figure S48. HR-MS spectrum of **47**

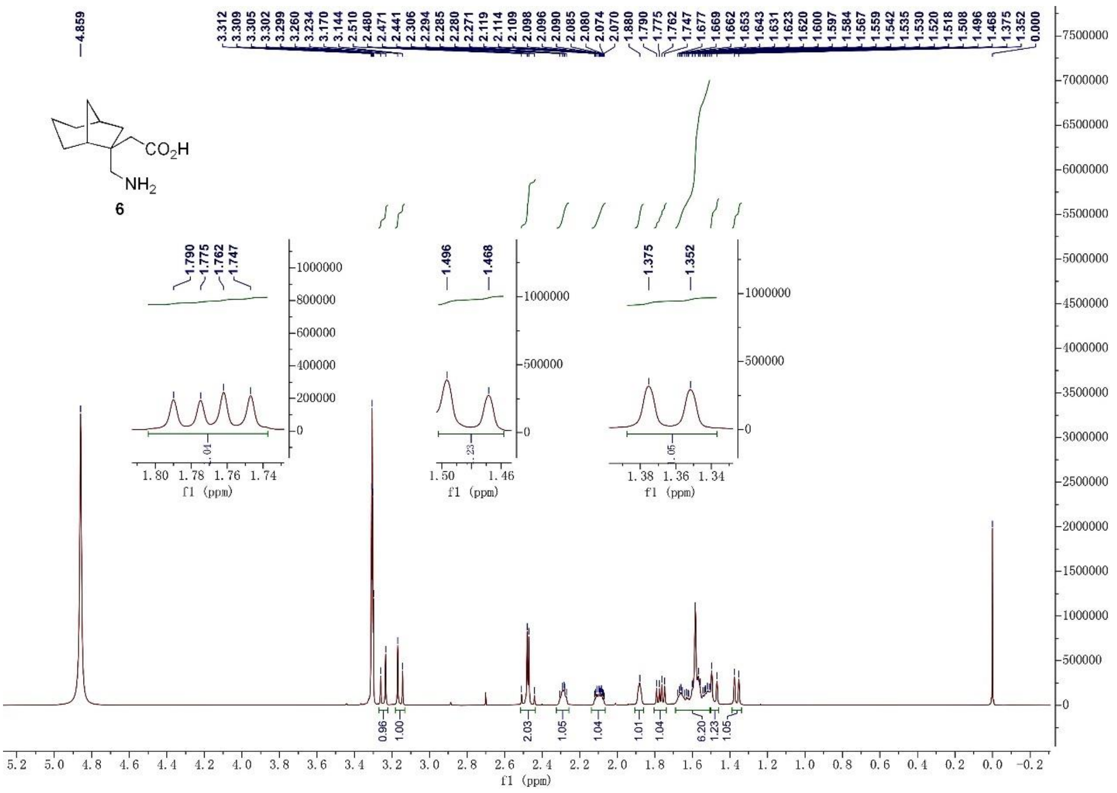

Figure S49. ^{1}H NMR spectrum of **6** (500 MHz, CD_3OD)

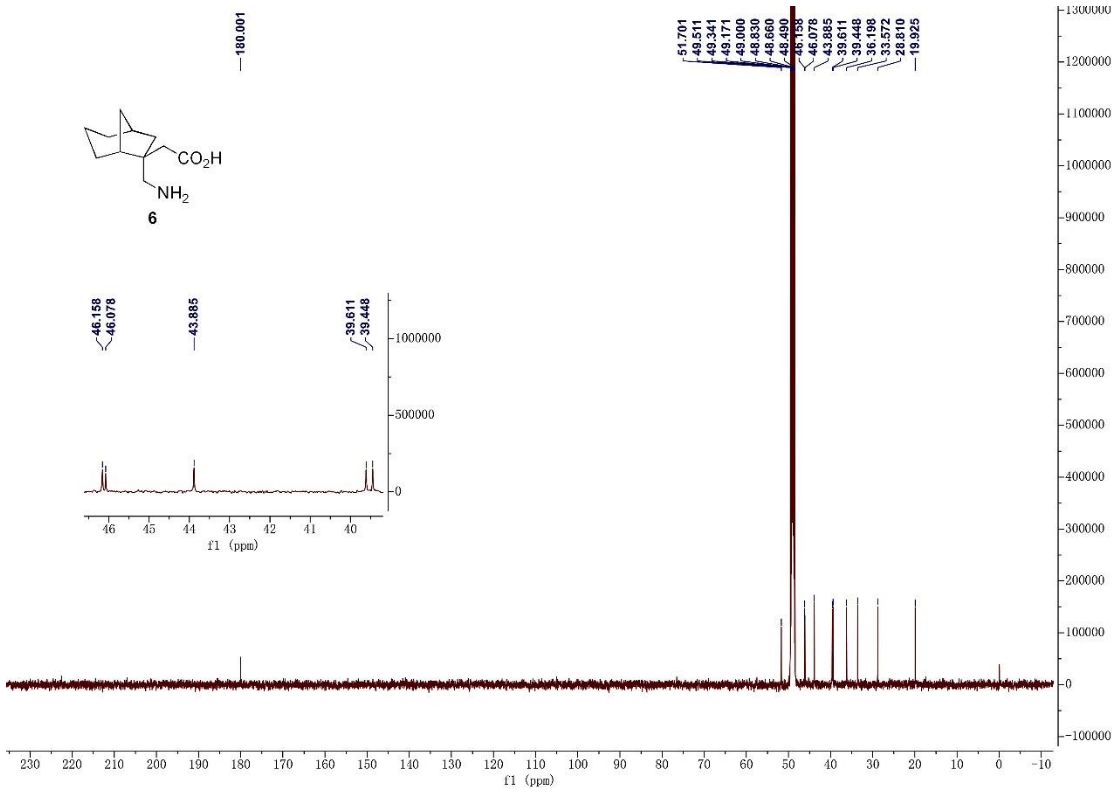

Figure S50. ^{13}C NMR spectrum of **6** (126 MHz, CD_3OD)

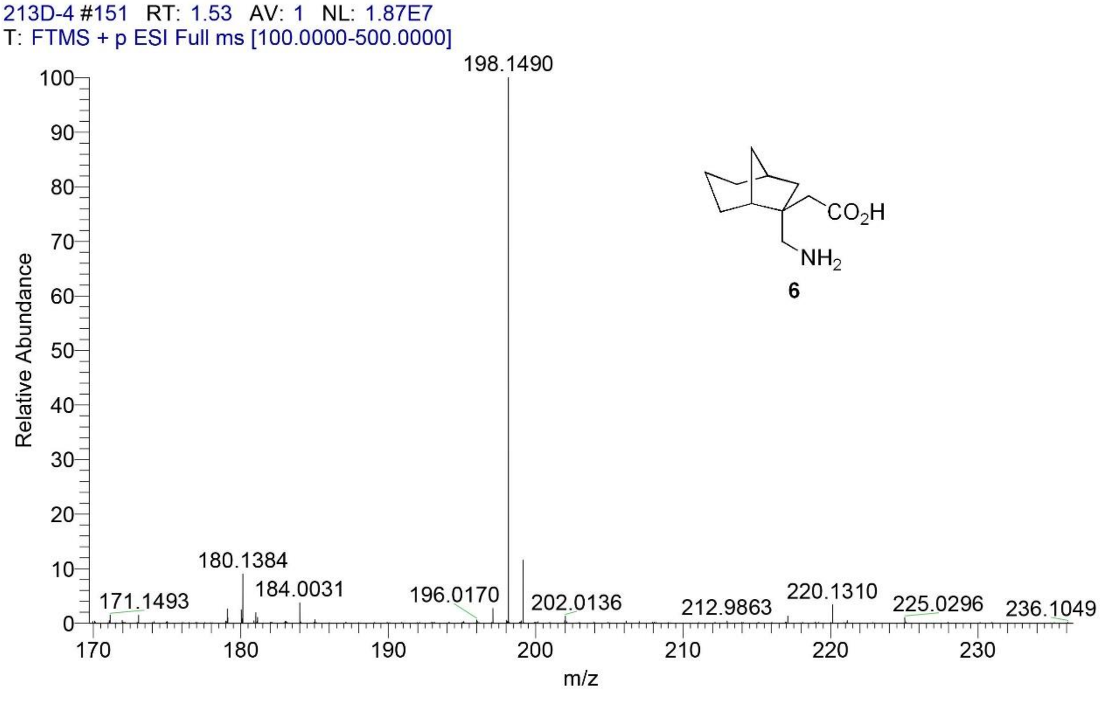

Figure S51. HR-MS spectrum of **6**

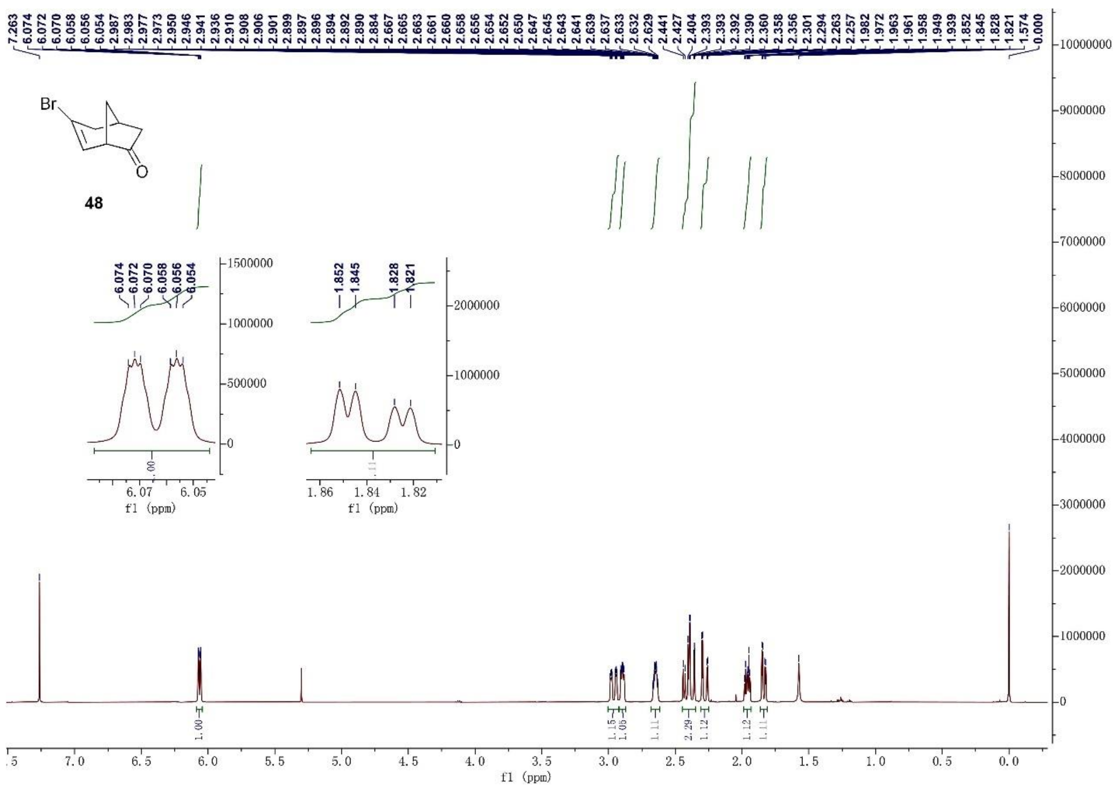

Figure S52. ^{1}H NMR spectrum of **48** (500 MHz, $CDCl_3$)

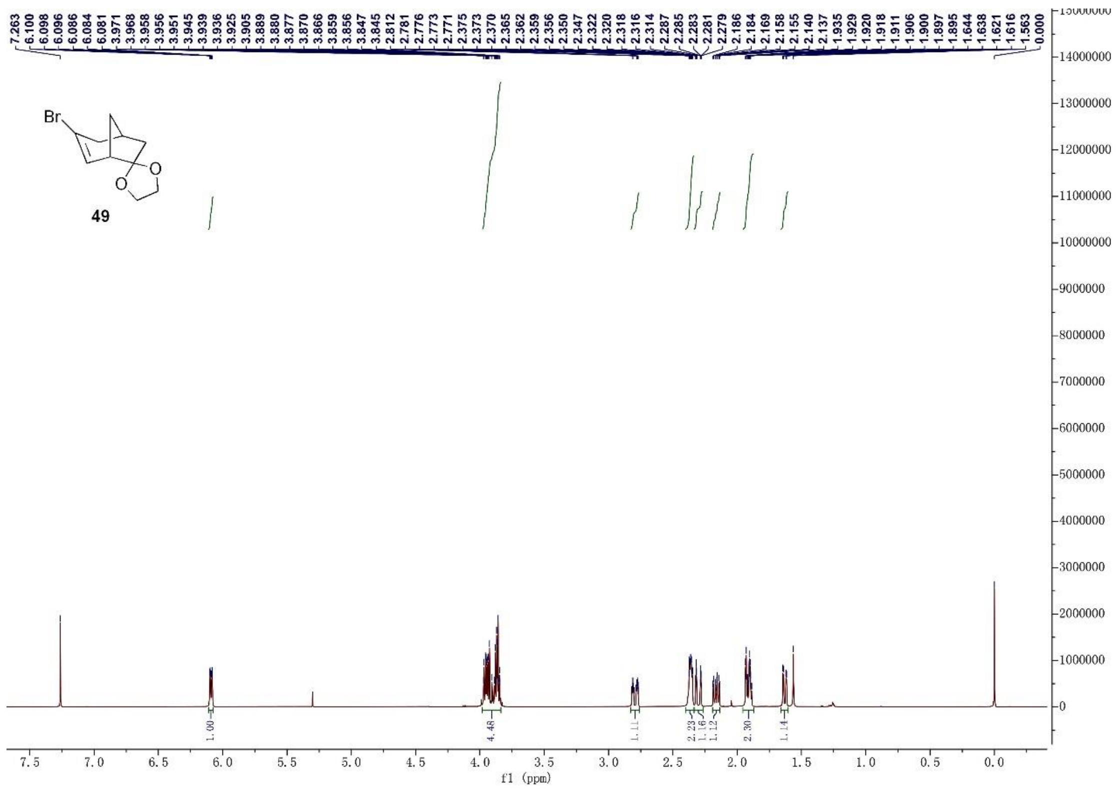

Figure S53. ^{1}H NMR spectrum of **49** (500 MHz, $CDCl_3$)

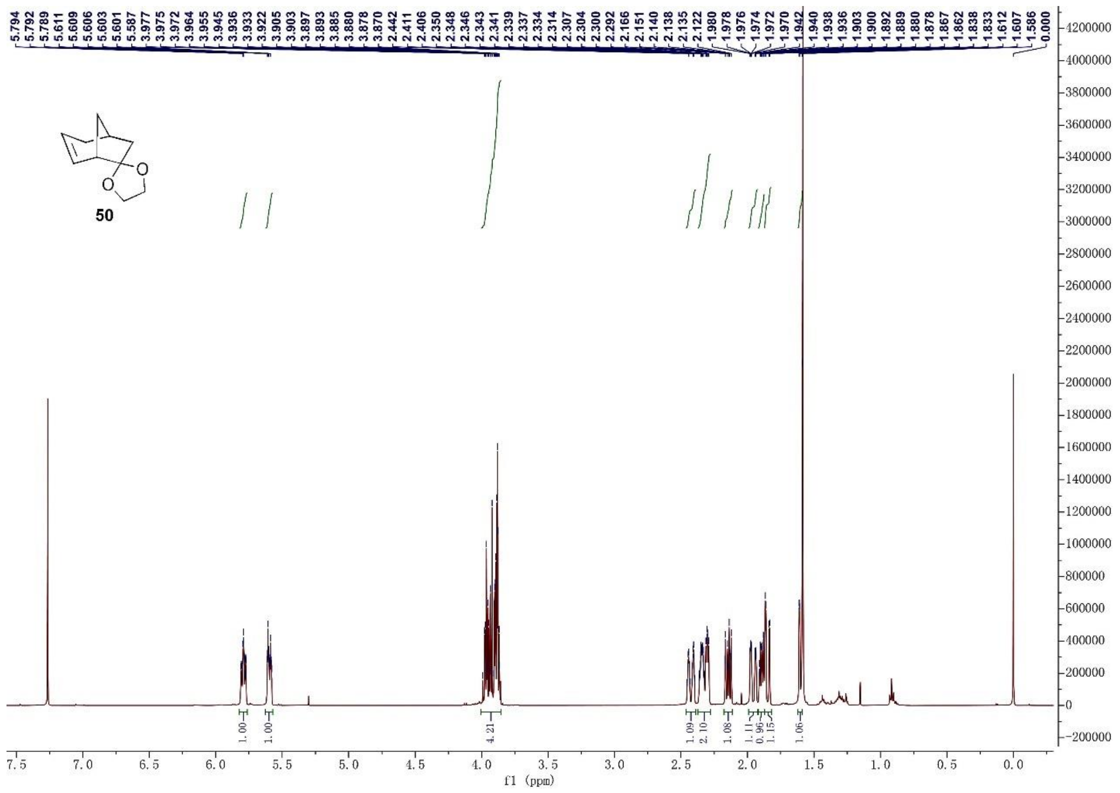

Figure S54. ^{1}H NMR spectrum of **50** (500 MHz, $CDCl_3$)

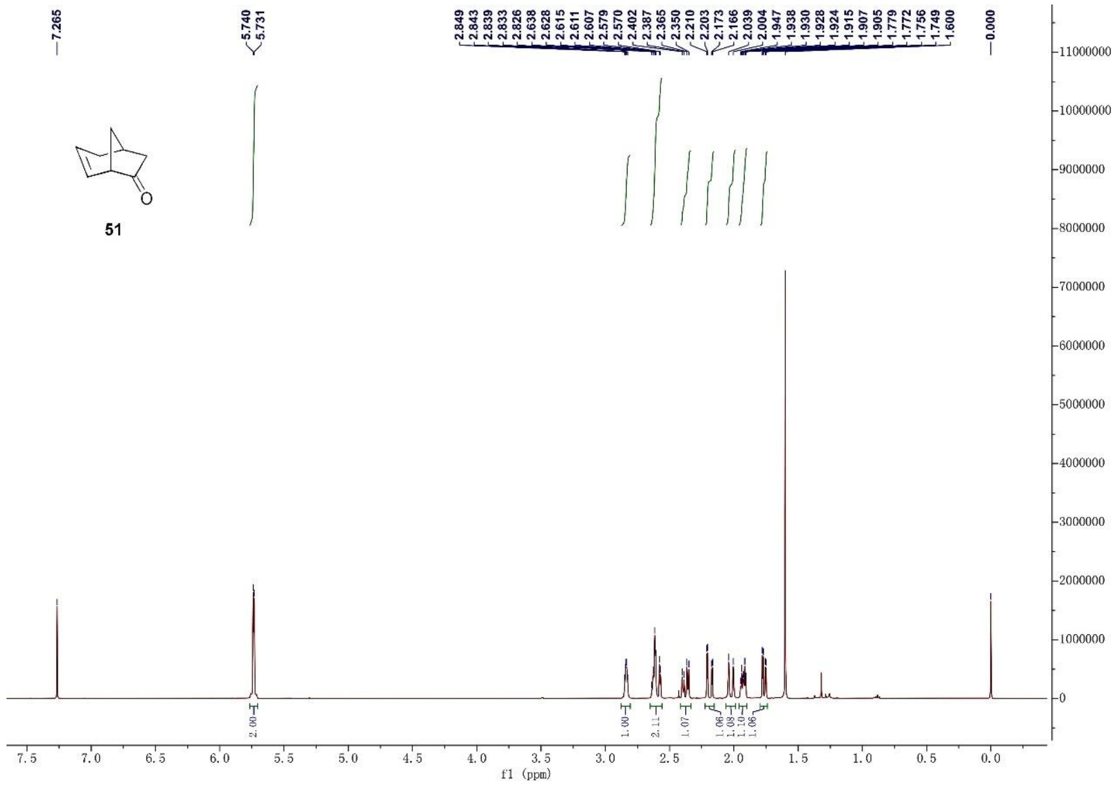

Figure S55. ^{1}H NMR spectrum of **51** (500 MHz, $CDCl_3$)

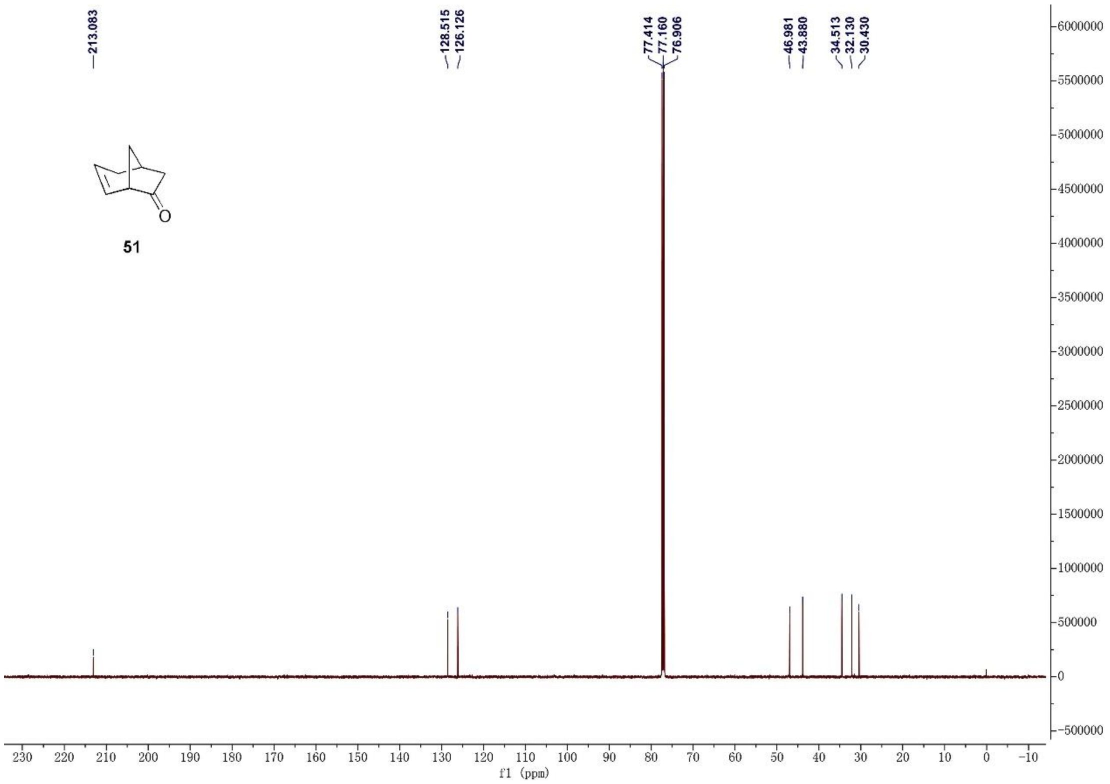

Figure S56. ^{13}C NMR spectrum of **51** (126 MHz, $CDCl_3$)

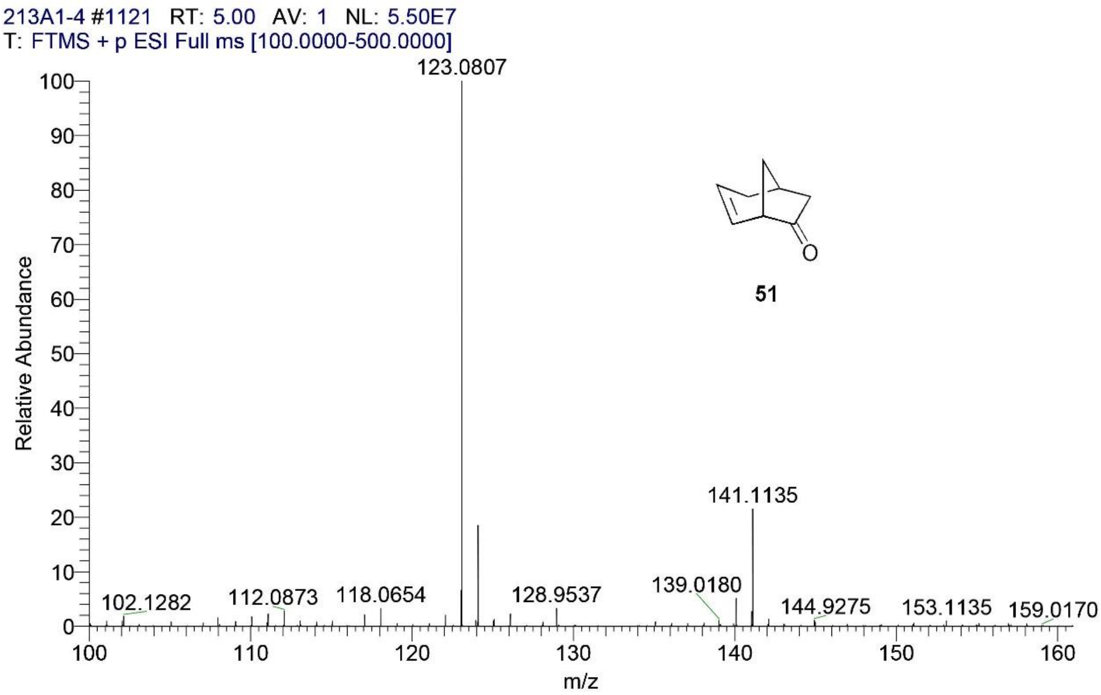

Figure S57. HR-MS spectrum of **51**

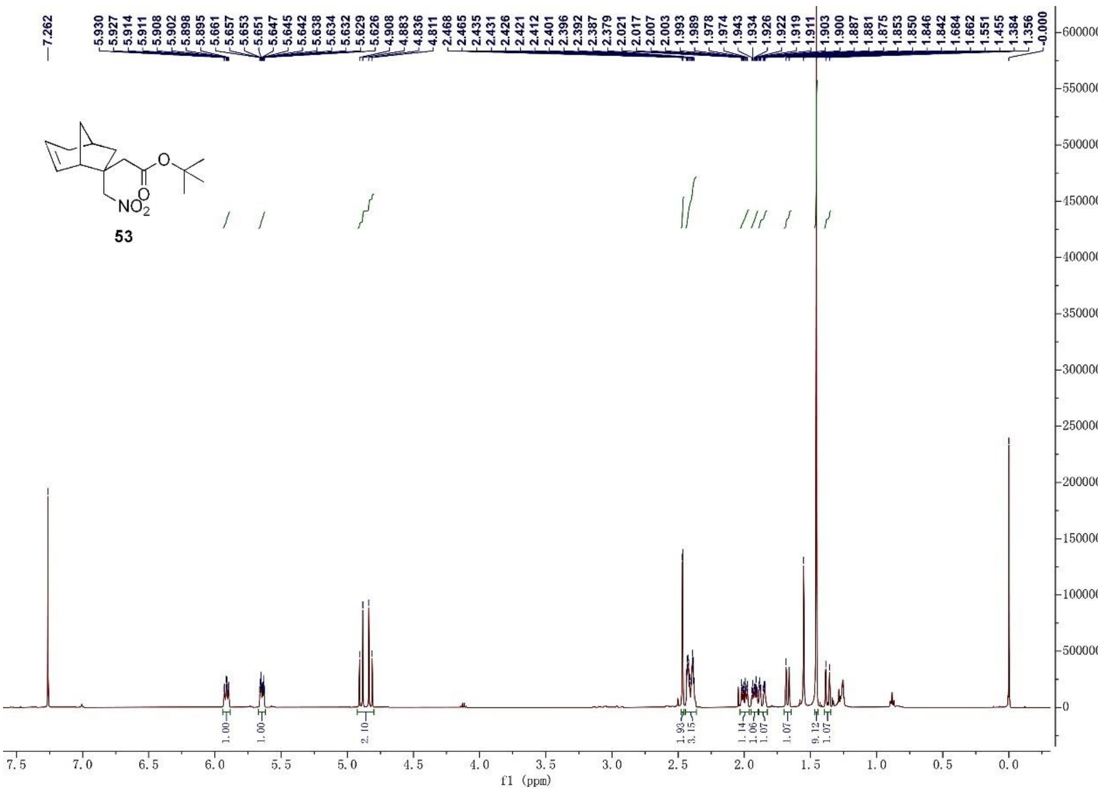

Figure S58. ^{1}H NMR spectrum of **53** (500 MHz, $CDCl_3$)

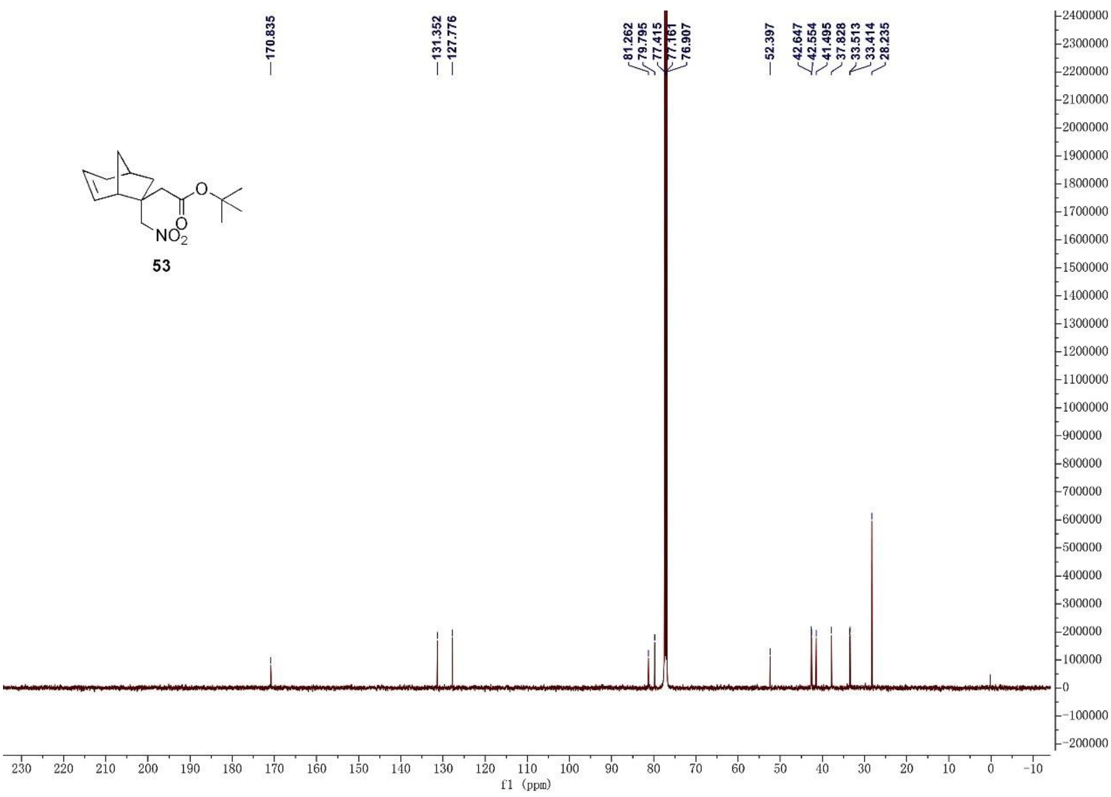

Figure S59. ^{13}C NMR spectrum of **53** (126 MHz, $CDCl_3$)

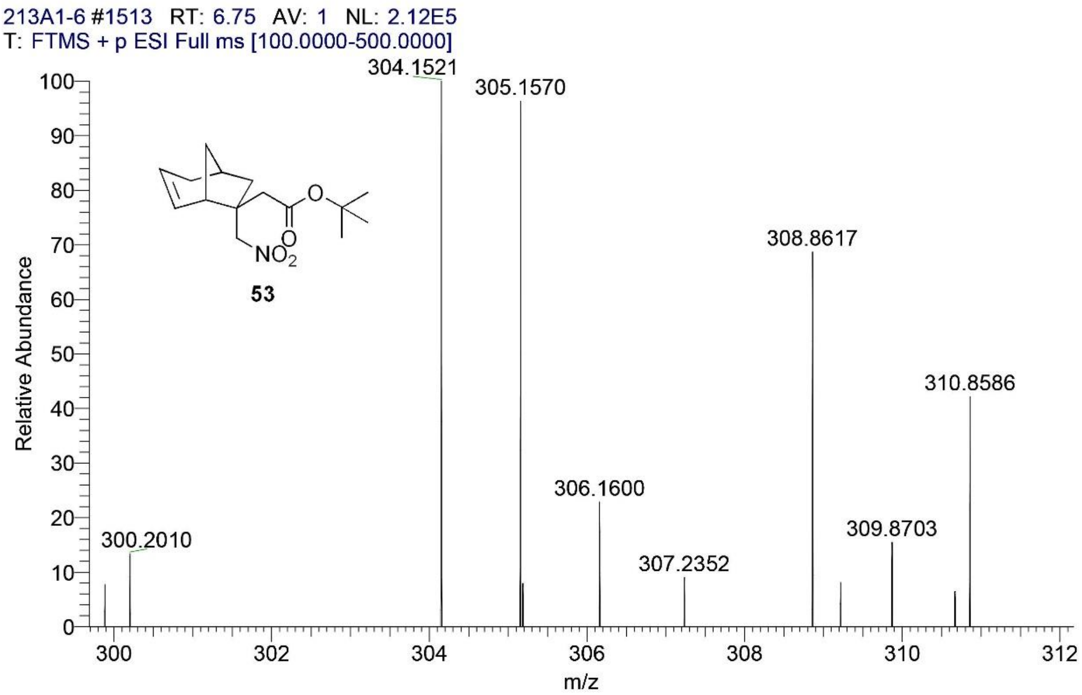

Figure S60. HR-MS spectrum of **53**

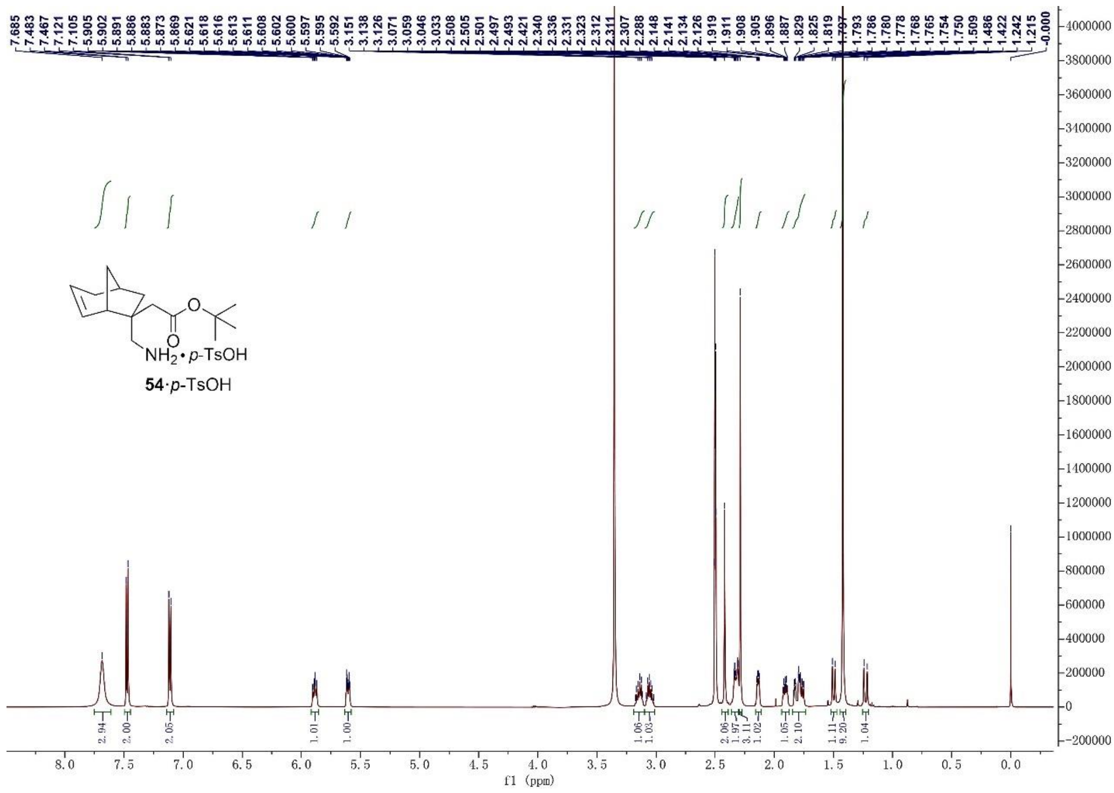

Figure S61. ^{1}H NMR spectrum of **54•*p***-TsOH (500 MHz, DMSO)

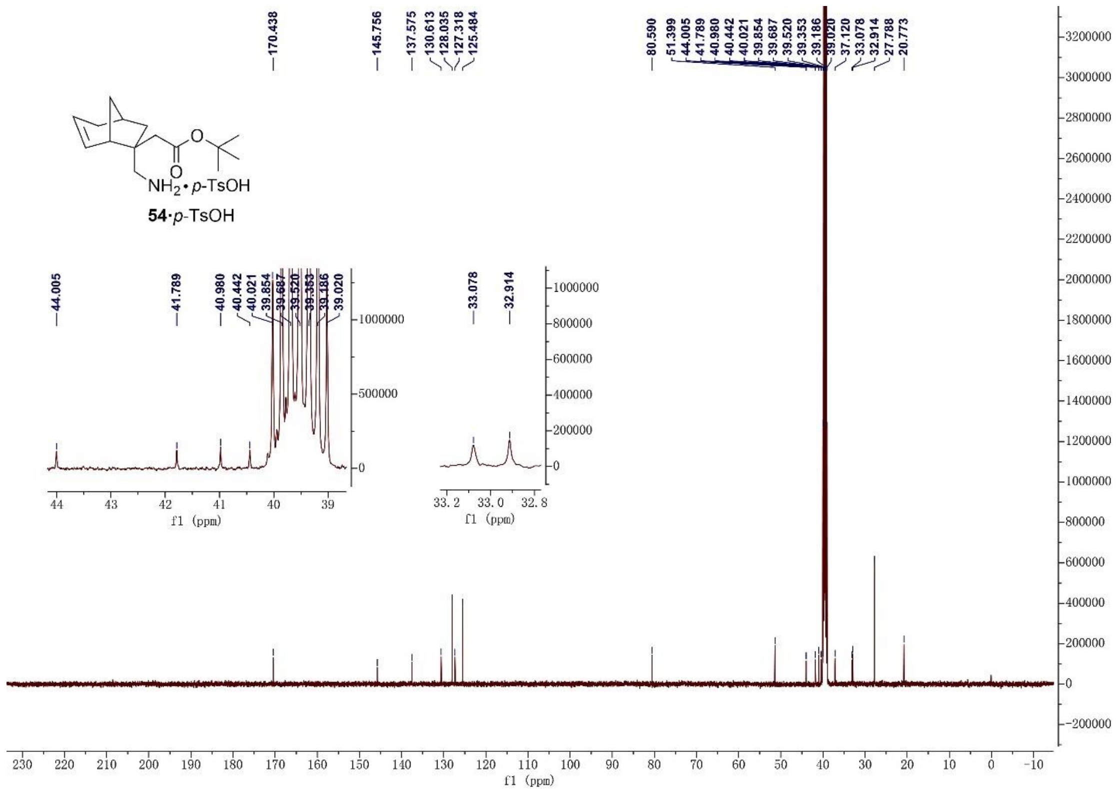

Figure S62. ^{13}C NMR spectrum of **54•***p*-TsOH (126 MHz, DMSO)

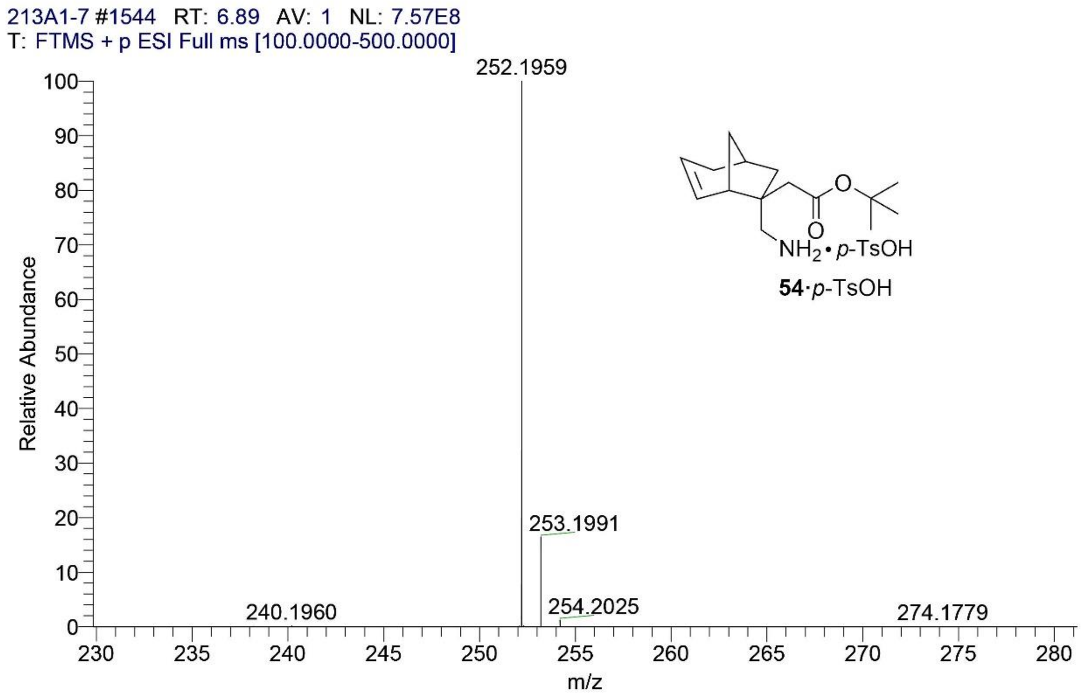

Figure S63. HR-MS spectrum of **54•***p*-TsOH

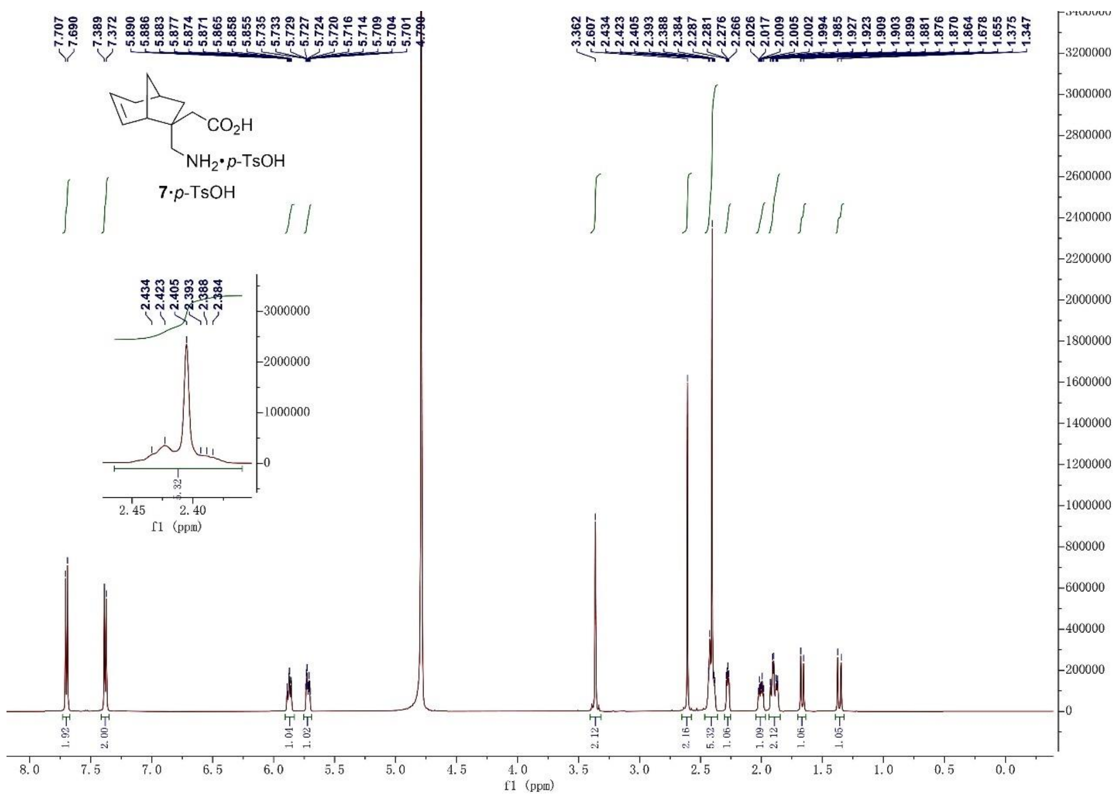

Figure S64. ^{1}H NMR spectrum of **7•***p*-TsOH (500 MHz, D_2O)

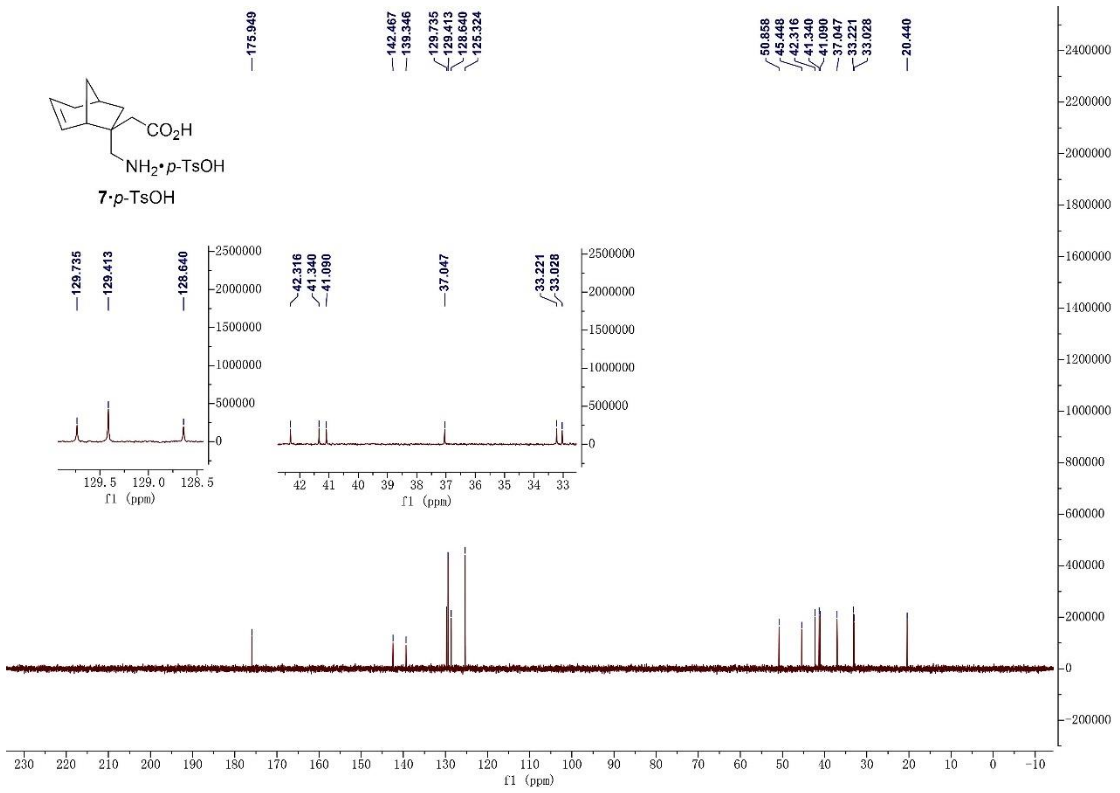

Figure S65. ^{13}C NMR spectrum of **7•***p*-TsOH (126 MHz, D_2O)

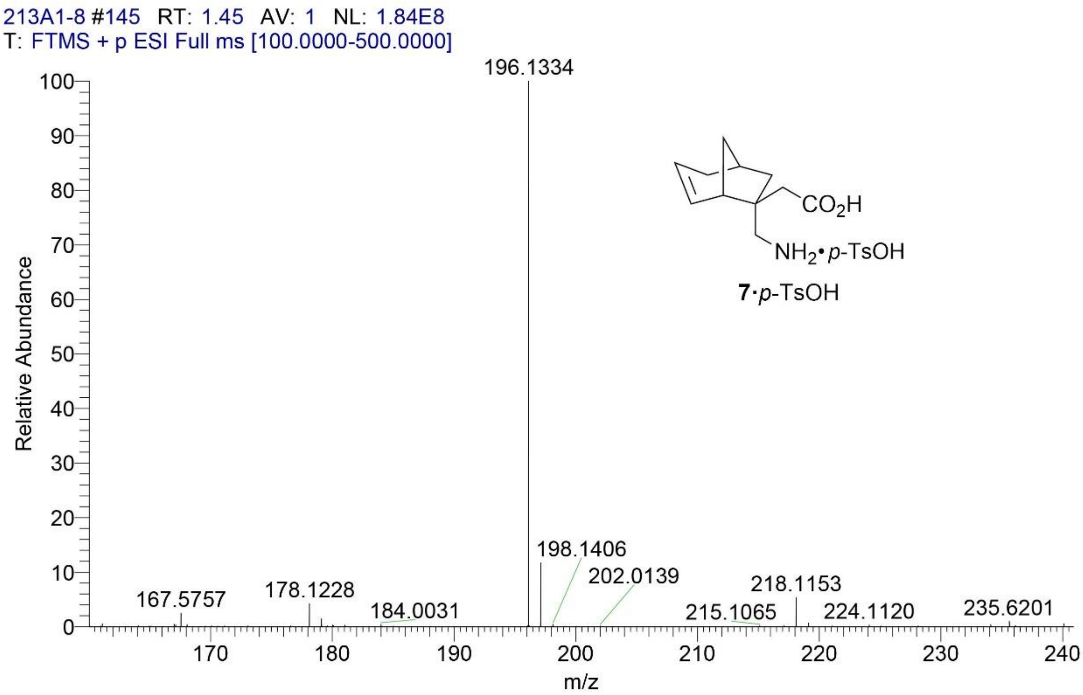

Figure S66. HR-MS spectrum of **7•**p-TsOH

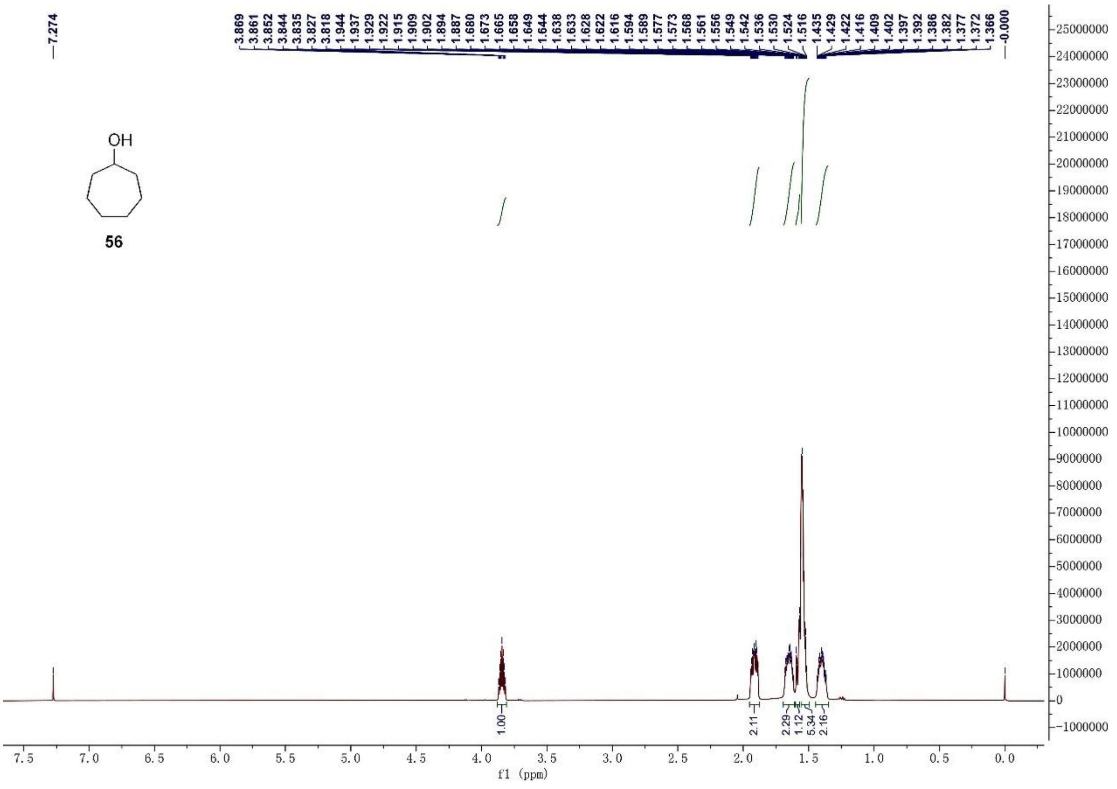

Figure S67. ^{1}H NMR spectrum of **56** (500 MHz, $CDCl_3$)

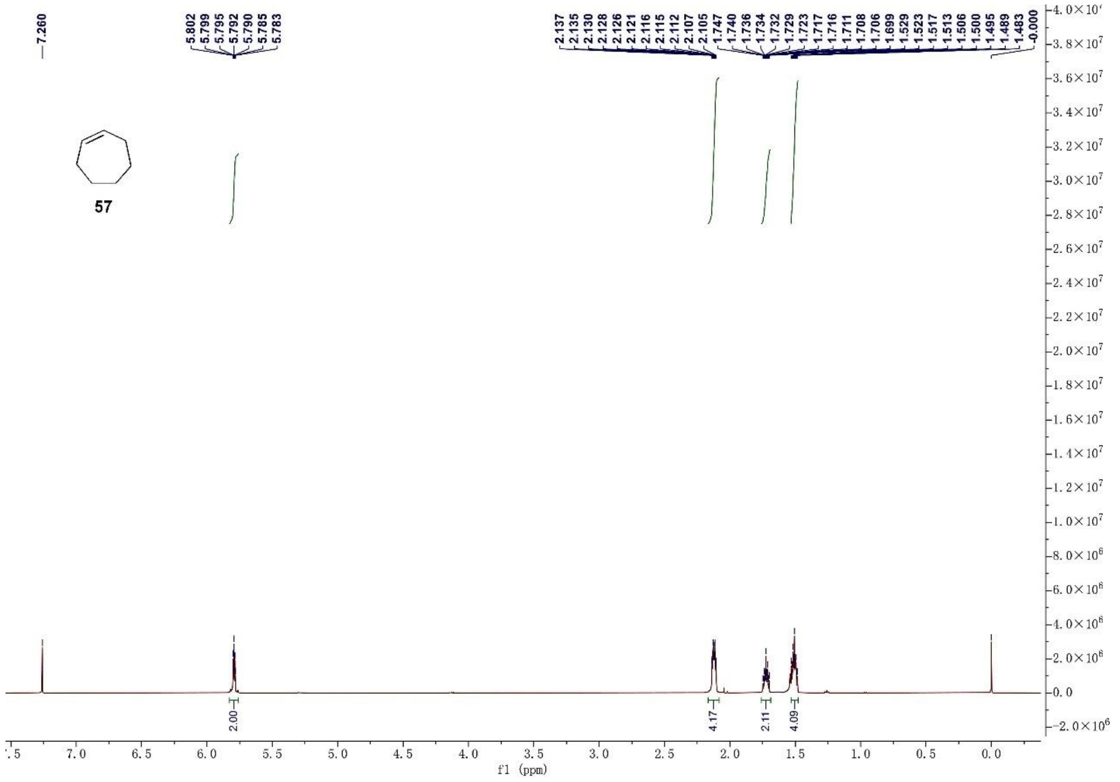

Figure S68. ^{1}H NMR spectrum of **57** (500 MHz, $CDCl_3$)

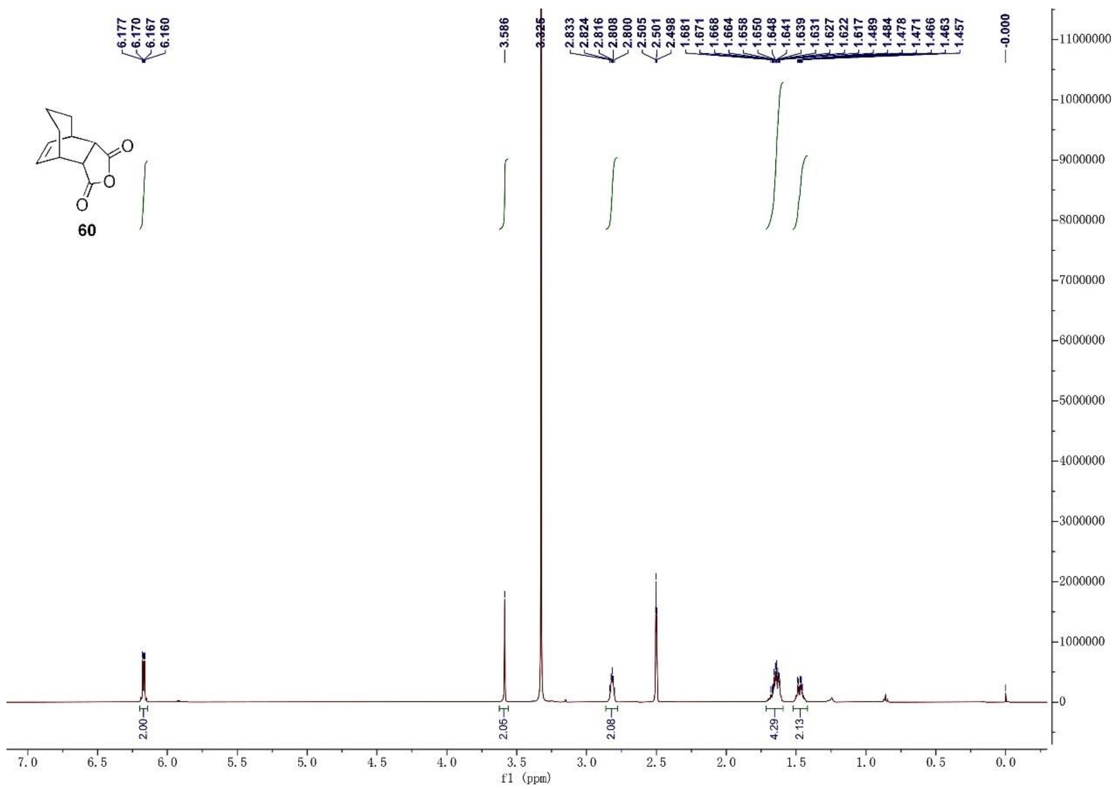

Figure S69. ^{1}H NMR spectrum of **60** (500 MHz, $CDCl_3$)

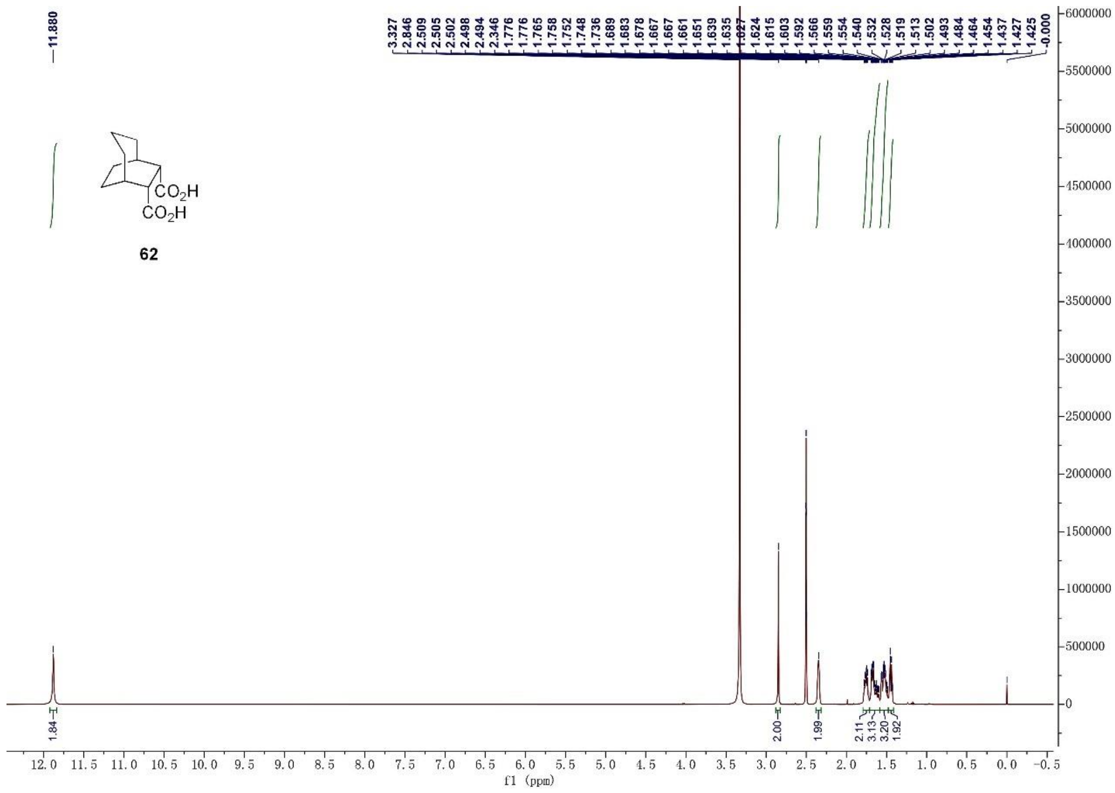

Figure S70. ^{1}H NMR spectrum of **62** (500 MHz, DMSO)

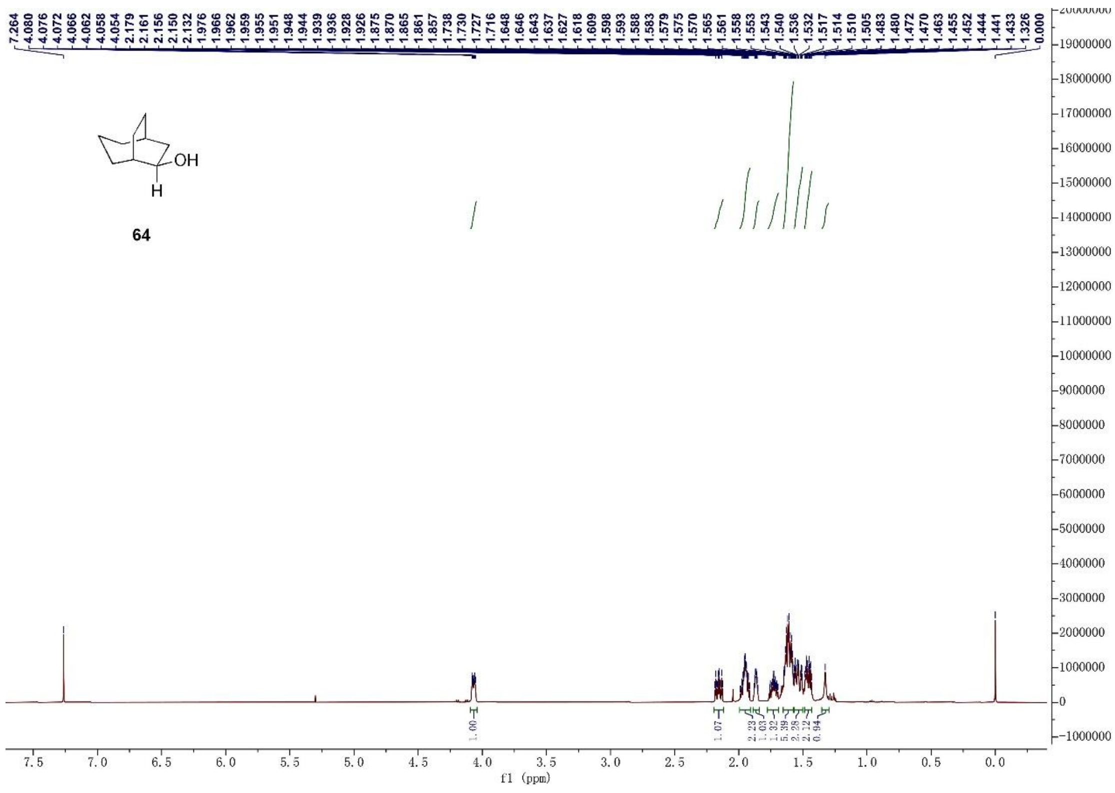

Figure S71. ^{1}H NMR spectrum of **64** (500 MHz, $CDCl_3$)

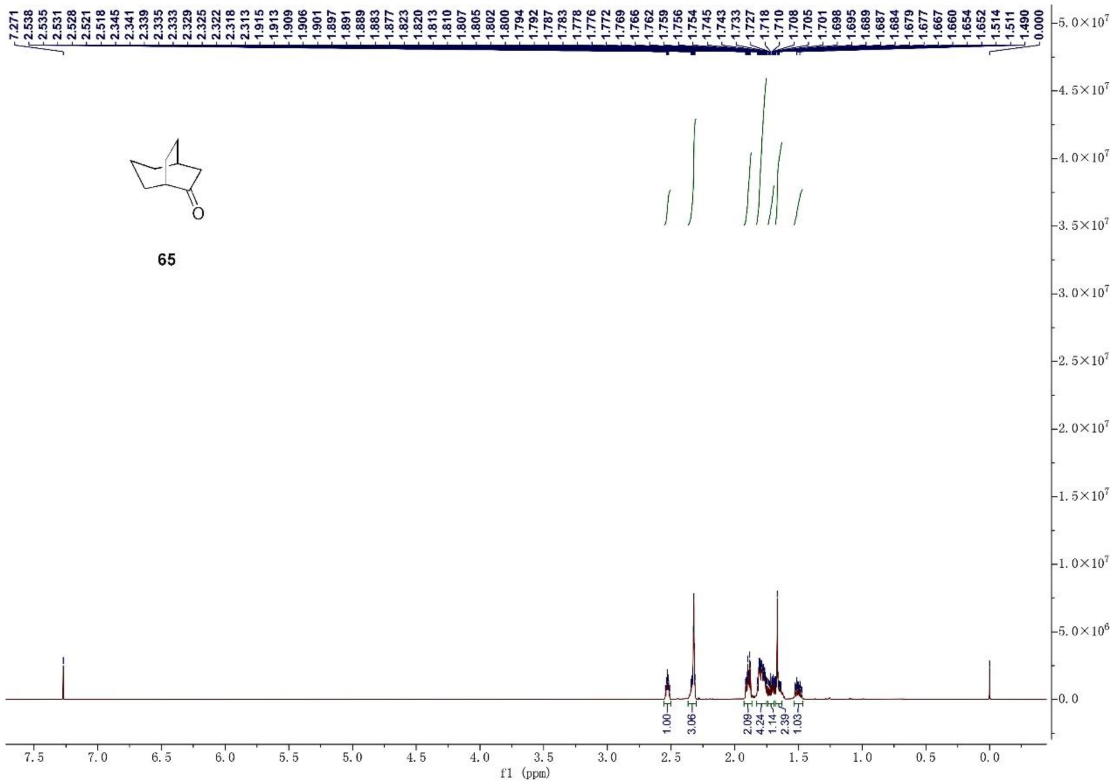

Figure S72. ^{1}H NMR spectrum of **65** (500 MHz, $CDCl_3$)

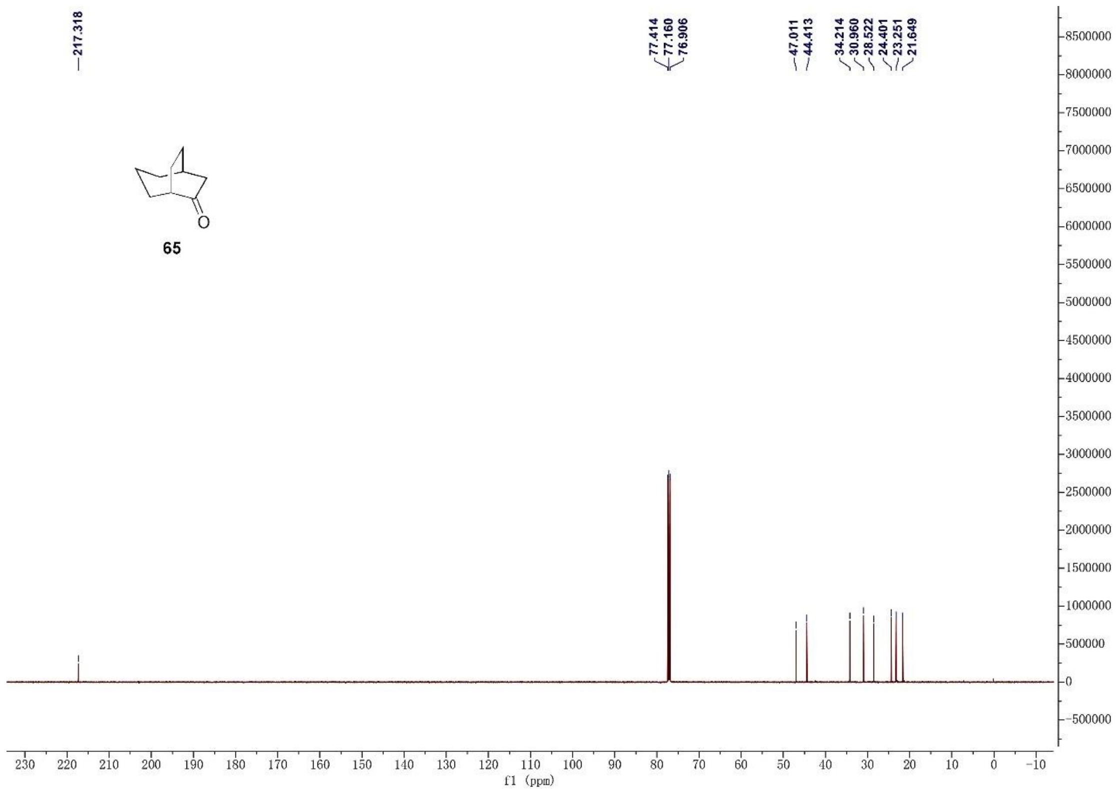

Figure S73. ^{13}C NMR spectrum of **65** (126 MHz, $CDCl_3$)

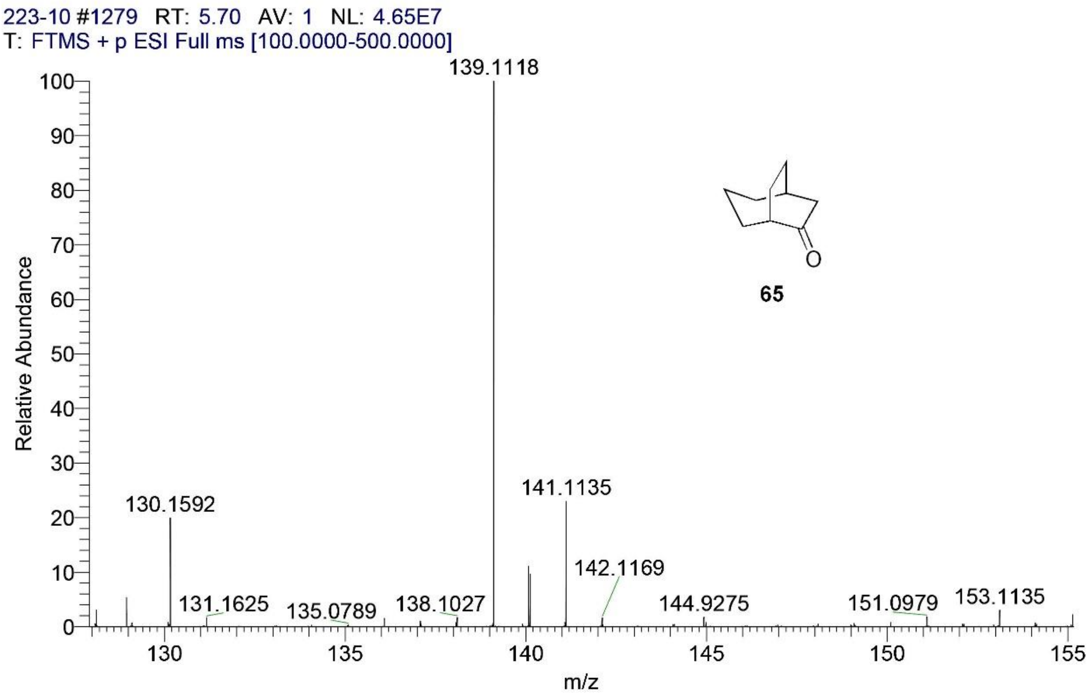

Figure S74. HR-MS spectrum of **65**

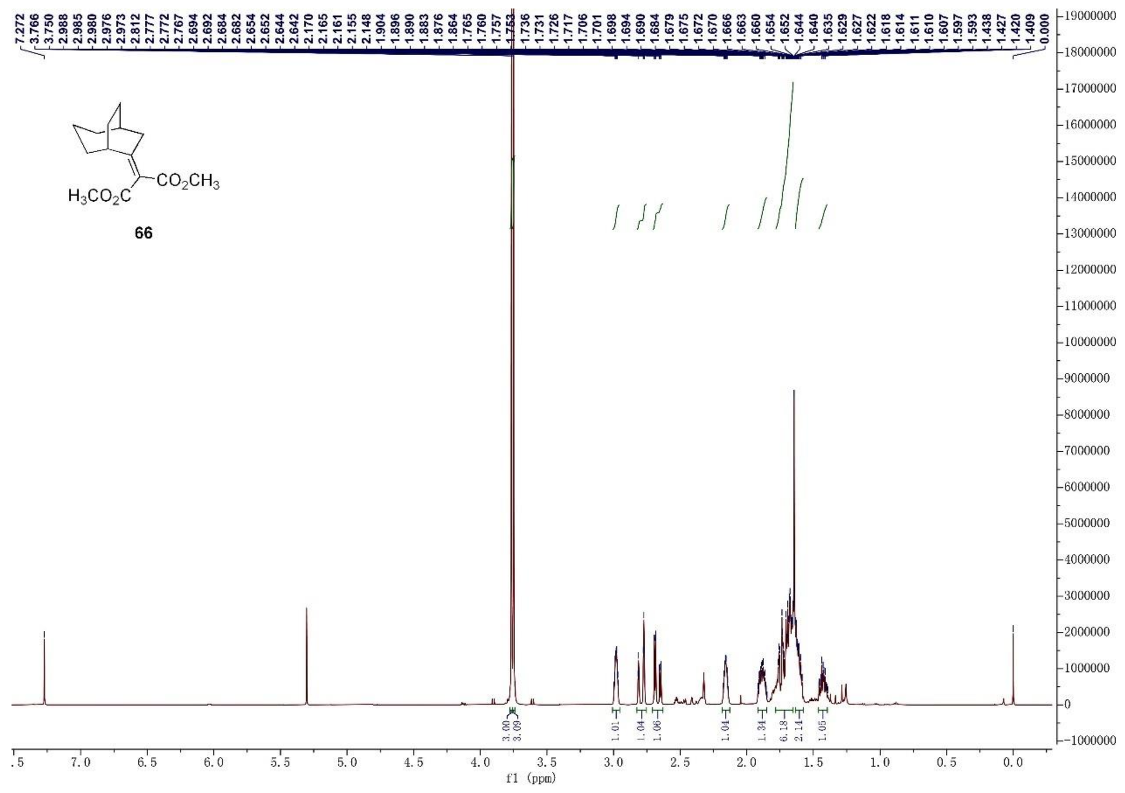

Figure S75. ^{1}H NMR spectrum of **66** (500 MHz, $CDCl_3$)

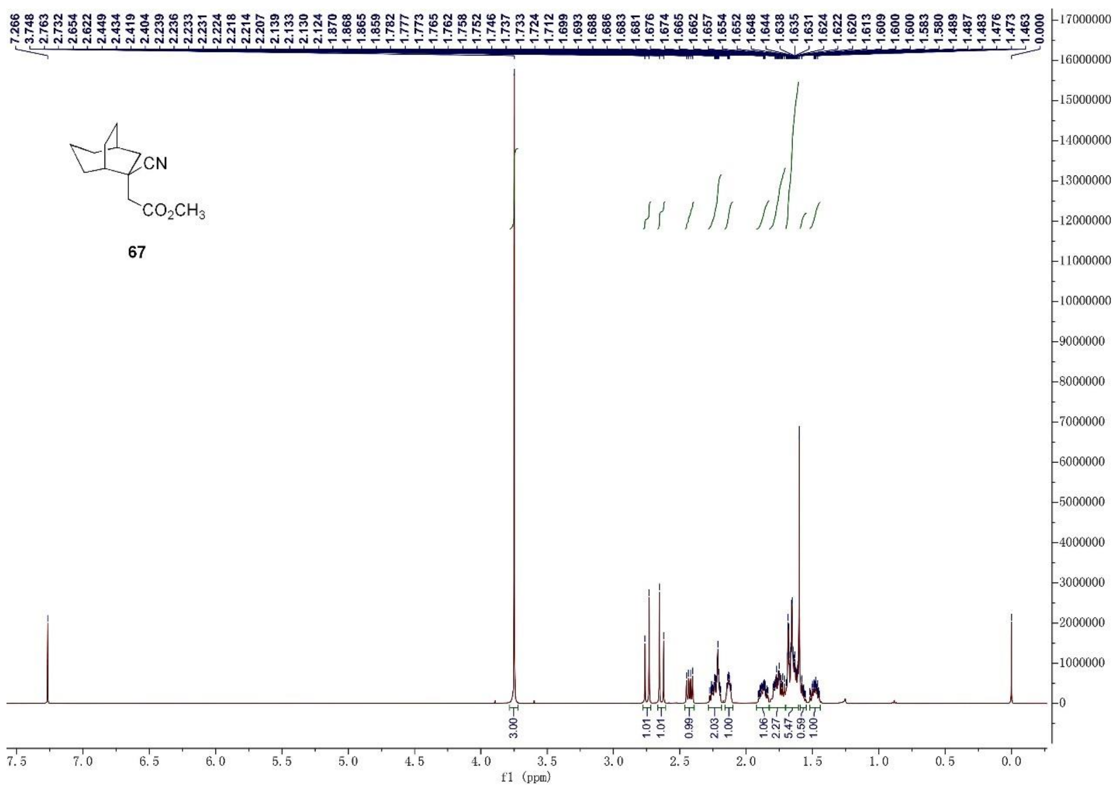

Figure S76. ^{1}H NMR spectrum of **67** (500 MHz, $CDCl_3$)

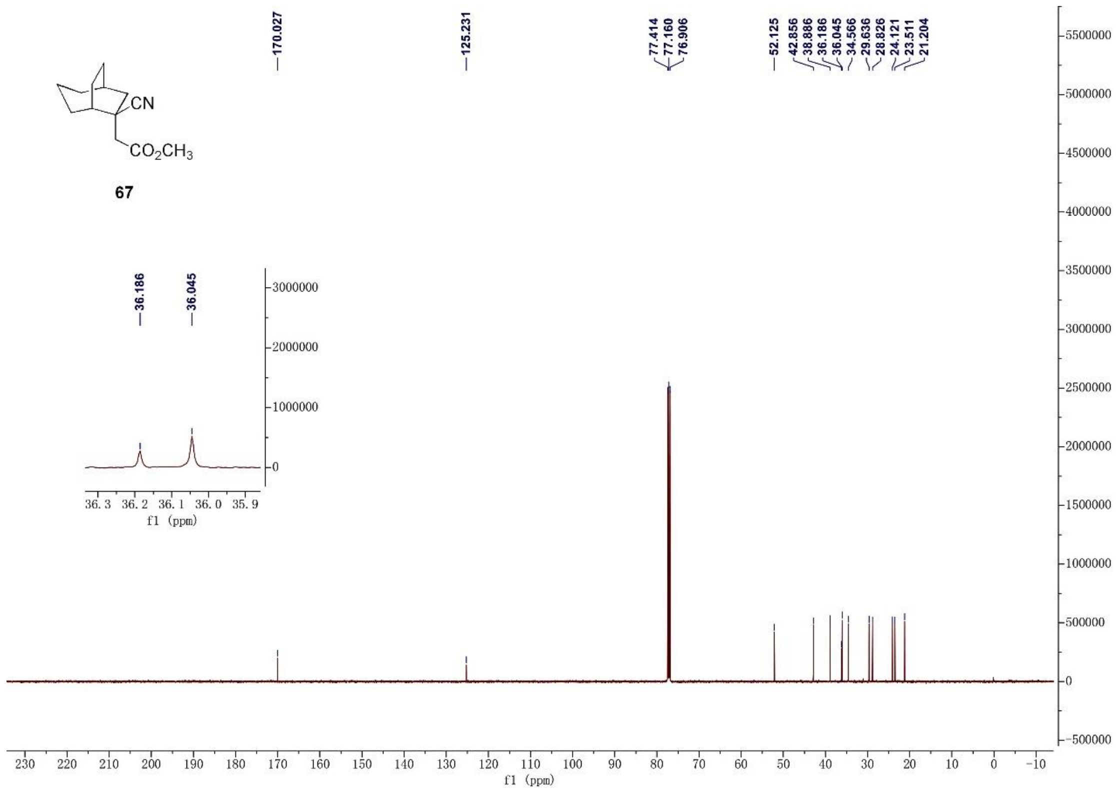

Figure S77. ^{13}C NMR spectrum of **67** (126 MHz, $CDCl_3$)

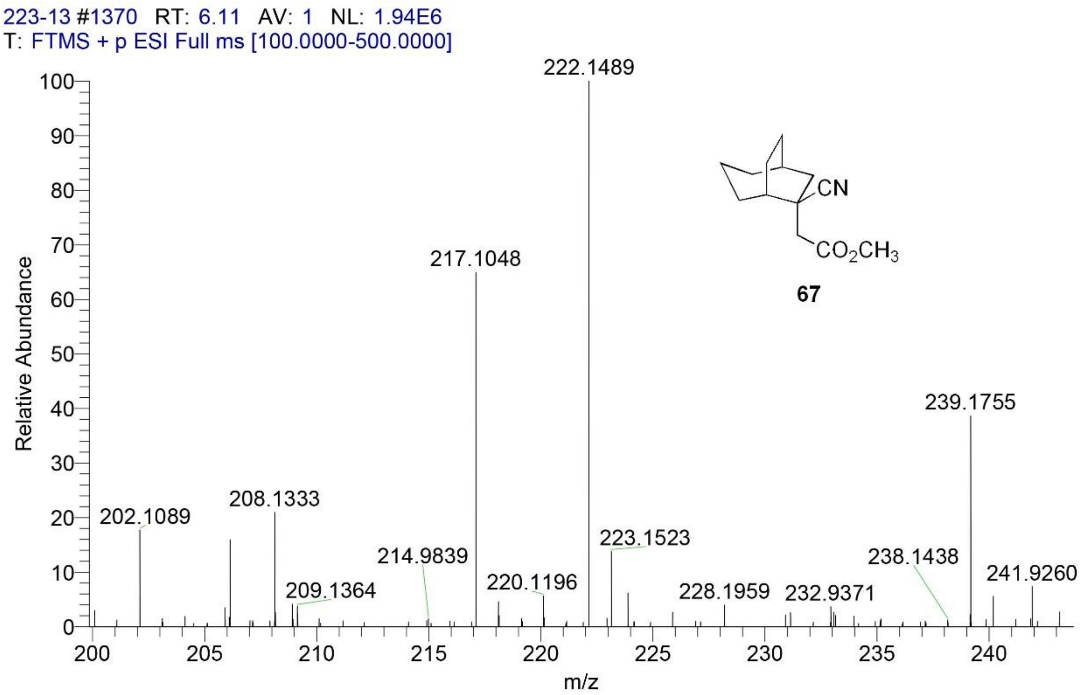

Figure S78. HR-MS spectrum of **67**

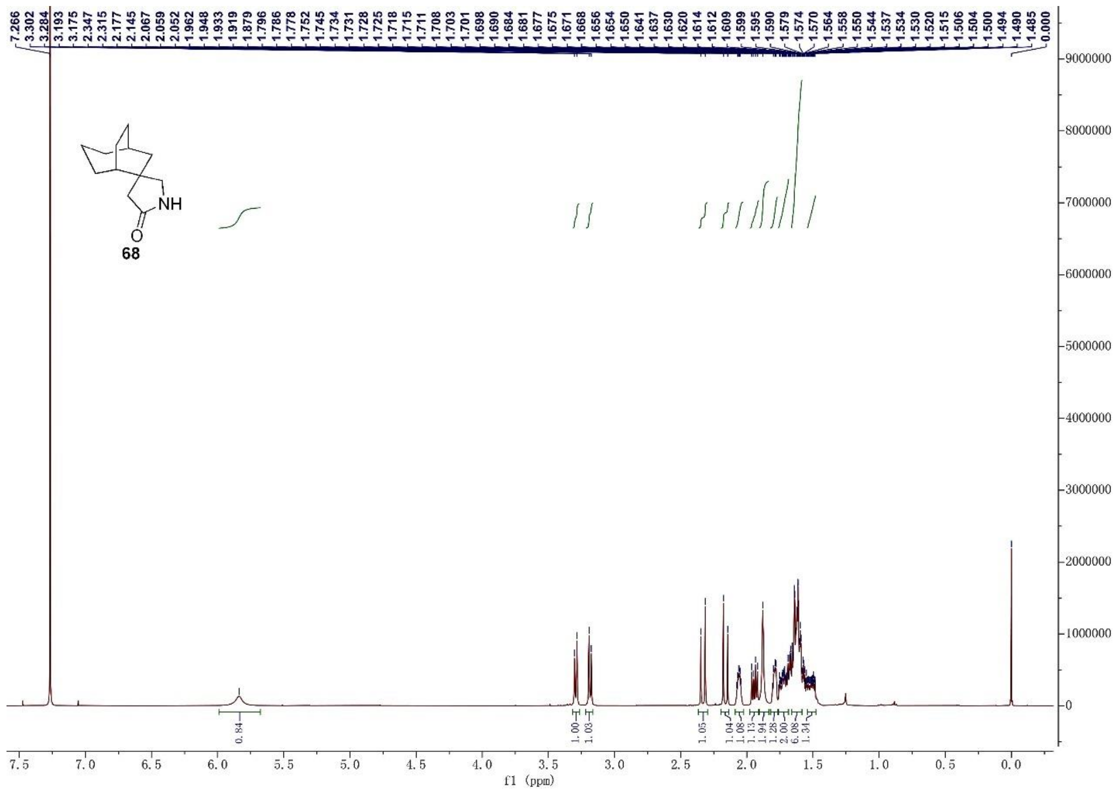

Figure S79. ¹H NMR spectrum of **68** (500 MHz, $CDCl_3$)

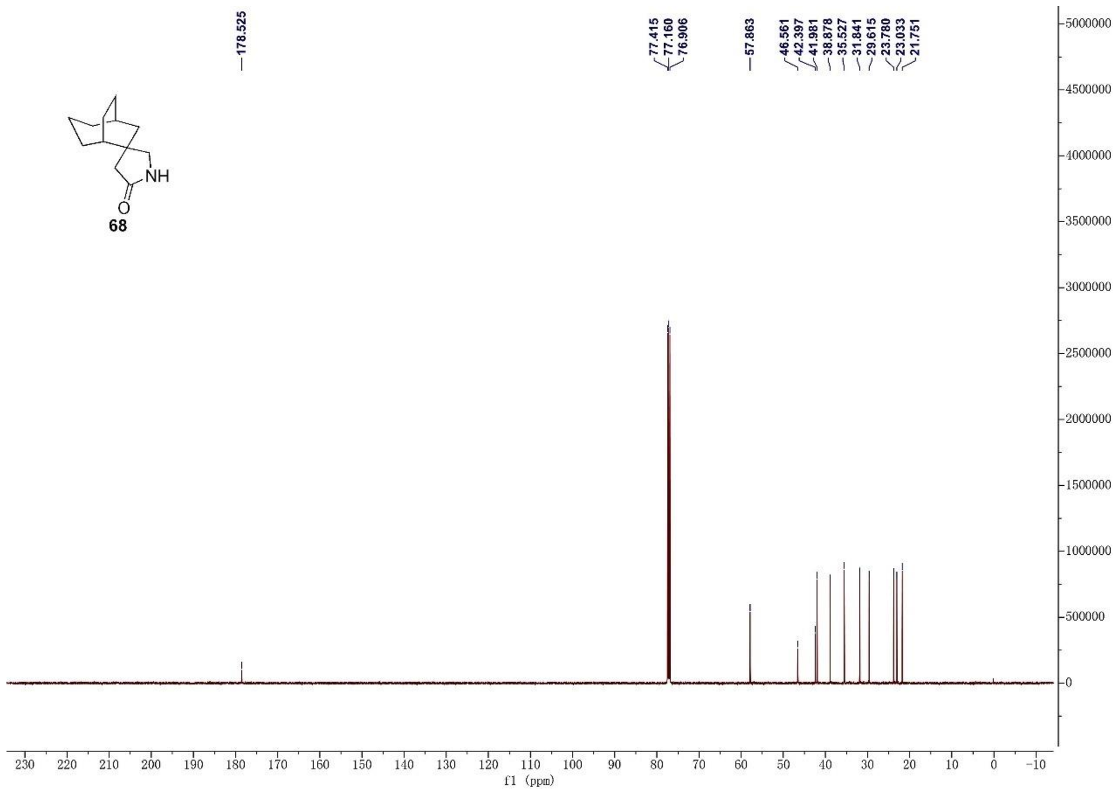

Figure S80. ^{13}C NMR spectrum of **68** (126 MHz, $CDCl_3$)

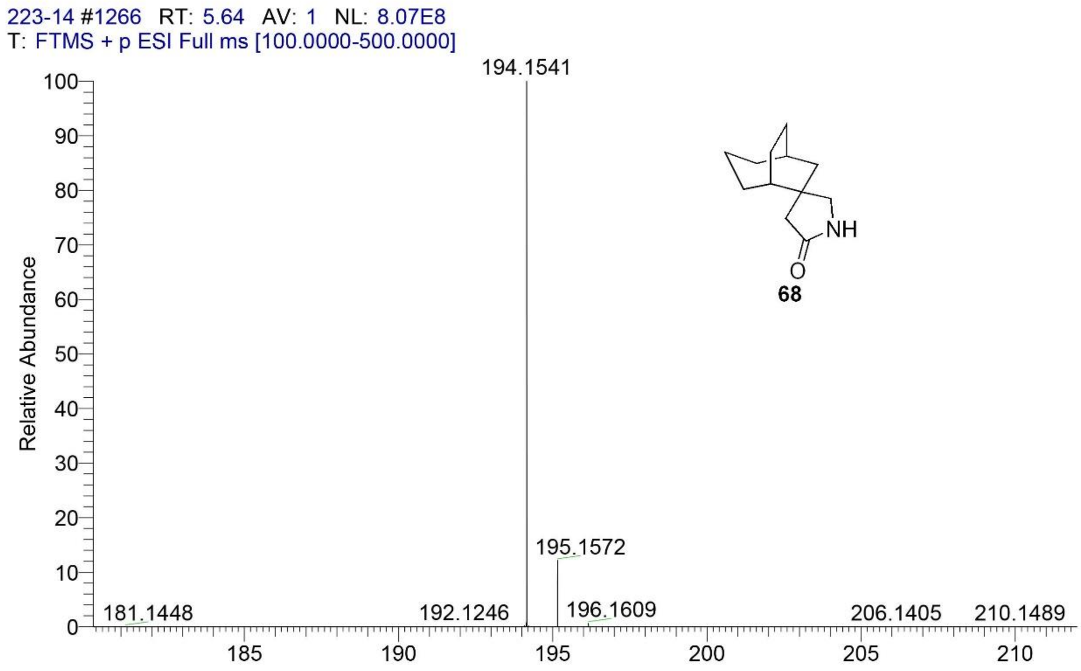

Figure S81. HR-MS spectrum of **68**

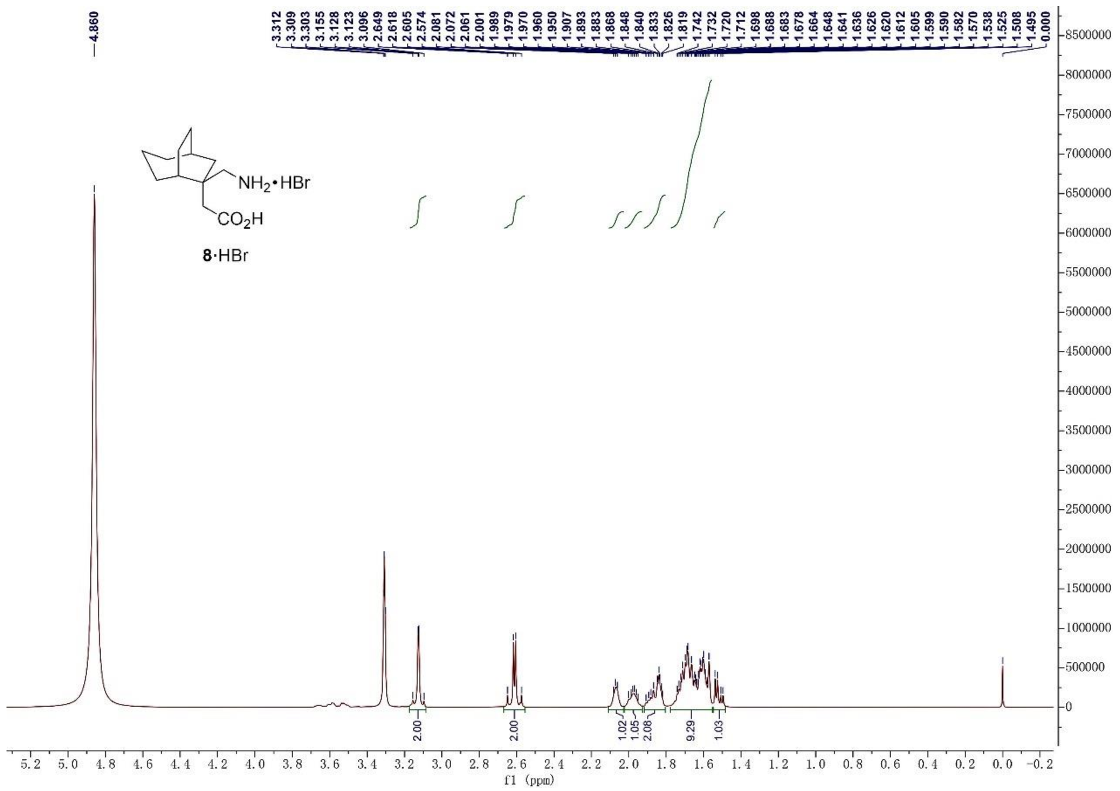

Figure S82. ^{1}H NMR spectrum of **8•**HBr (500 MHz, CD_3OD)

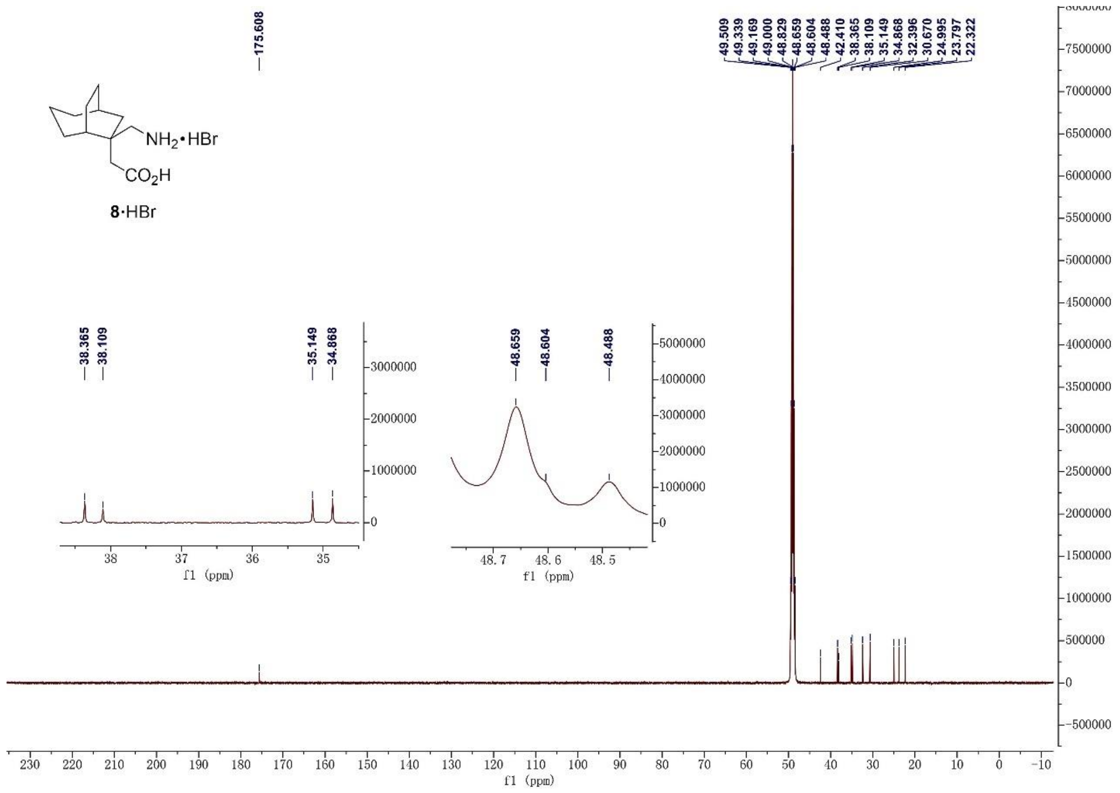

Figure S83. ^{13}C NMR spectrum of **8•**HBr (126 MHz, CD_3OD)

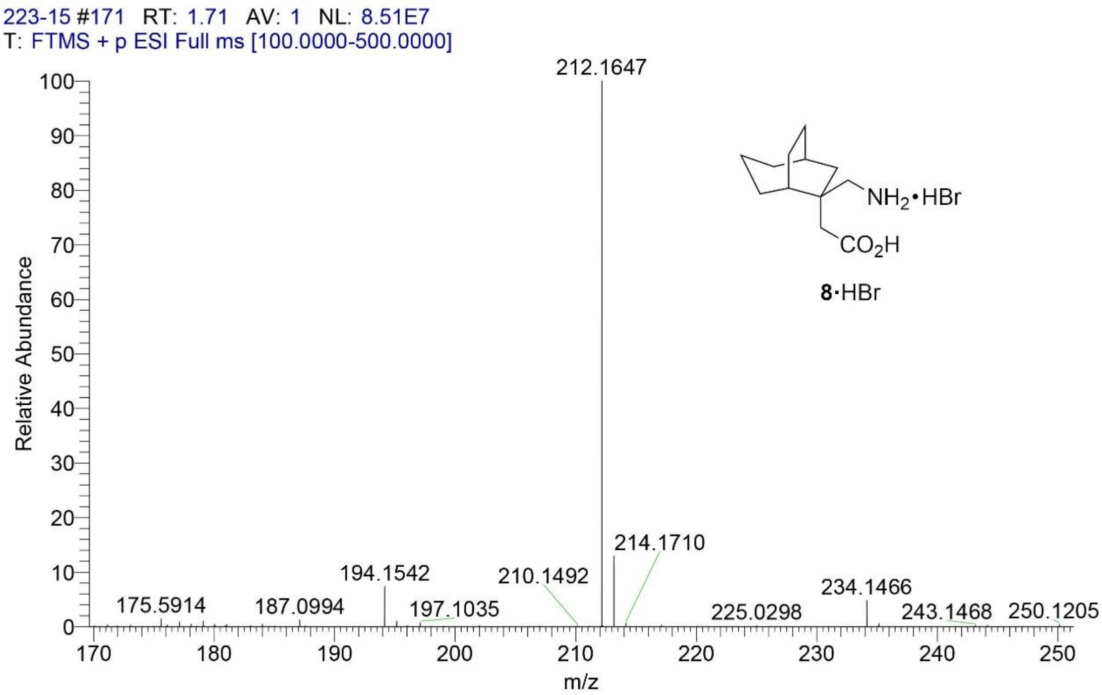

Figure S84. HR-MS spectrum of **8•HBr**

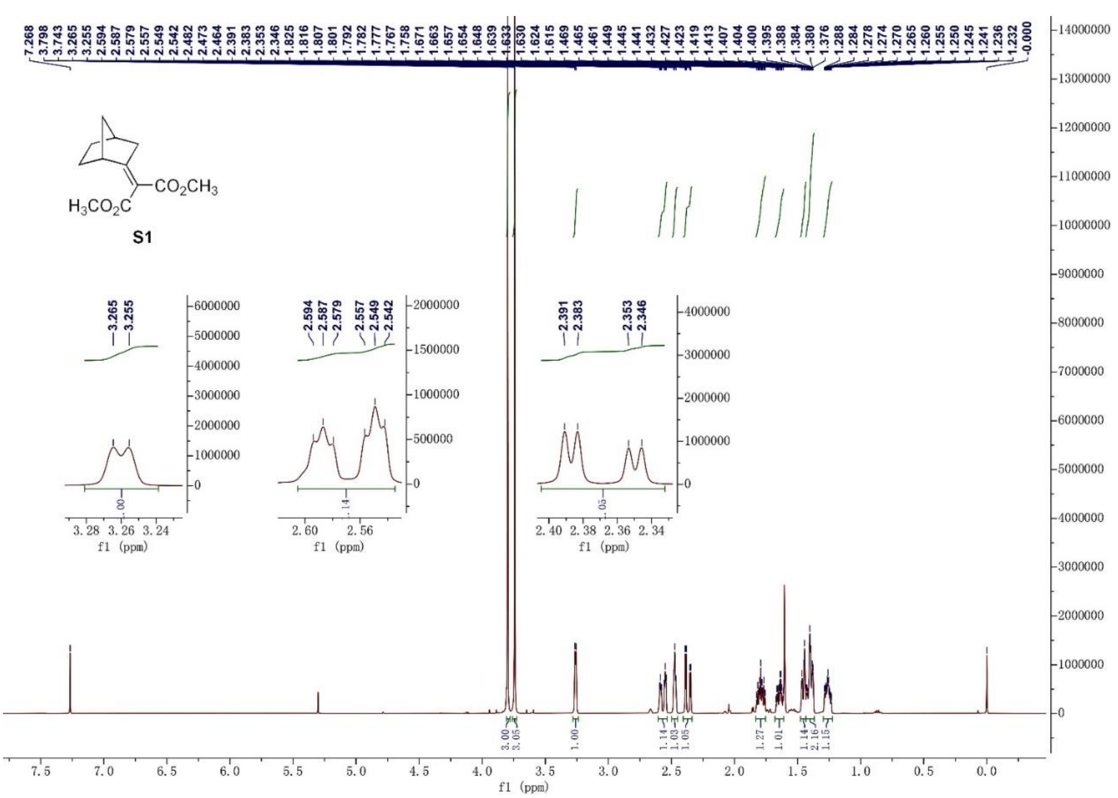

Figure S85. ^{1}H NMR spectrum of **S1** (500 MHz, $CDCl_3$)

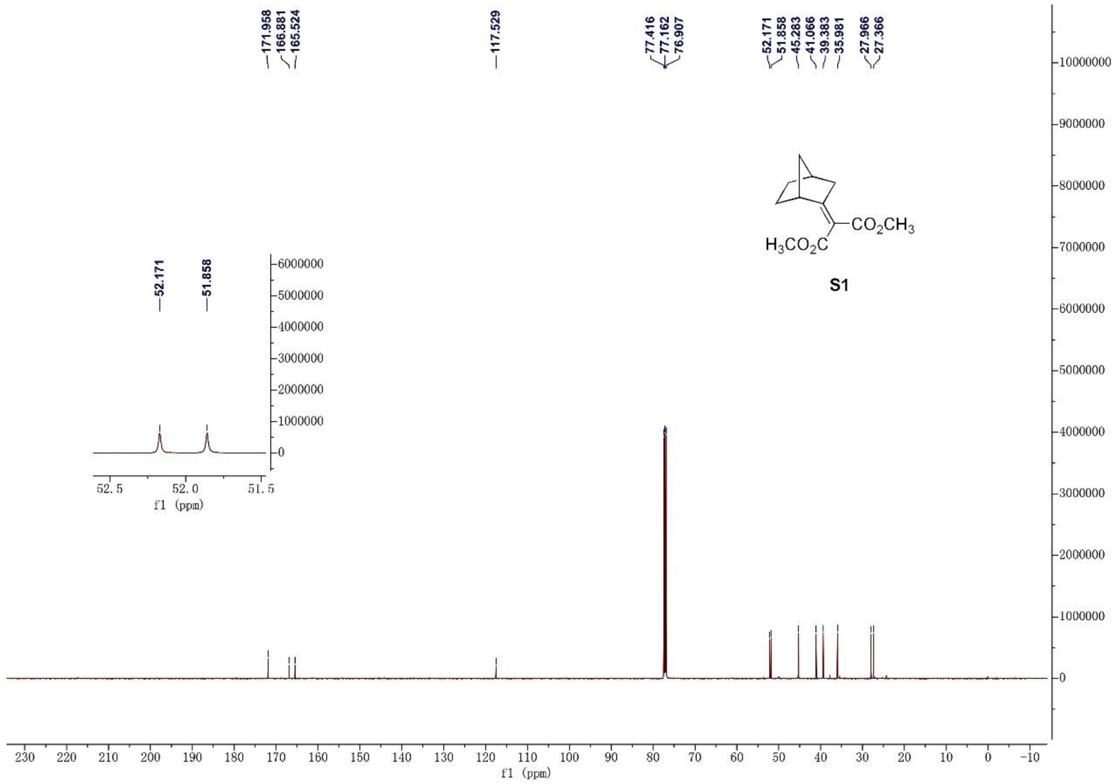

Figure S86. ^{13}C NMR spectrum of **S1** (126 MHz, $CDCl_3$)

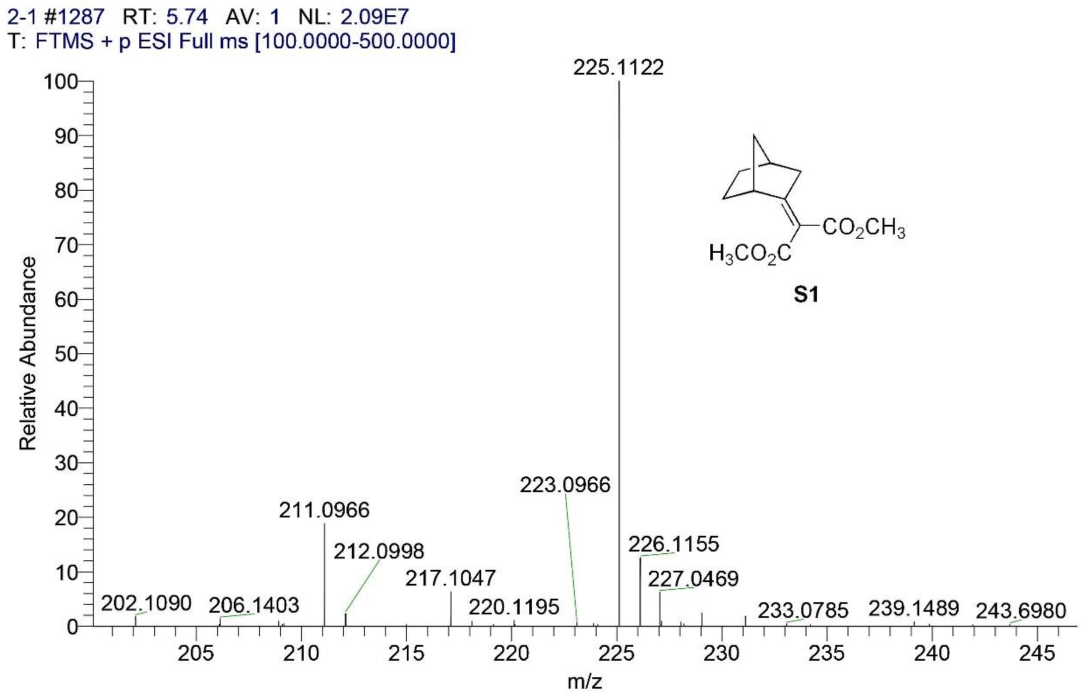

Figure S87. HR-MS spectrum of **S1**

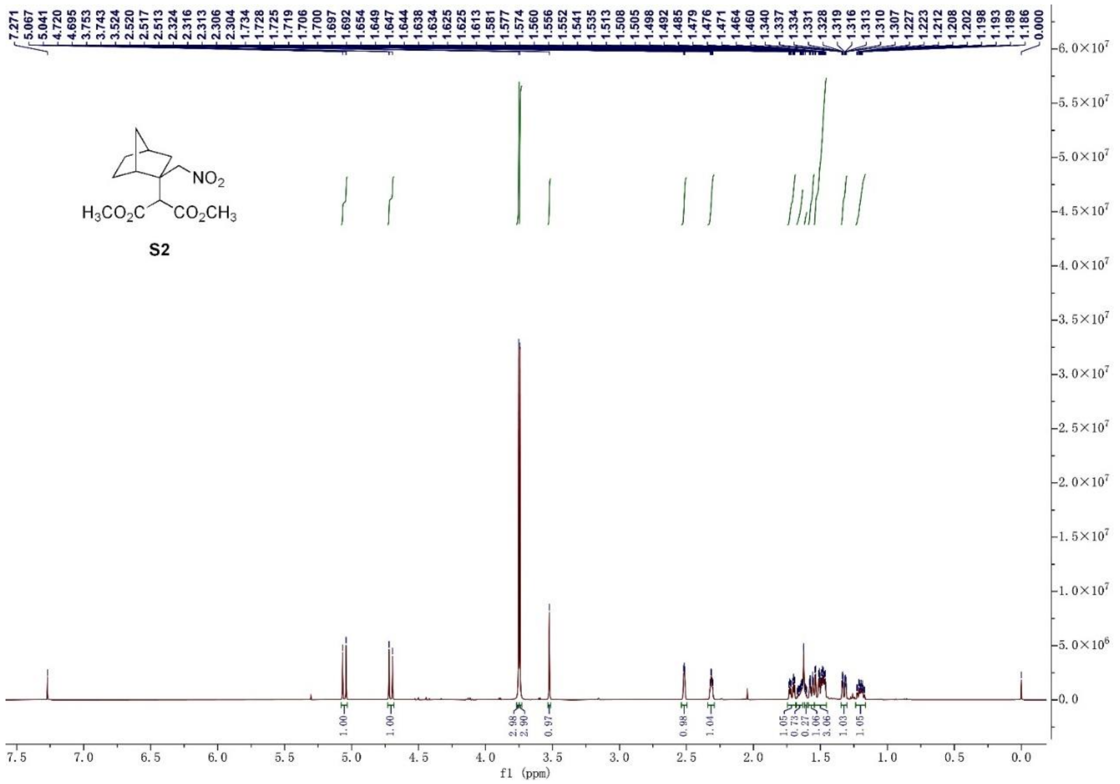

Figure S88. ^{1}H NMR spectrum of **S2** (500 MHz, $CDCl_3$)

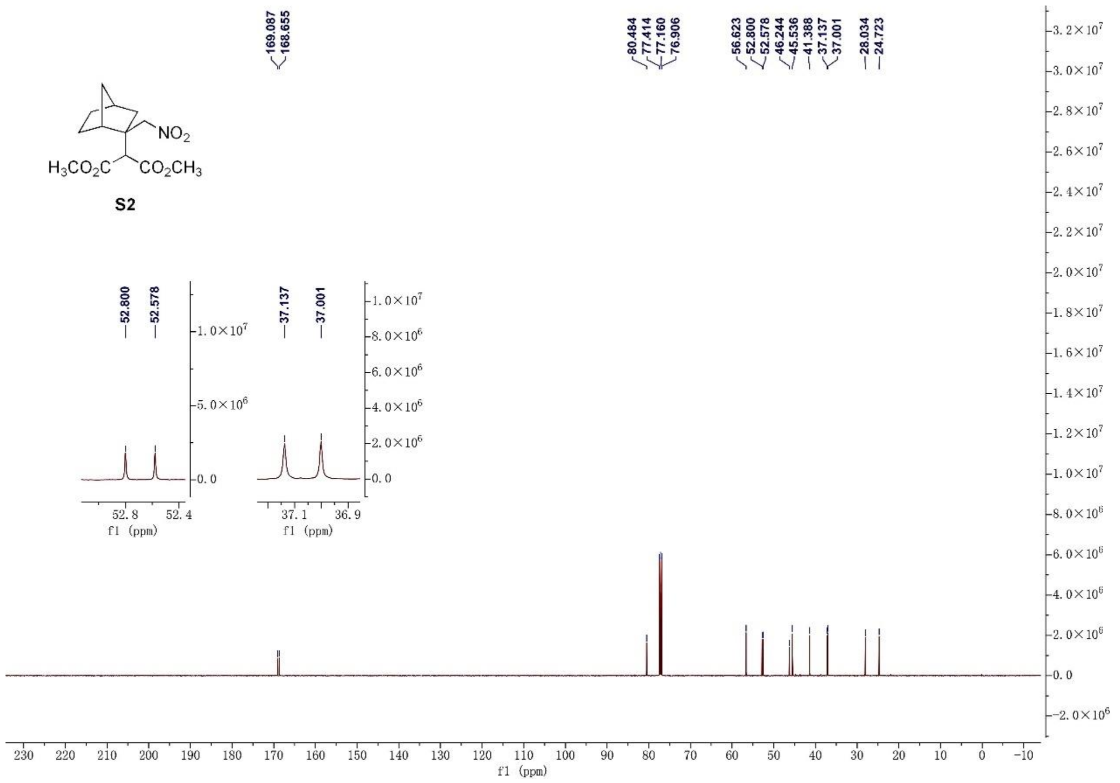

Figure S89. ^{13}C NMR spectrum of **S2** (126 MHz, $CDCl_3$)

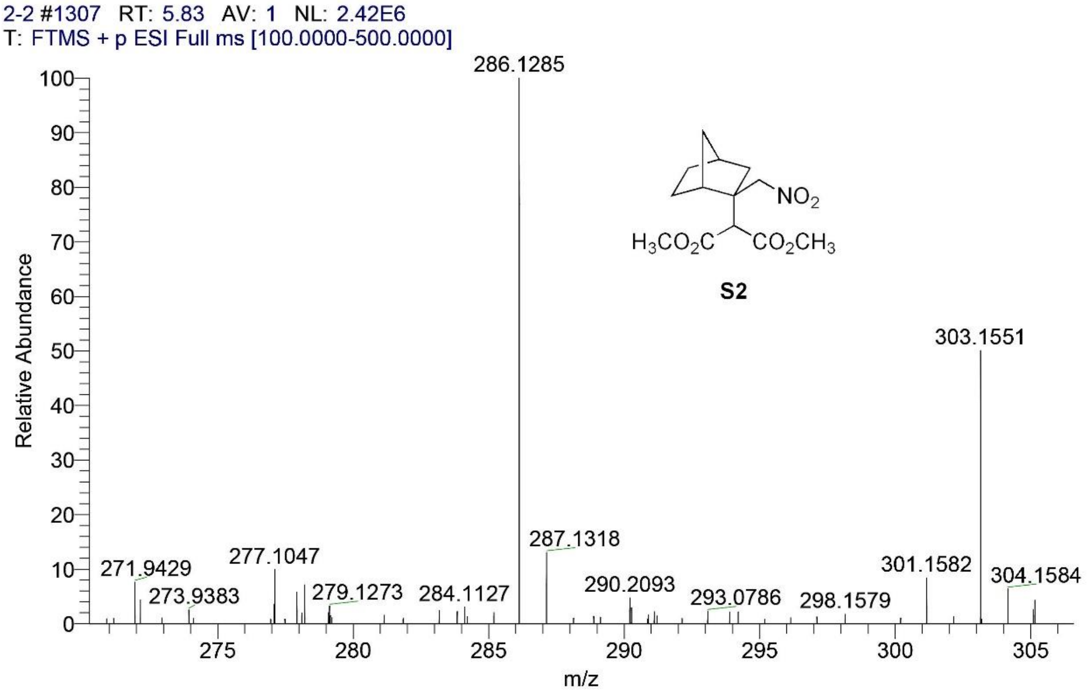

Figure S90. HR-MS spectrum of **S2**

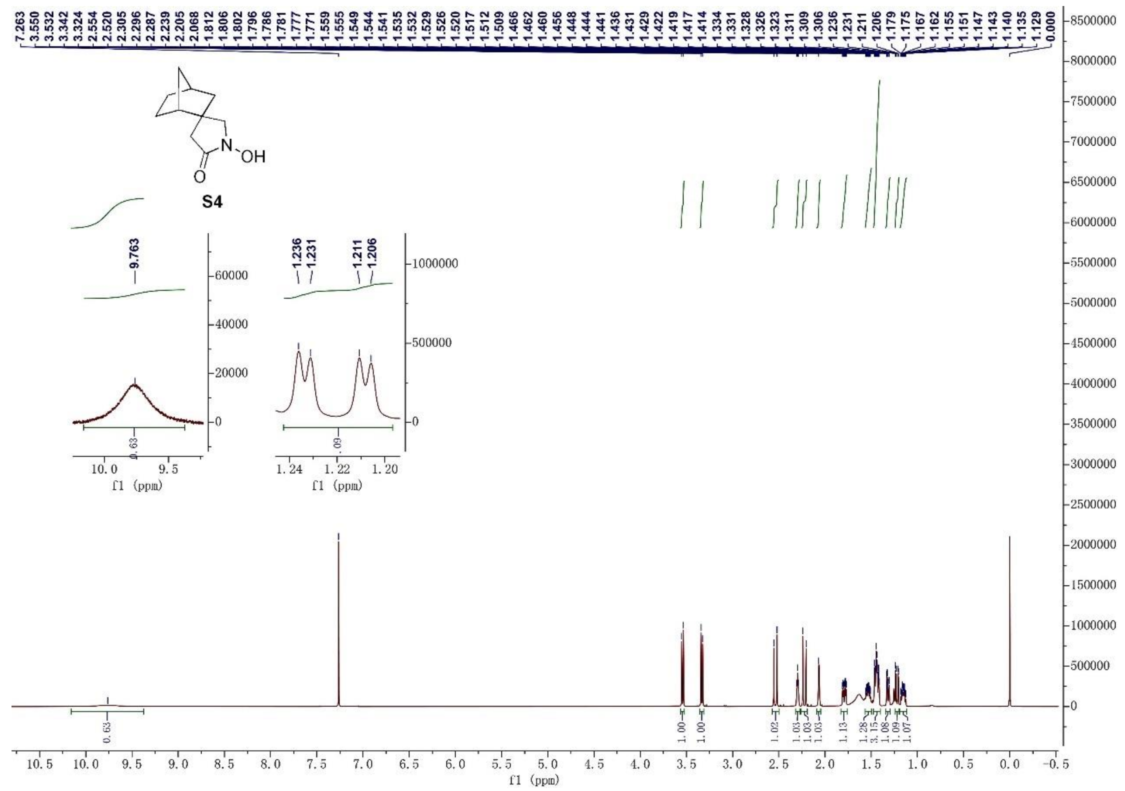

Figure S91. ¹H NMR spectrum of **S4** (500 MHz, $CDCl_3$)

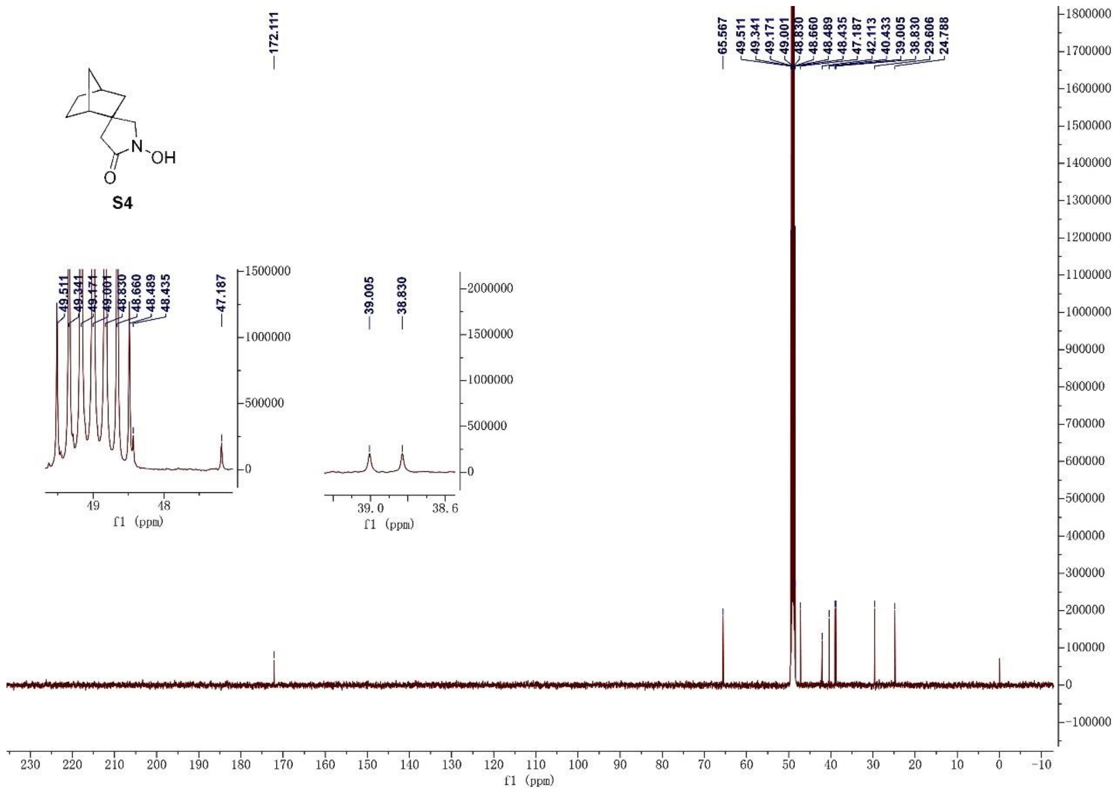

Figure S92. ^{13}C NMR spectrum of **S4** (126 MHz, CD_3OD)

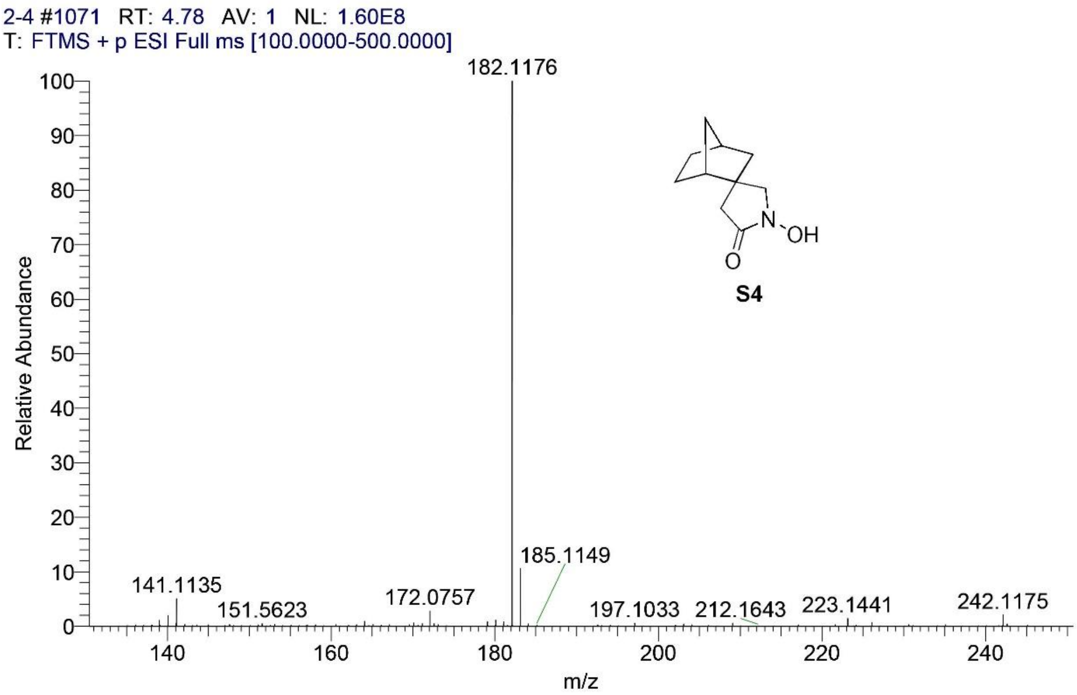

Figure S93. HR-MS spectrum of **S4**

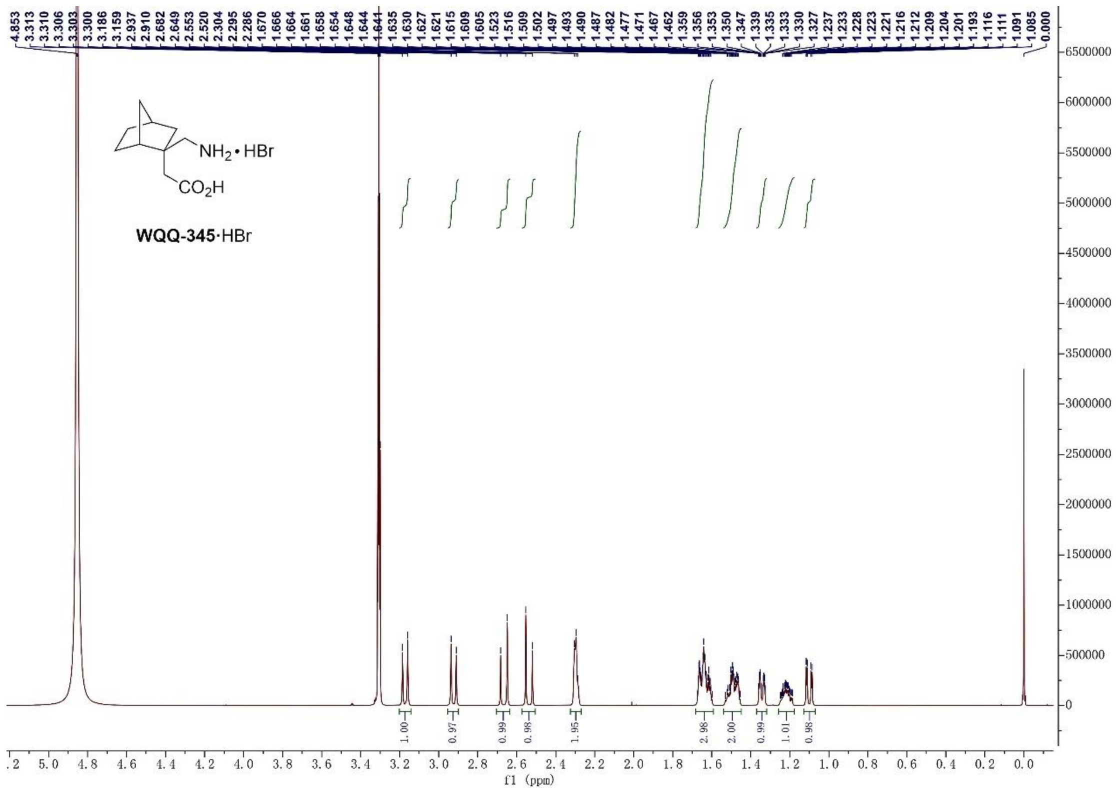

Figure S94. ^{1}H NMR spectrum of **WQQ-345**•HBr (500 MHz, CD_3OD)

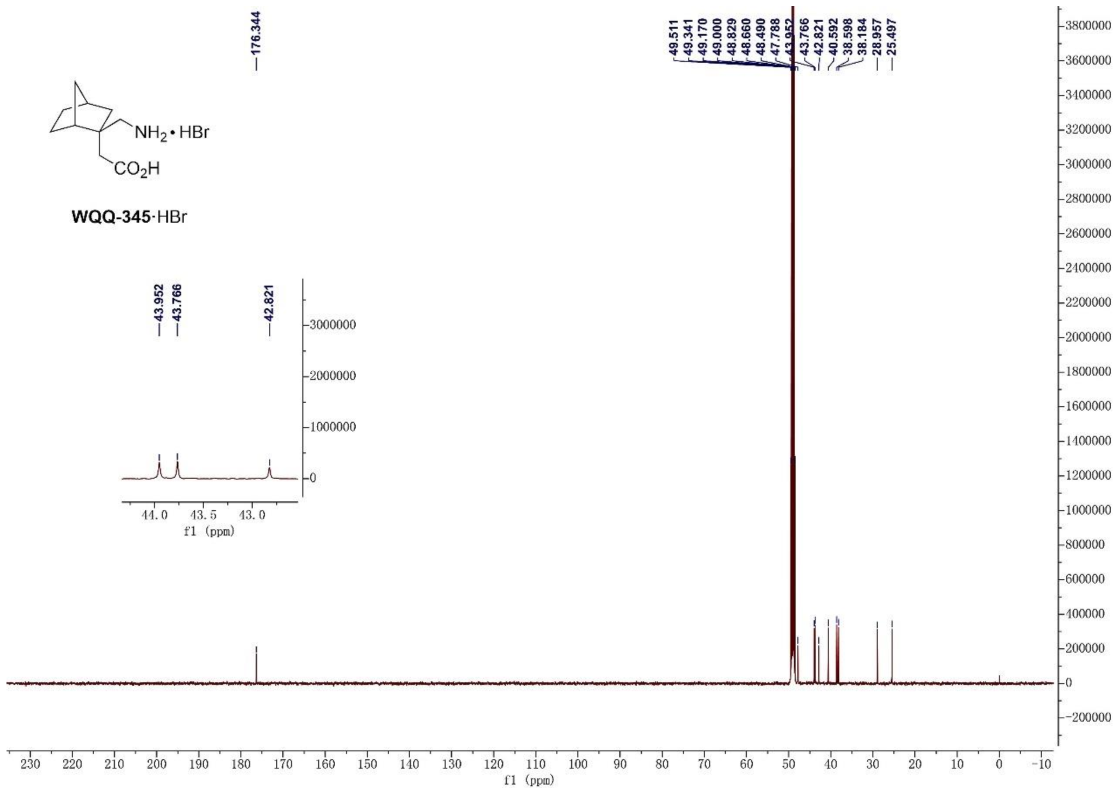

Figure S95. ^{13}C NMR spectrum of **WQQ-345**•HBr (126 MHz, CD_3OD)

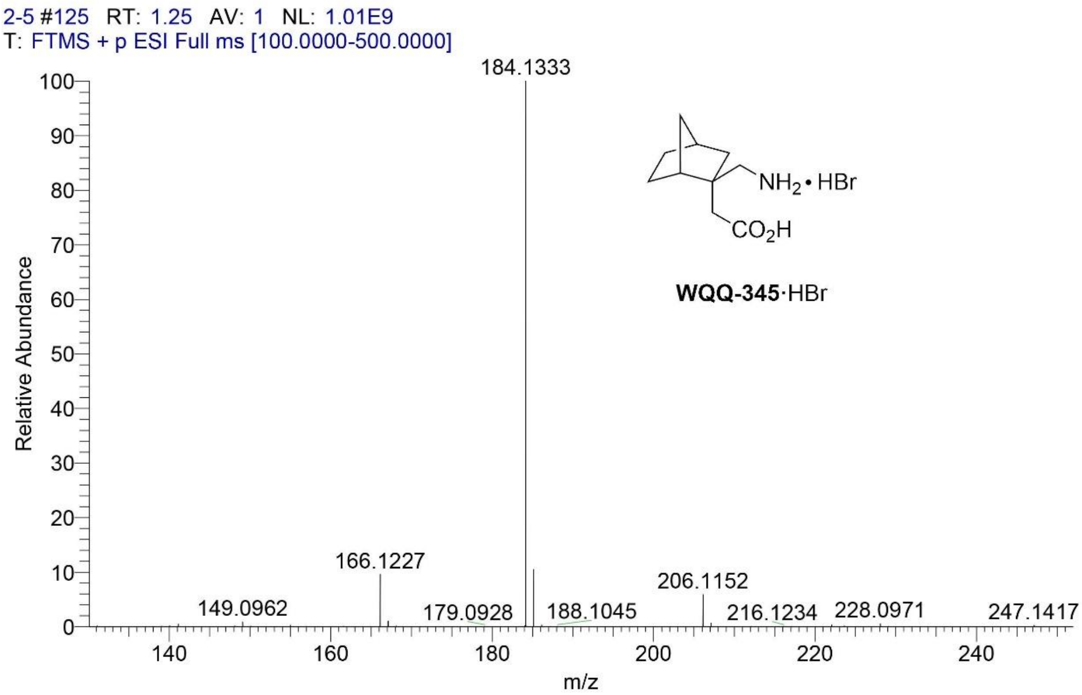

Figure S96. HR-MS spectrum of **WQQ-345**•HBr
